# Structural Variety, Luminescent Properties, and DFT Calculations of 2‑Naphthaldehyde Thiosemicarbazone and Its Complexes with Zinc(II) and Cadmium(II)

**DOI:** 10.1021/acs.inorgchem.6c02677

**Published:** 2026-09-15

**Authors:** Alfonso Castiñeiras, Isabel García-Santos, Lorenzo Seijo, Julio Corredoira-Vázquez, Sergi Burguera, Antonio Frontera

**Affiliations:** † Department of Inorganic Chemistry, Faculty of Pharmacy, University of Santiago de Compostela, 15782 Santiago de Compostela, Spain; ‡ Departamento de Química Inorgánica, Facultade de Química, 16780 Universidade de Santiago de Compostela, 15782 Santiago de Compostela, Spain; § Institute of Materials (iMATUS), Universidade de Santiago de Compostela, 15782 Santiago de Compostela, Spain; ∥ Departament de Química, 16745 Universitat de les Illes Balears, Crta e Valldemossa km 7.5, 07122 Palma de Mallorca (Baleares), Spain

## Abstract

A 2-naphthyl functionalized thiosemicarbazone has been synthesized in high yield by means of a Schiff base condensation reaction. The 2-(naphthalen-2-ylmethylene)­hydrazinecarbothioamide (Hnatsc, **1**) reacts with zinc­(II) and cadmium­(II) chlorides and acetates to obtain neutral complexes of formulae: [ZnCl_2_(Hnatsc)_2_]·H_2_O (**2**·H_2_O), [Zn­(natsc)_2_]·3H_2_O (**3**·3H_2_O), [Zn­(natsc)_2_(Hnatsc)_2_]·3H_2_O (**4**·3H_2_O), [Zn­(OAc)­(natsc)­(Hnatsc)]·2H_2_O (**5**·2H_2_O), [CdCl_2_(Hnatsc)_2_] (**6**), [Cd­(natsc)_2_] (**7**), and [Cd­(OAc)­(natsc)­(MeOH)] (**8**), in which the thiosemicarbazone functions as a monodentate thione ligand or as a deprotonated bidentate chelating ligand coordinating to the metal ions via the imine nitrogen and thiolate sulfur atoms. The characterization of all compounds was through a combination of analytical techniques, including elemental analysis, nuclear magnetic resonance (NMR) spectroscopy, infrared (IR) spectroscopy, and electrospray ionization (ESI) mass spectrometry. The molecular and crystal structures of Hnatsc and complexes **2** and **6** were determined by means of single-crystal X-ray diffraction. Furthermore, a fluorescence study was conducted on the thiosemicarbazone and the complexes. A density functional theory (DFT) study using MEP, QTAIM, and NCIplot analyzed the supramolecular architecture, revealing that metal coordination significantly strengthens hydrogen-bonded synthons. Furthermore, TD-DFT calculations provided insight into the photophysical properties, confirming the ligand-centered nature of the electronic transitions and identifying high internal conversion rates as the primary quenching mechanism in the zinc complex **2**.

## Graphical abstract

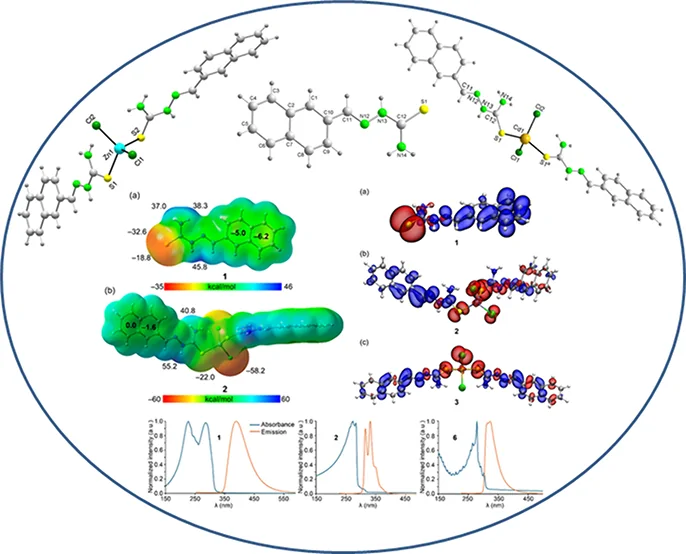

## 1 Introduction

It is evident that a multitude of aromatic hydrocarbons, including but not limited to naphthalene, anthracene, and pyrene, in conjunction with their derivatives, are characterized by the presence of a delocalized π system. This feature has garnered significant attention in the realm of optoelectronic applications, a testament to its stability and fluorescent capacity.^1,2^ In this field of research, certain metal complexes containing conjugated π systems as ligands have recently found applications in many areas of chemistry and materials science. In this regard, some luminescent complexes can function as sensors in light devices.^3^ These semicarbazones are characterized by their π systems, which exhibit delocalized electrons along the chain. They contain one sulfur atom and one nitrogen atom as donors, and are hypothesized to be effective in the formation of metal complexes that exhibit fluorescence. Luminescent coordination compounds with *N,S*-chelate ligands as thiosemicarbazones are of interest due to their favorable performance in sensor techniques and luminescent devices. A significant number of *d* ^10^ metal complexes, with a particular emphasis on zinc­(II) and cadmium­(II) complexes with nitrogenous ligands and thiosemicarbazones, have been synthesized and their luminescent behavior has been thoroughly examined.^4,5^

It is clear that these semicarbazones, which possess the functional group >N–N­(H)–C­(S)-N<, represent a class of molecules whose chemistry constitutes an interdisciplinary field. In this respect, they play an important role in various scientific disciplines, including coordination chemistry, materials chemistry, biochemistry and bioinorganic chemistry. The biological activity of thiosemicarbazone derivatives has been a major focus in the field of chemistry for some time,^6,7^ and it is well established that many of them possess remarkable biological properties such as antifungal,^8^ antitumor^9^ or anti-inflammatory.^10^ The utilization of coordination compounds comprising thiosemicarbazones as ligands is also relevant to the domain of diagnostic imaging and theranostics.^11^ Furthermore, certain complexes with thiosemicarbazones are used as precursors in the synthesis of nanostructured materials using thermal decomposition techniques, such as some metal ions in oxidation state II that have been used in the synthesis of ZnS,^12^ CdS,^13^ or CoS.^14^ Several theoretical and experimental studies have been conducted on thiosemicarbazones as organic corrosion inhibitors.^15,16^ In summary, the applications of thiosemicarbazones, either directly or through their derivatives, are numerous, highlighting their coordinating capacity towards various metal centers where they exhibit their donor variability.^17^

Regarding the aforementioned points, there has recently been a growing interest in research on the photochemistry of luminescent Zn­(II) and Cd­(II) coordination complexes, both in their monomeric forms,^18–20^ as well as in multinuclear forms^21,22^ or as polymeric species.^23–26^ Divalent zinc and cadmium ions are colorless *d* ^10^ systems. In the construction of luminescent complexes, they exhibit tetrahedral, square pyramidal, trigonal bipyramidal, or octahedral coordination geometries and can be used as electroluminescent materials, such as organic light-emitting diodes (OLEDs).^27^

In accordance with the aforementioned considerations and in light of the extant literature, this study reports the synthesis of a ligand derived from 2-naphthaldehyde and thiosemicarbazide. The extended aromatic system of this ligand has the potential to provide both supramolecular recognition sites and significant luminescent properties.

The coordination chemistry of 2-(naphthalen-2-ylmethylene)­hydrazinecarbothioamide (Hnatsc, Chart 1) and the products of its reaction with zinc­(II) and cadmium­(II) chlorides and acetates are also reported. The new compounds were fully characterized by means of elemental analysis, infrared spectroscopy, ^1^H, ^13^C, and ^113^Cd (where solubility permitted) nuclear magnetic resonance spectroscopy, mass spectrometry, and single-crystal X-ray diffraction of the thiosemicarbazone (**1**) and the complexes with ZnCl_2_ (**2**) and CdCl_2_ (**6**). Furthermore, an investigation was conducted into the luminescent properties of Hnatsc and all the complexes in solution. Beyond the experimental investigation of luminescent properties, a theoretical study was undertaken to deepen the understanding of the system. DFT calculations, including MEP surface analysis, QTAIM, and NCIplot, were employed to quantify the strength of noncovalent interactions and the effect of metal coordination on supramolecular synthons. Additionally, TD-DFT studies were performed to elucidate the electronic nature of the absorption and emission bands and to rationalize the observed variations in fluorescence quantum yields.

**I cht1:**
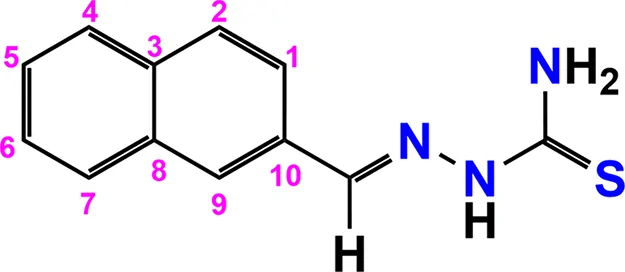
2-(Naphthalen-2-ylmethylene)­hydrazinecarbothioamide (Hnatsc, **1**), with Atom Labels for NMR Analysis

## 2 Experimental Section

### 2.1 Physical Measurements

All reagents and solvents were commercial products that were used without further purification. Melting points were determined using a Büchi M-560 melting point apparatus and are uncorrected. Microanalyses (C, H, N, and S) were performed using a Thermo Finnigan model. Flash EA 1112 elemental analyzer. IR spectra were recorded as KBr discs (4000–400 cm^–1^) using a Bruker IFS-66v spectrophotometer, and as Nujol mulls (500–100 cm^–1^) using a Varian 670-IR spectrophotometer. Conductivity measurements of 10^–3^ M solutions of the complexes in DMF were taken with a Mettler Toledo mod. Seven Easy Conductivity Meter. Mass spectra were derived using a Micromass AutoSpec Premier mass spectrometer with a nitrobenzyl alcohol matrix and ion-exchange (IE) technique for the complexes, and an ESI technique with a Bruker MicroTOF analyzer. ^1^H, ^13^C­{^1^H}, and ^113^Cd NMR spectra were obtained from DMSO-d6 solutions using a Bruker AMX 300 spectrometer. For the ^113^Cd spectra, a 0.5 M solution of reagent-grade Cd­(ClO_4_)_2_ was used as an external standard. Correlation (HMQC and HMBC) spectra were recorded using a Varian INOVA 400 spectrophotometer. UV-vis spectra of 10^–4^ M solutions in MeOH were recorded using a JASCO V-630 spectrophotometer with a 1 cm quartz cell in the 250–600 nm range. Emission and excitation spectra of the complexes were obtained using a JASCO FP-8300 spectrofluorometer for 10^–5^ M solutions.

### 2.2 Synthesis of 2-(Naphthalen-2-ylmethylene)­hydrazinecarbothioamide (Hnatsc)

The ligand synthesis was carried out in accordance with the general procedure for preparation via condensation of an aldehyde (or ketone) with a thiosemicarbazide, followed by elimination of a water molecule.^28^ Specifically, 2.88 g (31 mmol) of thiosemicarbazide (purchased from Sigma-Aldrich) was dissolved in 30 mL of distilled water at 65 °C in a 250 mL flask. Then, a solution of 4.97 g (32 mmol) of 2-naphthaldehyde (purchased from Sigma-Aldrich) in 160 mL of ethanol was added dropwise using a pressure-balanced funnel. The resulting mixture was then subjected to a reflux process accompanied by continuous stirring for 4 h. The colorless solid obtained was then separated by gravity filtration and dried under vacuum. Slow evaporation of the mother liquor over several days yielded crystals suitable for X-ray diffraction analysis. The compound is soluble in ethanol, methanol, acetonitrile, DMF, and DMSO.

#### 2.2.1 Data for Hnatsc (**1**)

Yield: 6.9 g (95.8 %). m.p.: 232 °C. Elemental analysis Found: C, 62.4; H, 4.5; N, 18.2; S, 13.3. Calc. for C_12_H_11_N_3_S (229.30): C, 62.9; H, 4.8; N, 18.3; S, 14.0 %. IR (ν_max_/cm^–1^): ν­(NH): 3384; 3253; 3155; ν­(CN): 1601; 1541; ν­(NN): 1065; ν­(CS): 822. IE MS [m/z, assignment]: 229.07 [C_12_H_11_N_3_S]^+^. ^1^H NMR (DMSO-*d*_6_, ppm): 11.48 (1H,*s*,NH); 8.20 (1H,*s*,NH_2_); 8.18 (1H,*s*,CH); 8.15 (0.5H,*d*,C9H); 8.12 (0.5H,*d*,C9H); 8.10 (1H,*s*,H1); 8.07 (1H,*s*,NH_2_); 7.88 (3H,*m*, H2+H4+H7); 7.51 (2H,*m*,H5+H6). ^13^C­{^1^H} NMR (DMSO-*d*_6_, ppm): 178.42 (C12); 142.76 (C11); 134.06 (C10); 133.27 (C3); 132.45 (C8); 129.26 (C1); 128.65 (C2); 128.15 (C4); 128.15 (C7); 127.41­(C5); 127.07 (C6); 123.54 (C9).

### 2.3 Synthesis and Crystallization of Complexes

The general procedure involves synthesizing the complexes by slowly adding a methanolic solution of the Hnatsc ligand (**1**) to a metal salt dissolved in the same solvent, with a metal-to-ligand ratio of either 1:1 or 2:1. In some syntheses, 1 mL of Et_3_N is added. The mixture is then stirred for approximately 1 week. The resulting solids are then filtered, washed with methanol and dried under vacuum.

Data for [ZnCl_2_(Hnatsc)_2_]·H_2_O (**2**·H_2_O): Hnatsc (0.20 g, 0.87 mmol), methanol (120 mL), ZnCl_2_ (0.12 g, 0.87 mmol), methanol (30 mL). White solid. Yield: 0.198 g (36.78%). m.p.: 240.5 °C. Elemental analysis Found: C, 47.05; H, 3.8; N, 13.14; S, 10.41. Calc. for C_24_H_24_Cl_2_N_6_OS_2_Zn (610.01): C, 47.21; H, 3.97; N, 13.77; S, 10.48. %. IR (ν_max_/cm^–1^): ν­(NH): 3384; 3253; 3155; ν­(CN): 1601; 1541; ν­(NN): 1065; ν­(CS): 821; ν­(Zn–S): 353; ν­(Zn–Cl): 247. IE MS [m/z, assignment]: 521.07 [Zn­(natsc)_2_]^+^; 365.29 [ZnCl_2_(Hnatsc)]^+^; 750.12 [Zn­(natsc)­(Hnatsc)_2_]^+^. ^1^H NMR (DMSO-*d*_6_, ppm): 11.52 (2H,*s*,NH); 8.25 (2H,*s*,NH_2_); 8.22 (2H,*s*,CH); 8.18 **(**1H,*d*,H9**)**; 8.16 **(**1H,*d*,H9**)**; 8.11 (2H,*s*,H1); 8.09 (2H,*s*,NH_2_); 7.92 (6H,*m*,H2+H4+H7); 7.52 (4H,*m*,H5+H6). ^13^C­{^1^H} NMR (DMSO-*d*_6_, ppm): 178.38 (C12); 142.85 (C11); 134.06 (C10); 133.28 (C3); 132.46 (C8); 129.24 (C1); 128.64 (C2); 128.15 (C4); 128.15 (C7); 127.41 (C5); 127.08 (C6); 123.54 (C9).

Colorless, needle-shaped crystals suitable for the single-crystal X-ray analysis of the formula [ZnCl_2_(Hnasc)_2_] (**2**) were obtained by slowly evaporating the mother liquor at room temperature.

#### 2.3.1 Data for [Zn­(natsc)_2_]·3H_2_O (**3**·3H_2_O)

Hnatsc (0.20 g, 0.87 mmol), methanol (25 mL). Zn­(OAc)_2_ (0.08 g, 0.44 mmol), methanol (20 mL). Yellow solid. Yield: 0.19 g (76.67%). m.p.: 240.5 °C. Elemental analysis Found: C, 50.67; H, 3.98; N, 14.79; S, 11.28. Calc. for C_24_H_26_N_6_O_3_S_2_Zn (574.08): C, 50.17, H, 4.56; N, 14.63; S, 11.14. IR (ν_max_/cm^–1^): ν­(NH): 3500; 3372; ν­(CN): 1600; 1575; ν­(NN): 1035; ν­(CS): 745; ν­(Zn–N): 464; ν­(Zn–S): 355. IE MS [m/z, assignment]: 521.40 [Zn­(natsc)_2_]^+^; 292.28 [Zn­(natsc)]^+^. ^1^H NMR (DMSO-*d*_6_, ppm): 8.32 (2H,*s*,CH); 8,12 (1H,*d*,H9); 8.10 (1H,*d*,H9); 7.84, (4H,*t*,H4+H7); 7,72 (2H,*s*,H1); 7.49 (4H,*s*,NH2); 7.46-7.33 (6H,*m*,H2+H5+H6). ^13^C­{^1^H} NMR (DMSO-*d*_6_, ppm): 178.47 (C12); 145.03 (C11); 134.16 (C10); 133.63 (C3); 133.36 (C8); 128.43 (C1); 128.12 (C2); 128.04 (C4); 127.96­(C7); 126.71­(C5); 126.71 (C6); 123.76 (C9).

#### 2.3.2 Data for [Zn­(natsc)_2_(Hnatsc)_2_]·3H_2_O (**4**·3H_2_O)

Hnatsc (0.20 g, 0.87 mmol), methanol (25 mL), ZnCl_2_ (0.12 g, 0.87 mmol), methanol (20 mL), Et_3_N (1 mL). Yellow solid. Yield: 0.095 g (10.57%). m.p.: 240.5 °C. Elemental analysis Found: C, 55.76; H, 4.39; N, 16.33; S, 12.45. Calc. for C_48_H_48_N_12_O_3_S_4_Zn (1032.21): C, 55.80; H, 4.69; N, 16.28; S, 12.39. IR (ν_max_/cm^–1^): ν­(NH): 3383; 3250; 3153; ν­(CN): 1600; 1540; ν­(NN): 1066; ν­(CS): 817; ν­(Zn–N): 452; ν­(Zn–S): 353. IE MS [m/z, assignment]: 521.05 [Zn­(natsc)_2_]^+^; 750.12 [Zn (natsc)_2_(Hnatsc)]^+^. ^1^H NMR (DMSO-*d*_6_, ppm): 11.52 (2H,*s*,NH); 8.22 (4H,*s*,CH); 8.17 (2H,*d*,H9); 8.15 (2H,*d*,H9); 8.11 (2H,*s*,H1); 8.04 (8H,*s*,NH_2_); 7.92 (12H,*m*,H2+H4+H7); 7.52 (8H,*m*,H5+H6). ^13^C­{^1^H} NMR (DMSO-*d*_6_, ppm): 178.38 (C12); 142.85 (C11); 133.94 (C10); 133.31 (C3); 132.31 (C8); 128.90 (C1); 128.57 (C2); 128.50 (C4); 128.12 (C7); 127.20­(C5); 126.98 (C6); 123.61 (C9).

#### 2.3.3 Data for [Zn­(OAc)­(natsc)­(Hnatsc)]·2H_2_O (**5**·2H_2_O)

Hnatsc (0.20 g, 0.87 mmol), methanol (25 mL), Zn­(OAc)_2_ (0.08 g, 0.44 mmol), methanol (20 mL), Et_3_N (1 mL). White solid. Yield: 0.11 g (38.63%). m.p.: 238 °C. Elemental analysis Found: C, 50.05; H, 4.33; N, 14.07; S, 11.12. Calc. for C_26_H_28_N_6_O_4_S_2_Zn (616.09): C, 50.64; H, 4.58; N, 13.64; S, 10.38. IR (ν_max_/cm^–1^): ν­(NH): 3496; 3381; 3254; 3155; ν­(CN): 1601; 1541; CO: 1574; 1477; ν­(NN): 1065; ν­(CS): 745; ν­(Zn–N): 451; ν­(Zn–O): 412; ν­(Zn–S): 356. IE MS [m/z, assignment]: 571.40 [Zn­(CH_3_O_2_)­(Hnatsc)_2_]^+^; 340.41 [Zn­(CH_3_O_2_)­(natsc)]^+^; 799.20 [Zn­(CH_3_O_2_)­(natsc)­(Hnatsc)_2_]^+^. ^1^H NMR (DMSO-*d*_6_, ppm): 11.52 (1H,*s*,NH); 8.25 (2H,*s*,NH_2_); 8.25 (2H,*s*,CH); 8.17 (1H,*d*,H9); 8.15 (1H,*d*,H9); 8.10-7.92 (6H,*m*,H1+NH_2_); 7.91 (6H,*m*,H2+H4+H7); 7.53 (8H,*m*,H5+H6); 1.86 (3H,*s*,OAc). ^13^C­{^1^H} NMR (DMSO-*d*_6_, ppm): 178.19 (C12); 178.04 (C=O_OAC_); 142.59 (C11); 133.47 (C10); 132.79 (C3); 128.99 (C8); 128.48 (C1); 128.08 (C2); 128.04 (C4); 127.62 (C7); 126.76­(C5); 126.50 (C6); 123.07 (C9); 31.51 (CH_3(OAc)_).

#### 2.3.4 Data for [CdCl_2_(Hnatsc)_2_] (**6**)

Hnatsc (0.20 g, 0.87 mmol), methanol (120 mL), CdCl_2_ (0.16 g, 0.87 mmol), methanol 30 mL. White solid. Yield: 0.217 g (36.78%). m.p 240.5 °C. Elemental analysis Found: C, 44.19; H, 3.25; N, 12.58; S, 9.85. Calc. for C_24_H_24_CdCl_2_N_6_OS_2_ (641.97): C, 44.90; H, 3.45; N, 13.08; S, 9.96. IR (ν_max_/cm^–1^): ν­(NH): 3440; 3267; 3176; ν­(CN): 1600; 1551; ν­(NN): 1063; ν­(CS): 822; ν­(Cd–S): 356; ν­(Cd–Cl): 246. IE MS [m/z, assignment]: 606.01 [CdCl­(Hnatsc)_2_]^+^; 377.94 [CdCl­(Hnatsc)]^+^; 836.07. [CdCl­(Hnatsc)_3_]^+^. ^1^H NMR (DMSO-*d*_6_, ppm): 11.51 (2H,*s*,NH); 8.24 (2H,*s*,NH2); 8.19 (1H,*s*,CH); 8.16 (1H,*d*,H9); 8.15 (1H,*d*,H9); 8.11 (2H,*s*,H1); 8.01 (2H,*s*,NH2); 7.92 (6H,*m*,H2+H4+H7); 7.54 (4H,*m*,H5+H6). ^13^C­{^1^H} NMR (DMSO-*d*_6_, ppm): 178.47 (C12); 145.03 (C11); 134.16 (C10); 133.63 (C3); 133.36 (C8); 128.42 (C1); 128.12 (C2); 128.04 (C4); 128.04 (C7); 127.97­(C5); 126.71 (C6); 123.76 (C9).

Some colorless, lamellar crystals with the formula [Cd­(Hnatsc)_2_Cl_2_·2DMSO (**6**·2DMSO)] formed in the NMR tube when the DMSO-*d*_6_ solution prepared for these studies was slowly evaporated at room temperature over a period of several days. These crystals are suitable for structural analysis by single-crystal X-ray diffraction.

#### 2.3.5 Data for [Cd­(natsc)_2_] (**7**)

Hnatsc (0.20 g, 0.87 mmol), methanol (25 mL), Cd­(OAc)_2_ (0.08 g, 0.44 mmol), methanol (20 mL). Yellow solid. Yield: 0.14 g (56.81%). m.p.: 240 °C. Elemental analysis Found: C, 51.35; H, 3.80; N, 15.08; S, 11.96. Calc. for C_24_H_20_CdN_6_S_2_ (570.02): C, 50.52; H, 3.54; N, 14.74; S, 11.22. IR­(ν_max_/cm^–1^): ν­(NH): 3499; 3447; 3371; ν­(CN): 1575; 1495; ν­(NN): 1037; ν­(CS): 819; ν­(Cd–N):451; ν­(Cd–S):355. IE MS [m/z, assignment]: 569.05 [Cd­(natsc)_2_]^+^; 340.78 [Cd­(natsc)]^+^. ^1^H NMR (DMSO-*d*_6_, ppm): 8.28 (2H,*s*,CH); 8.10 (1H,*d*,H9); 8.07 (1H,*d*,H9); 7.82 (4H,*t*,H4+H7); 7.75 (2H,*s*,NH2); 7.50 (1H,*s*,NH2); 7.42 (6H,*m*,H2+H5+H6). ^13^C­{^1^H} NMR (DMSO-*d*_6_, ppm): 178.54 (C12); 144.65 (C11); 133.96 (C10); 133.69 (C3); 133.37 (C8); 128.45 (C1); 128.20 (C2); 128.06 (C4); 128.06 (C7); 127.78 (C5); 127.78­(C6); 123.75­(C9).

#### 2.3.6 Data for [Cd­(OAc)­(natsc)­(MeOH)]·(**8**)

Hnatsc (0.2 g, 0.87 mmol), methanol (25 mL), Cd­(OAc)_2_ (0.11 g, 0.87 mmol), methanol (20 mL). Yellow solid. Yield: 0.28 g (74.71%). m.p 240.5 °C. Elemental analysis Found: C, 41.88; H, 4.08; N, 9.84; S, 7.73. Calc. for C_15_H_17_CdN_3_O_3_S (433.00): C, 41.57; H, 3.96; N, 9.70; S, 7.38. IR­(ν_max_/cm^–1^): ν­(NH): 3417; 3399; ν­(CN): 1574; 1464; CO: 1519; 1484; ν­(NN): 1034; ν­(CS): 819; ν­(Cd–N): 461; ν­(Cd–O): 419; ν­(Cd–S): 355. IE MS [m/z, assignment]: 398.29 [Cd­(OAc)­(natsc)]^+^; 798.78 [Cd­(OAc)­(natsc)]_2_^+^. ^1^H NMR (DMSO-*d*_6_, ppm): 8.20 (1H,*s*,CH); 8.05 (1H,*d*,C9H); 7.82; (3H, *t*, H2+H4+H7); 7.82 (1H, *d*, NH2); 7.43 (3H,*q*,H5+H6); 7.40 (1H,*s*,NH2); 3.13 (2H,*d*,MeOH); 1.81 (3H,*s*,OAc). ^13^C­{^1^H} NMR (DMSO-*d*_6_, ppm): 178.44 (C12); 178.23 (C=O_OAC_); 144.96 (C11); 134.14 (C10); 133.68 (C3); 133.44 (C8); 128.45 (C1); 128.31 (C2); 128.08 (C4); 127.91 (C7); 127.83­(C5); 126.78 (C6); 123.82 (C9); 58.21 (CH_3_OH); 31.51 (CH_3(OAc)_).

### 2.4 Crystal Structure Determination and Refinement

The diffraction data were obtained at 100(2) K using a Bruker SMART CCD 1000 diffractometer for Hnatsc and a Bruker X8 Kappa APEXII diffractometer for [ZnCl_2_(Hnatsc)_2_] and [CdCl_2_(Hnatsc)_2_]·2DMSO, with crystals mounted on glass fibers and using Mo Kα radiation (λ = 0.71073 Å). The data were processed using APEX2 software^29^ and were corrected for absorption using a multi-scan method.^30^ The structures were solved using direct methods,^31^ which revealed the positions of all non-hydrogen atoms. These were then refined using a full-matrix least-squares procedure with anisotropic displacement parameters.^32^ The [ZnCl_2_(Hnatsc)_2_] structure was refined as a twin with components of 0.39(3) and 0.61(3). The disorder of the CdCl_2_ atoms in [CdCl_2_(Hnatsc)_2_]·2DMSO was refined anisotropically in two positions with occupancies of 0.50/0.50. The Flack X parameter (absolute structure parameter) was calculated to be −0.07(3), providing strong evidence that the absolute structure has been correctly assigned.^33^ All hydrogen atoms were located using difference maps and the positions of the O–H and N–H hydrogen atoms were refined (the others were included as riding groups); the isotropic displacement parameters of the H atoms were constrained to 1.2/1.5 *U*_eq_ of the carrier atoms. Molecular graphics were generated using PLATON/ORTEP^34^ and DIAMOND.^35^ Crystal data, experimental details, and refinement results are summarized in Table 1.

**Table 1:** Crystal Data and Structure Refinement for 2-(Naphthalen-2-ylmethylene)­hydrazinecarbothioamide (Hnatsc, **1**), [ZnCl_2_(Hnatsc)_2_] (**2**), and [CdCl_2_(Hnatsc)_2_]·2DMSO (**6**·2DMSO)

Compound	**1**	**2**	**6**·2DMSO
Empirical formula	C_12_H_11_N_3_S	C_24_H_22_Cl_2_N_6_S_2_Zn	C_28_H_34_CdCl_2_N_6_O_2_S_4_
Formula weight	229.30	594.86	798.15
Crystal system	Monoclinic	Triclinic	Monoclinic
Space group	*C*2/*c*	*P*1̅	*C*2
Unit cell dimensions			
*a* / Å	15.091(3)	7.1940(5)	35.723(5)
*b* / Å	5.8077(10)	11.0792(8)	6.282(5)
*c* / Å	25.646(4)	16.5626(12)	7.345(5)
*α* / deg	90	102.284(4)	90
*β* / deg	91.407(2)	90.625(4)	98.114(5)
*γ* / deg	90	100.670(4)	90
Volume / Å^–3^	2247.0(7)	1265.84(16)	1631.8(17)
*Z*	8	2	2
Calc. density/Mg/m^3^	1.356	1.561	1.624
Absorp. coefc. / mm^–1^	0.262	1.373	1.126
*F*(000)	960	608	812
Crystal size	0.30 × 0.13 × 0.07	0.60 × 0.20 × 0.10	0.19 × 0.15 × 0.02
*θ* range / deg	1.589 – 26.352	1.260 – 26.440	2.303 – 26.369
Limiting indices / *h*,*k*,*l*	–18/18, 0/7, 0/32	–8/9, −13/13, −20/20	–44/44, −7/7, 0/9
Refl. collect/unique [*R*_int_]	2294/2294 [0.0498]	35274/35274 [0.0691]	3324/3324 [0.0557]
Completeness θ / deg (%)	25.242(100.0)	25.242(95.2)	25.240(99.9)
Max. /min transm.	1.000–0.943	1.000–0.676	1.000–0.852
Data/parameters	2294/145	35274/317	3324/205
Goodness-of-fit on *F* ^2^	1.044	1.046	1.081
Final *R* indices	*R*_1_ = 0.0345 *wR*_2_ = 0.0793	*R*_1_ = 0.0858 *wR*_2_ = 0.2166	*R*_1_ = 0.0523 *wR*_2_ = 0.0897
*R* indices (all data)	*R*_1_ = 0.0475 *wR*_2_ = 0.0848	*R*_1_ = 0.1009 *wR*_2_ = 0.2307	*R*_1_ = 0.0702 *wR*_2_ = 0.0967
Largest dif. peak/hole e·Å^–3^	0.232 / −0.219	1.925 / −2.151	0.586 / −0.654
*CCDC number*	2555695	2555696	2555697

### 2.5 Photophysical Measurements

The electronic spectra were recorded on a JASCO V-630 UV-vis Spectrophotometer with solute concentrations of approximately 10^–4^ M in methanol. The application of baseline correction was a requisite step in the analysis. Fluorescence spectra were measured using a Jasco FP-8300 Spectrofluorometer. Samples for fluorescence measurements were diluted until the maximum absorption was below 0.1, typically 0.05. Three independent scans were recorded and averaged. For the purpose of fluorescence emission measurements, blank solutions were measured in the same way and subsequently subtracted. Corrections for the spectral efficiency of the experimental setup were applied to fluorescence emission spectra. In order to take the wavelength-dependence of the excitation source (Xe lamp) into account, a photodiode reference detector was used for fluorescence excitation measurements. Fluorescence quantum yields were determined relative to quinine sulfate in 0.2 M H_2_SO_4_ (whereas the quantum yield was determined to be 0.546 at 298 K),^36^ the parameters in question are determined from the equation.^37^

$$\Phi =\frac{A_{S}\cdot F_{x}\cdot n_{x}^{2}}{A_{x}\cdot F_{S}\cdot n_{S}^{2}}\Phi_{S}$$

where *A*_*i*_ denotes the absorption at the excitation wavelength, *F*_*i*_ the integrated fluorescence intensity, *n*_*i*_ the refractive index of the solvent, and *Φ*_*S*_ the quantum yield of the fluorescence standard. Sub-indexes *i* = *X* or *S* refer to sample (ligand Hnatsc, and complexes **2** to **8**) or Standard, respectively.

### 2.6 Theoretical Methods

The geometries of the studied compounds were fully optimized at the RI-CAM-B3LYP^38^ -D4 ^39^ /def2-TZVP^40,41^ level of theory using the TURBOMOLE 7.7 ^42^ software package. The spectroscopical calculations were done using the ORCA 6.1.1 ^43^ software package at the same level of theory To calculate the theoretical spectra and theoretical radiative and non-radiative rates, the hessian files of the compounds were obtained with frequency calculations. For the calculation of the spectra, the Herzberg-Teller vibrational coupling effect was included.^44^ The combined Bader’s Quantum Theory of Atoms in Molecules^45^ and Non-covalent Interaction Plot (QTAIM-NCIplot) analysis was done using the AIMAll software.^46^ For the NCI study, the PBE0 functional was used.^47^

## 3 Results and Discussion

### 3.1 Synthesis and Characterization

The condensation reaction between 2-naphthaldehyde and thiosemicarbazide in a 1:1 molar ratio, in an ethanol/water mixture, under reflux, leads to the formation of the corresponding thiosemicarbazone 2-(naphthalen-2-ylmethylene)­hydrazinecarbothioamide (Hnatsc), in high yield, as a colorless solid soluble in acetonitrile, dimethylformamide and dimethyl sulfoxide, and partially soluble in ethanol and methanol. The characterization of this compound has been achieved through elemental analysis, spectroscopic methods and single-crystal X-ray diffraction. In accordance with the characteristics of the majority of thiosemicarbazones, the predominant form of Hntsc, both in solid and solution, is the thione tautomer. This assertion is substantiated by the absence of the ν­(S–H) band in the IR spectrum (2500-2600 cm^–1^)^48^ and the NNH signal of the thione form appearing at 11.5 ppm in the ^1^H NMR spectrum in DMSO.

The reactions of Hnatsc with zinc­(II) and cadmium­(II) chlorides and acetates yield a variety of complexes, the precise nature of which depends on the preparation method and the metal salts used. The newly synthesized complexes were characterized by means of elemental analysis, various solid-phase and solution spectral techniques, and X-ray diffraction of single crystals of compounds **2** and **6**, which were obtained by slow evaporation of the reaction mixtures at room temperature. The complexes are stable in air and soluble in DMF, DMSO, and other common organic solvents, including acetonitrile, methanol, and so on. The molar conductivity (Λ_M_) values of 10^–3^ mol L^–1^ solutions of the complexes in DMF at room temperature are less than 15 S cm^2^ mol^–1^, indicating non-electrolyte behavior.^49^

The electron impact mass spectrum (EI-MS) of Hnatsc (Figures 1 and S1) shows an intense peak corresponding to the molecular ion of the ligand (m/z = 229.07 g/mol, [C_12_H_11_N_3_S]^+^).

**Figure 1:**
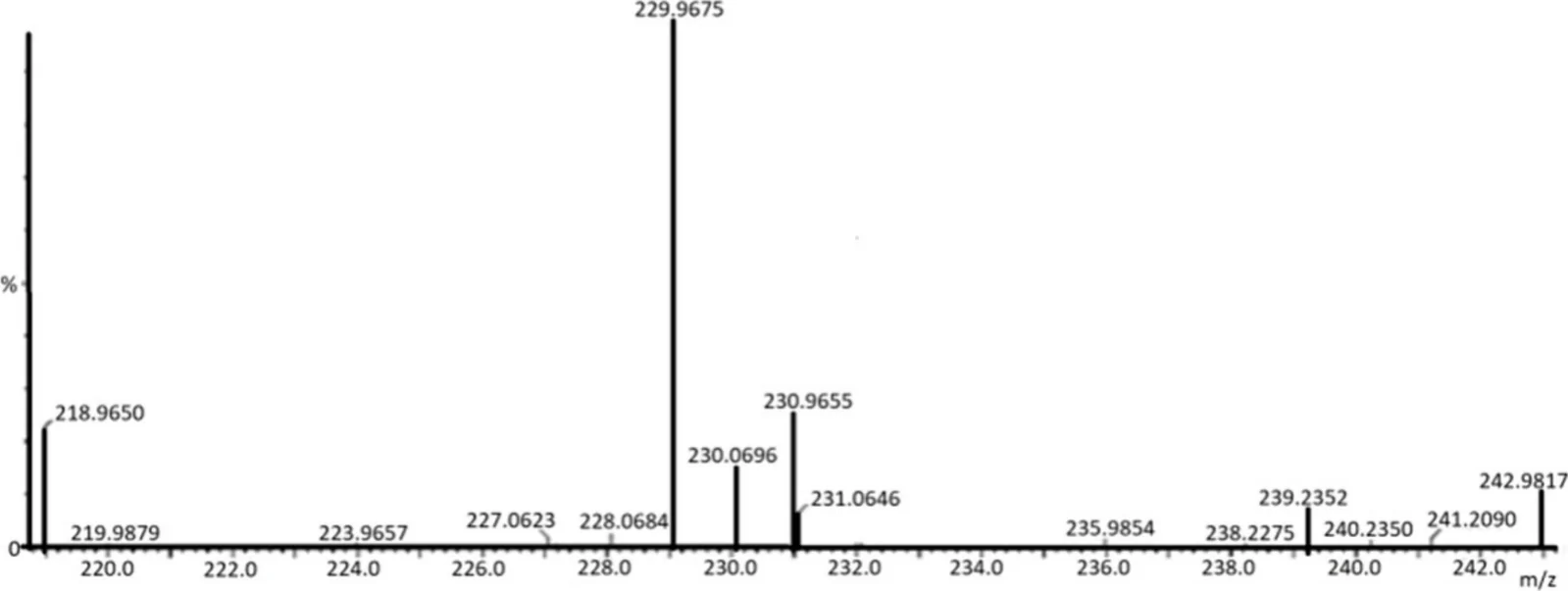
Hnatsc electron impact spectrum.

The presence of peaks corresponding to different fragments containing the neutral or deprotonated thiosemicarbazone in the electrospray-ionization mass spectra (ESI-MS) of the complexes indicates robust Zn–S and Cd–S bonds. In the spectra of complexes **2**–**5** and **7**, peaks are observed at the following m/z values: 521 for the Zn complexes and 569 for the Cd complex, which correspond to the [M­(natsc)_2_]^+^ fragment; and 522, which corresponds to [Zn (natsc)­(Hnatsc)]^+^. In complexes **3** and **7**, this coincides with the molecular ion. Furthermore, the presence of a peak at the m/z value of 750, which corresponds to [Zn­(natsc)_2_(Hnatsc)]^+^ or [Zn­(natsc)­(Hnatsc)_2_]^+^ in complexes **2**, **3**, and **5**, corroborates the aforementioned reasoning. Spectra of complexes containing chlorido or acetate ligands (**2**, **5**, **6**, and **8**) show peaks corresponding to anion fragments, such as [ZnCl_2_(Hnatsc)]^+^ (m/z = 365), [CdCl_2_(Hnatsc)_2_]^+^ (m/z = 606), and [Cd­(OAc)­(natsc)]^+^ (m/z = 398), among others (Figures S1–S5).

### 3.2 FT-IR Spectra

In the IR spectrum of Hnatsc, **1** (Figures 2, S6 and S7), the medium-intensity bands at 3384 and 3253 cm^–1^ are attributed to the asymmetric and symmetric stretching modes, respectively, of the −NH_2_ group, while another medium-intensity band at 3155 cm^–1^ is assigned to ν­(NH) of the -N-NH-C- group of the thiosemicarbazone fragment. A band due to the ν­(SH) modes may appear in the 2600–2550 cm^–1^ region. The absence of this band in the IR spectrum of **1** confirms that the thiosemicarbazone, in the solid state, exists in the thione form.

**Figure 2:**
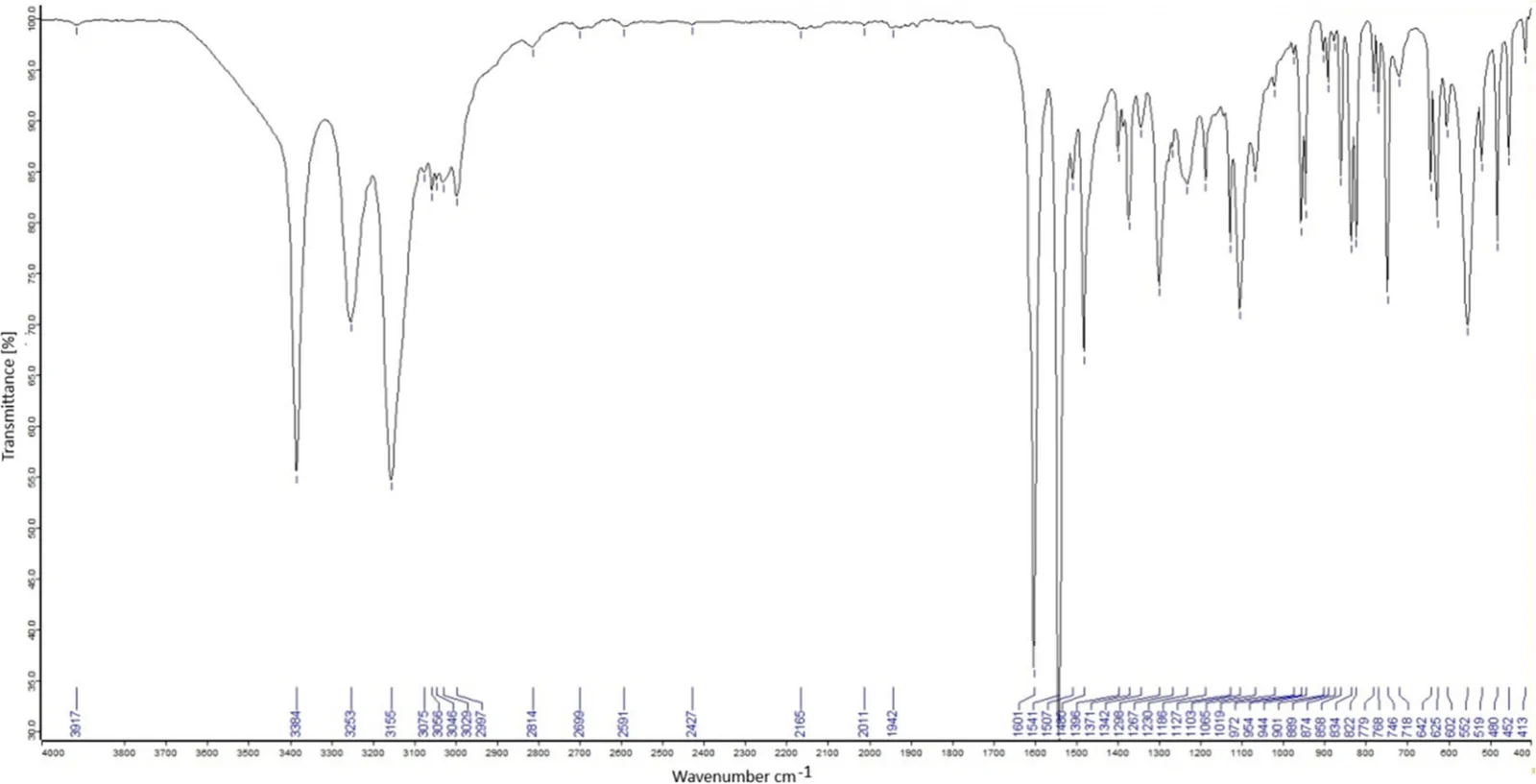
IR spectrum of Hnatsc in the range 4000-500 cm^–1^.

The complexity of the −C=N-NH-C­(=S)-NH_2_ system makes it difficult to assign the characteristic bands of the thiosemicarbazone fragment; however, in accordance with previous assignments for other thiosemicarbazones,^50^ three strong, sharp bands are observed in the 1600–1400 cm^–1^ range, which may be due to a combination of ν­(CN) + ν­(CC) and δ­(NH) + ν­(CN), or even a combination of all modes. Two bands at 1371 cm^–1^ and 1298 cm^–1^ can be assigned to a combination of ν­(CN) + ν­(CS). The middle band at 1019 cm^–1^ is probably due to ν­(NN) and, finally, the band that appears at 822 cm^–1^ may be mostly due to ν­(C=S).

In all the complex spectra (Figures S8–S21), most of the uncoordinated **1** bands are present, confirming the presence of the thiosemicarbazone as a ligand. In many of them, a broad band or shoulder is observed above 4500 cm^–1^, which is due to ν­(OH) from the H_2_O or CH_3_OH crystallization molecules. In compounds **2**, **4**, **5**, and **6**, the three bands assigned to the stretching modes of the −NH_2_ and −NH groups appear in the 3400–3100 cm^–1^ range, confirming that in these complexes, the thiosemicarbazone is in its neutral form. However, in complexes **3**, **7**, and **8**, the band at 3155 cm^–1^ of the Hnatsc spectrum has disappeared, indicating the deprotonation of the thiosemicarbazone and probably its coordination to the metal ion as a κ_2_-N,S chelate. In complexes **4** and **5**, where elemental analysis suggests the presence of [natsc]^−^, deprotonation is not observed due to the presence of neutral thiosemicarbazone molecules. In the 1560–1400 cm^–1^ region, in the spectra of complexes **3**, **6**, and **8**, the disappearance of the strong band at 1601 cm^–1^ and the shift of the bands in the uncoordinated ligand spectrum, which appear at 1541 and 1480 cm^–1^, to higher wavenumbers, seem to confirm the *κ* ^2^-N,S coordination of the thiosemicarbazone fragment. Furthermore, in many complexes, ν­(C=S) appears at lower wavenumbers.

The COO^–^ ion is present in complexes **5** and **8**. Acetate ligands are extremely flexible and exhibit versatile coordination modes, as each oxygen atom can act as a monodentate, chelating, monoatomic bridge, or triatomic bridge with syn-syn, syn-anti, and anti-anti configurations. These characteristics allow the formation of complexes with different nuclearities and a variety of topologies. According to an analysis of the structures found in the CSD, of acetates bonded to Zn­(II),^51^ the most common being monodentate (39.6%, Chart 2a), bidentate bridging (38.9%, Chart 2b), chelating (11.7%, Chart 2c) and monoatomic bridge (4.4%, Chart 2d). This implies different electron delocalization in the two C–O bonds, which is reflected in the position of the bands corresponding to the antisymmetric and symmetric stretching modes of these bonds. A study on the coordination mode of the acetate ion in complexes with a known molecular structure in relation to the IR frequency difference (Δν) between the asymmetric stretching modes, ν_a_(COO^–^), and symmetric stretching modes, ν_s_(COO^–^),^52^ concluded: a) if the acetate is ionic, Δν takes values between 160 and 175 cm^–1^; b) if it is a single-bidentate acetate, Δν generally takes values >200 cm^–1^; c) if Δν < 150 cm^–1^ the acetate must be bidentate chelate or bidentate bridge and d) the Δν values in bidentate bridge acetates are usually higher than those of chelates and often approach those of ionic acetates. Accordingly, the IR spectra of complexes **5** and **8** were analyzed and compared with the spectrum of uncoordinated Hnatsc in the 1600-1300 cm^–1^ region and it was observed that in complex **5** the ν­(COO^–^) bands appear centered at 1574 and 1344 cm^–1^ (Δν = 230 cm^–1^) which suggests a unidentate coordination for the acetate.^53^ However, in the IR spectrum of complex **8**, the bands assignable to ν­(COO^–^) are centered at 1574, 1464, and 1441 cm^–1^ (Δν = 110 and 133 cm^–1^) so that it is likely a bidentate chelate and bidentate bridging coordination of two different acetates, respectively.^54,55^

**II cht2:**
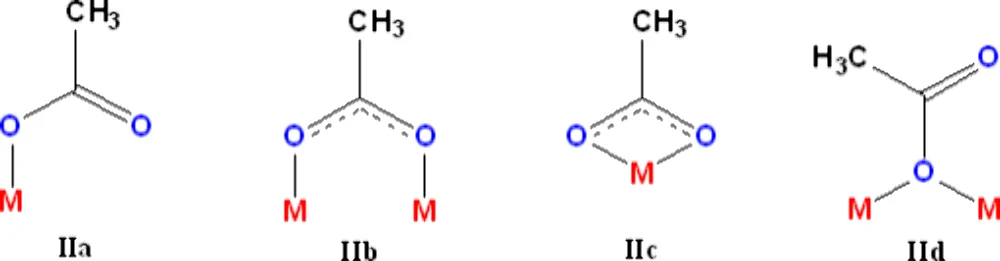
Analysis of the Main Coordination Modes of the Acetate Ion in Metal Complexes

The 500–100 cm^–1^ spectra of most complexes show two bands assigned, according to published data, to ν­(M-N_imino_) (340–370 cm^–1^) and ν­(M-S) (280–320 cm^–1^). The spectra of the zinc and cadmium complexes with acetate as ligand exhibit a band between 400 and 430 cm^–1^ that is absent in the other complexes and may be due to ν­(M-O),^56^ confirming the coordination of acetate in these complexes. The fact that the ν­(M-S) bands of complexes **2** and **6** are at lower wavenumbers than those of the other complexes is probably due to the M-S bond being weaker where the thiosemicarbazone is neutral than where it is deprotonated, forming a five-membered chelate ring. Finally, the bands that appear between 140 and 275 cm^–1^ in complexes **2** and **6** are attributed to ν­(M-Cl).^57^

### 3.3 NMR Spectra

In what follows, we will consider only those most important aspects related to the uncoordinated ligand and the modifications that some signals in the spectra undergo when it is coordinated. Figures S22–S31 show the ^1^H NMR spectra, and Figures S32–S38 show the ^13^C­{^1^H} NMR spectra from the DMSO-d^6^ solutions of thiosemicarbazone and its complexes.

Thiosemicarbazones have been known to exist in two isomeric forms viz. *E* or *Z*. On the basis of ^1^H NMR spectra data available in literature on heterocyclic thiosemicarbazones, it is possible to assign the correct conformations to the parent compounds.^58,59^ The diagnostic signal for this purpose is found to be the one belonging to the hydrazinic NH group which falls between δ = 13–15 ppm for the *E* form and at δ = 9–12 ppm for the *Z* form. The present thiosemicarbazone ligand displaying a singlet at 11.48 ppm obviously belongs to the former class in solution (Figure S22). This signal should disappear in the spectra of the complexes when thiolation of the ligand occurs and subsequent coordination through the deprotonated thiolate groups. In complexes **3**, **7**, and **8**, where the ligand is deprotonated, this deprotonation can be observed as the disappearance of the −NH signal (Figures S26, S30, and S31). As an example, Figure 3 compares the spectra of [ZnCl_2_(Hnatsc)_2_]·and [Zn­(natsc)_2_]·2H_2_O, where this deprotonation is observed.

**Figure 3:**
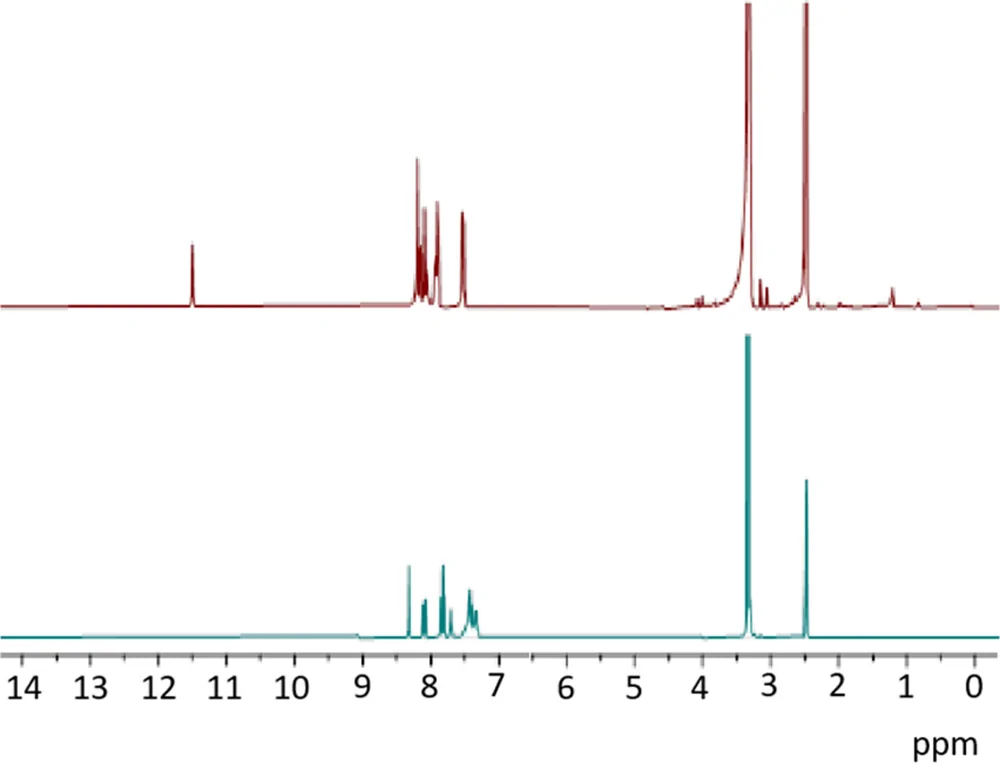
Comparative study of the ^1^H NMR spectra of [ZnCl_2_(Hnatsc)_2_] (top) and [Zn­(natsc)_2_]·2H_2_O (bottom), showing whether the ligand is neutral or deprotonated.

This study also reveals that while two distinct signals for the −NH_2_ group are observed at room temperature, at 8.19 and 8.12 ppm, which is attributed to the hindered rotation of the C–NH_2_ group, a single signal indicative of free rotation of this group appears at an increased temperature (Figure S23).

In the ^13^C NMR spectra, the carbons were assigned using HMQC and HMBC two-dimensional NMR spectroscopy. In the HMQC spectrum (Figure S24), it is also clearly observed that the H9 protons correlate with a single carbon (C9), indicating that this carbon does not fall within the shielding cone of the C=N group. In the ^13^C Hnatsc NMR spectrum, the most unshielded signal corresponds to the C=S thiocarbonyl group, appearing at 178.32 ppm, followed by the C=N carbon at 142.83 ppm, while the ring signals appear in the 123–134 ppm range typical of aromatic rings. In the complexes, there are hardly any shifts in most of the signals. As a consequence of ligand coordination, the signal that undergoes the greatest shift is that corresponding to the imine carbon (C11), since in all the complexes it is shifted to a lower field than in the ligand. For example 142.76 ppm in Hnatsc, **1** (Figure S32) and 144.65 ppm in [Cd­(natsc)_2_], **7** (Figure S37).

The chemical shifts of ^113^Cd are highly sensitive to the nature of the donor atoms, as well as to the coordination number, geometry, and solvent used, with the size of the halogen being the most determining factor in the signal position.^60–62^ Although the complexes are quite soluble in DMSO, the ^113^Cd NMR spectrum could only be recorded for the [Cd­(natsc)_2_] complex, which exhibits a single signal at 216.12 ppm, indicative of a single metal center (Figure 4). The absence of observable resonance in the ^113^Cd NMR spectra of complexes **6** and **8** can be attributed not to solubility in DMSO-d^6^, but to intrinsic limitations of NMR. The detectability of ^113^Cd resonance is limited by the relatively low sensitivity of the experiment, as well as by the relaxation properties and signal broadening associated with the coordination environment, resulting in very weak or unobservable signals even after long acquisition times. In addition, multiple structural factors, such as the nature and number of donor atoms and the coordination geometry, may also have contributed to the absence of observable resonances for complexes **6** and **8**.^63^

**Figure 4:**
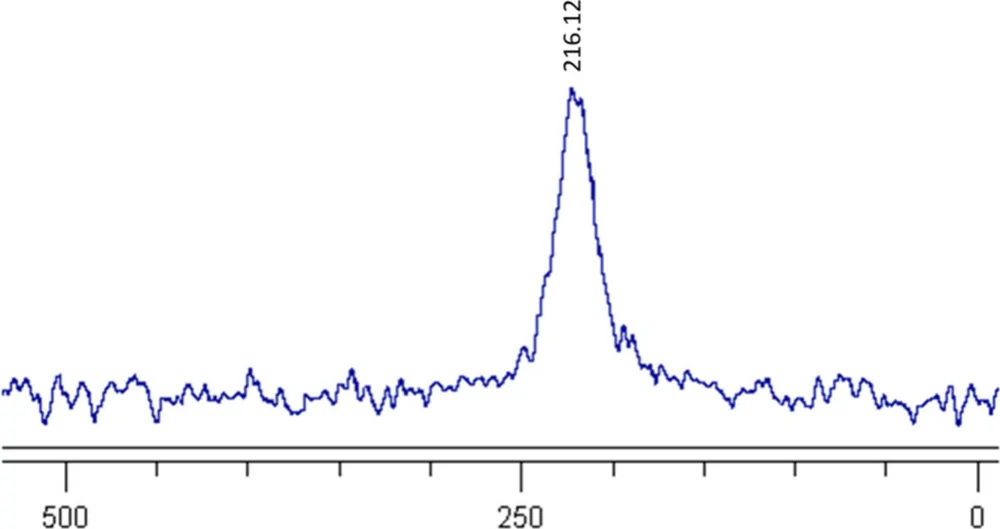
^113^Cd NMR spectrum of the [Cd­(natsc)_2_] complex.

### 3.4 Molecular Structures and Supramolecular Analysis

The molecular and crystal structures of the Hnatsc ligand (**1**) and of the complexes [ZnCl_2_(Hnatsc)_2_]·H_2_O (**2**·H_2_O) and [CdCl_2_(Hnatsc)_2_] (**6**) is discussed later using single-crystal X-ray diffraction analysis. In the following sections, an estimation is made of the coordination of the corresponding metal ions in the remaining compounds, based on analytical and spectral data.

In complexes **3** and **7**, elemental analysis indicates the presence of two ligand molecules per metal ion. The solid phase and solution have been shown to undergo ligand deprotonation, as evidenced by both IR and ^1^H NMR spectra. Furthermore, the IR spectra exhibit bands that are assignable to M-S and M-N stretching modes. Consequently, coordination around the metal ion through the thiolate sulfur and azomethine nitrogen atoms of both [natsc]^−^ should be anticipated, culminating in the formation of two κ^2^-S,N chelate rings in a distorted tetrahedral geometry (Chart 3a). A substantial number of tetrahedral structures with a [M­(κ^2^-S,N)_2_] core have been documented in the literature.^64–66^ An alternative structure could be that corresponding to a dinuclear species [M­(natsc)­(μ-S-natsc)]_2_, with two thiolate bridges between two metal ions and a κ^2^-S,N chelate on each metal [Chart 3b]. It has been demonstrated that certain zinc­(II) complexes with bis­(thiosemicarbazones) manifest this particular thiolate bridge in conjunction with the κ^2^-S,N chelates.^67,68^ However, the ^113^Cd NMR spectrum of complex **7** displays a single signal (see below), which appears to preclude the presence of any dinuclear species, at least within this complex. This observation serves to corroborate the enhanced stability exhibited by two chelates surrounding the central atom in comparison to a single chelate and an M_2_S_2_ bridge.

**III cht3:**
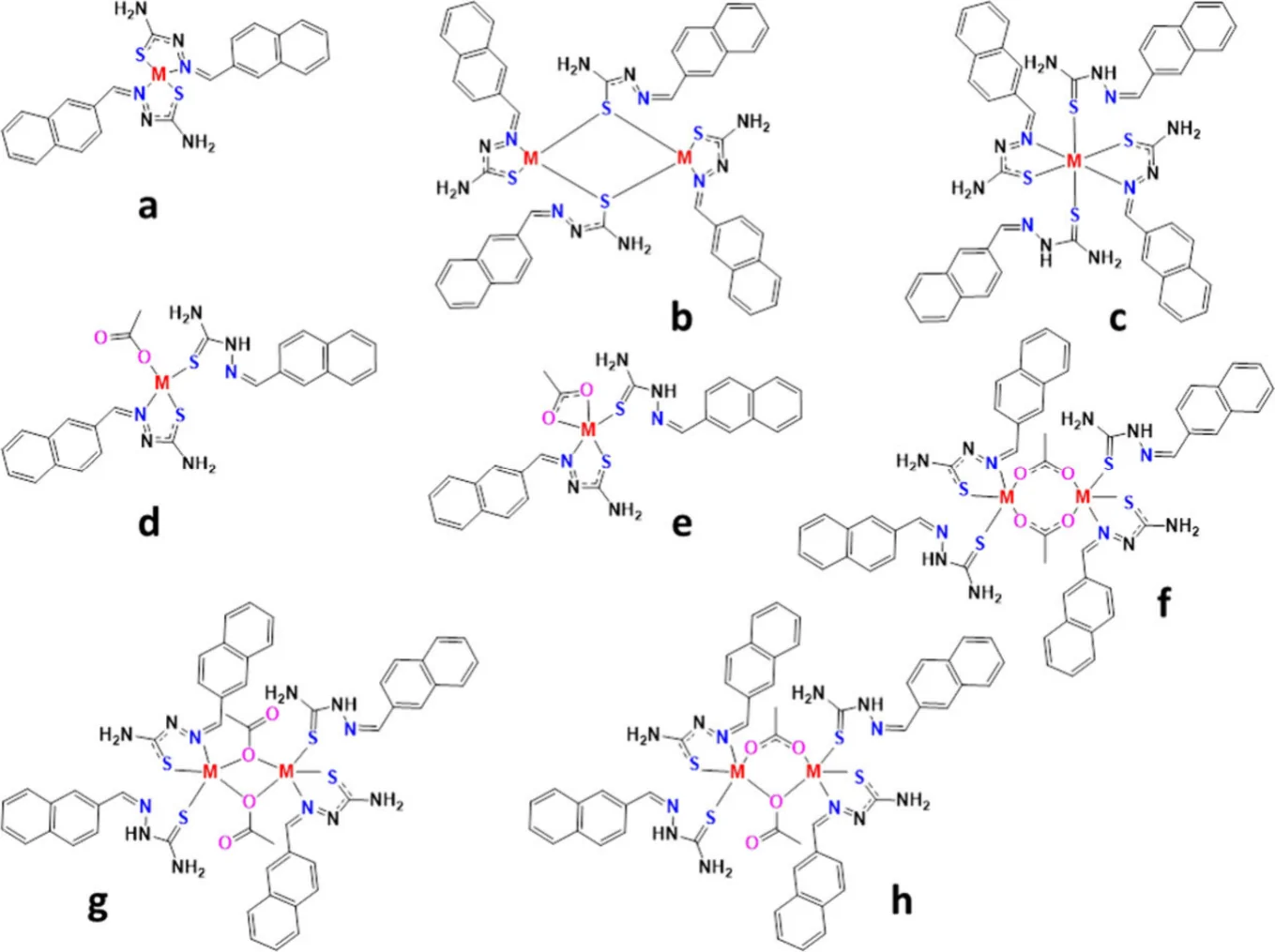
Schematic Depiction of Potential Coordination Geometry for Complexes **3**–**5**, **7**, and **8** ^a^

a Showing: a) tetrahedral *κ* ^2^-S,N chelate, b) tetrahedral dimers with thiolate bridges and *κ* ^2^-S,N chelates, c) octahedral, with *κ* ^2^-S,N chelates and thione sulfur atoms, d) tetrahedral with *κ* ^2^-S,N chelate, a thione sulfur atom, and the acetate as monodentate e) or as chelating agent, f) tetrahedral with a *κ* ^2^-S,N chelate a thione sulfur atom, and the acetates as bidentate, g) monodentate, h) or one of each.

In complex **4**, analytical data indicate the presence of the thiosemicarbazone in its neutral form, as confirmed by infrared and ^1^H NMR spectra, and in its deprotonated form, as confirmed by molecular neutrality. Consequently, the most probable coordination geometry around zinc­(II) is octahedral, with two [natsc]^−^ anions coordinated as *κ* ^2^-S,N chelates and two neutral [Hnatsc] molecules coordinated through the thione sulfur atom (Chart 3c). It is evident that several zinc­(II) complexes are recognized in which the thiosemicarbazone is coordinated in a similar manner to that observed in complex **4**.^69^

As demonstrated by the analytical and spectroscopic data, complex **5** exhibits a stoichiometry in which there is one acetate anion COO^–^, one deprotonated thiosemicarbazone, [natsc]^−^, and one neutral thiosemicarbazone [Hnatsc] for each Zn^2+^ ion. It is clear from the low molar conductivity value (see below) that the complex is neutral. This finding suggests that the complex may be a mononuclear species with a distorted tetrahedral geometry, with [natsc]^−^ coordinated in chelate mode (*κ* ^2^-S,N), Hnatsc coordinating the metal through the thione sulfur atom, and the acetate ion either as a monodentate (Chart 3d) or as a chelating agent (Chart 3e). As demonstrated in the extant literature, there are examples of zinc­(II) complexes which exhibit acetate-coordinating behavior in both cases.^51,53,70,71^ An alternative hypothesis is that the complex should be considered as dinuclear. In this case, three possible scenarios can be proposed based on the behavior of two acetate ions that act as bridges between the two zinc­(II) atoms: bidentate (Chart 3f), which is the usual one,^72–75^ monodentate (Chart 3g),^58,76,77^ or one of each (Chart 3h).^55,78,79^ In all three scenarios, the coordination number surrounding each Zn atom is five, and the most probable geometry is trigonal bipyramidal.

The stoichiometry of complex **8** is consistent with an acetate anion, COO^–^, a deprotonated thiosemicarbazone, [natsc]^−^ and a methanol molecule replaced by a cadmium­(II) cation. The same reasoning presented for complex **5** can now be applied by replacing the Hnatsc molecule coordinated to Zn^2+^ with the methanol molecule now coordinated to Cd^2+^ by the oxygen atom. The most probable structure in this case is likely to be that of a dinuclear complex with two bidentate acetate bridges (Chart 2b), similar to that of the Zn^2+^ complex (Chart 3f), with a coordination number of five around each Cd^2+^ (CdO_3_SN), and a trigonal bipyramidal geometry, which is, in principle, less distorted, by replacing the Hnatsc ligand with CH_3_OH and the Zn^2+^ with the larger Cd^2+^, thus minimizing steric hindrance.

*2-Naphthaldehyde-3-thiosemicarbazone* (Hnatsc, **1**). As can be seen in Figure 5, the asymmetric unit of this thiosemicarbazone consists of a single molecule in the *E* conformation relative to the C12–N13 carbon–nitrogen hydrazine bond. This is consistent with other uncoordinated thiosemicarbazones.^80–82^ Furthermore, the dihedral angle of 5.97(15)° between the middle planes of the naphthyl group and the thiosemicarbazone fragment confirms high planarity of the molecule. The geometric parameters listed in Table 2 indicate the presence of electronic delocalization along the thiosemicarbazone chain, which is characteristic of these compounds. However, the C–N bond involving the azomethine nitrogen atom (C11–N12) is predominantly π in character, measuring 1.279(2) Å, and is significantly shorter than the remaining N–N and C–N bonds (Table 2). These values are consistent with those found in the 4-methyl and 4-dimethyl substituted thiosemicarbazones.^81^

**Figure 5:**
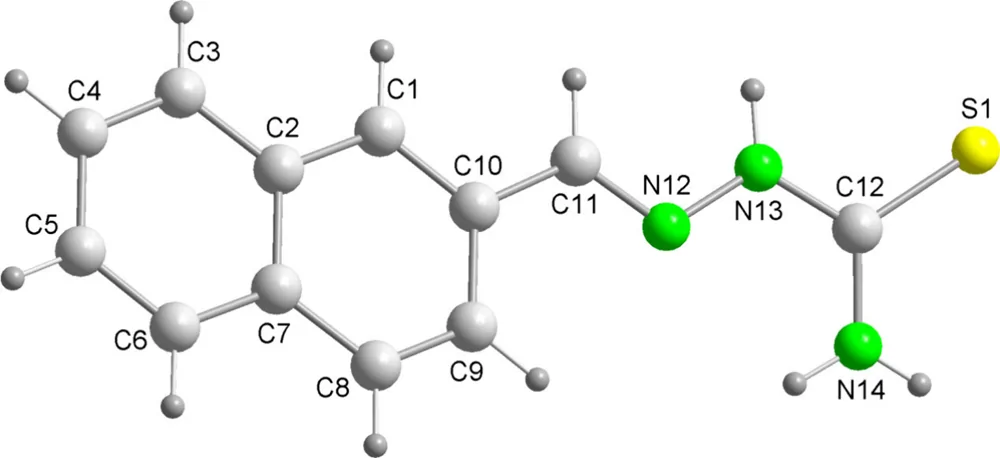
Representation of the asymmetric unit of 2-(naphthalen-2-ylmethylene)­hydrazinecarbothioamide (Hnatsc, **1**).

**Table 2:** Selection of Bond Lengths and Angles (Å and deg) for Thiosemicarbazone Moiety in the Compounds 2-(Naphthalen-2-ylmethylene)­hydrazinecarbothioamide (Hnatsc, **1**), [ZnCl_2_(Hnatsc)_2_] (**2**), and [CdCl_2_(Hnatsc)_2_]·2DMSO (**6**·2DMSO)

	Hnatsc, **1**	**2** ^a^		**6**·2DMSO
*Distances*				
C11–N12	1.279(2)	1.268(9)	1.294(9)	1.294(9)
N12–N13	1.378(2)	1.373(8)	1.375(8)	1.371(8)
N13–C12	1.346(2)	1.324(9)	1.347(9)	1.336(9)
C12–S1	1.701(2)	1.725(8)	1.699(8)	1.702(8)
C12–N14	1.320(2)	1.308(9)	1.330(9)	1.343(11)
*Angles*				
C10–C11–N12	121.73(15)	122.4(7)	120.2(7)	121.0(6)
C11–N12–N13	114.80(13)	115.4(6)	114.8(6)	114.4(6)
N12–N13–C12	120.03(13)	120.0(6)	118.7(6)	121.0(6)
N13–C12–S1	118.71(12)	117.1(6)	118.2(6)	116.6(7)
N13–C12–N14	117.91(14)	119.2(7)	117.7(7)	117.9(7)
S1–C12–N14	123.37(12)	123.7(6)	124.1(6)	125.6(6)

a Molecules I and II, respectively.

In crystal packing, each Hnatsc molecule is considered to contain three proton donors (N–H), one of which is bonded to the hydrazine nitrogen atom (N13), and two of which are bonded to the thioamide nitrogen atom (N14). The molecule also contains two potential proton acceptors: the azamethine nitrogen atom (N12) and the thiocarbonyl sulfur atom (S1). Accordingly, each molecule is connected to three nearest neighbors via N–H···S intermolecular interactions (Table 3), forming heterosynthons with the graph-set motif *R*_2_^2^(8), which are alternately fused to others with the graph-set motif *R*_4_^2^(8) (Figure 6). This gives rise to sheets that are stacked parallel to the crystallographic plane ″ab″. These sheets intersect with similar ones, but the sequence of fused heterosynthons of graph-set *R*_2_^2^(8) forms a dihedral angle of 25.03(1)° (Figure 6).

**Figure 6:**
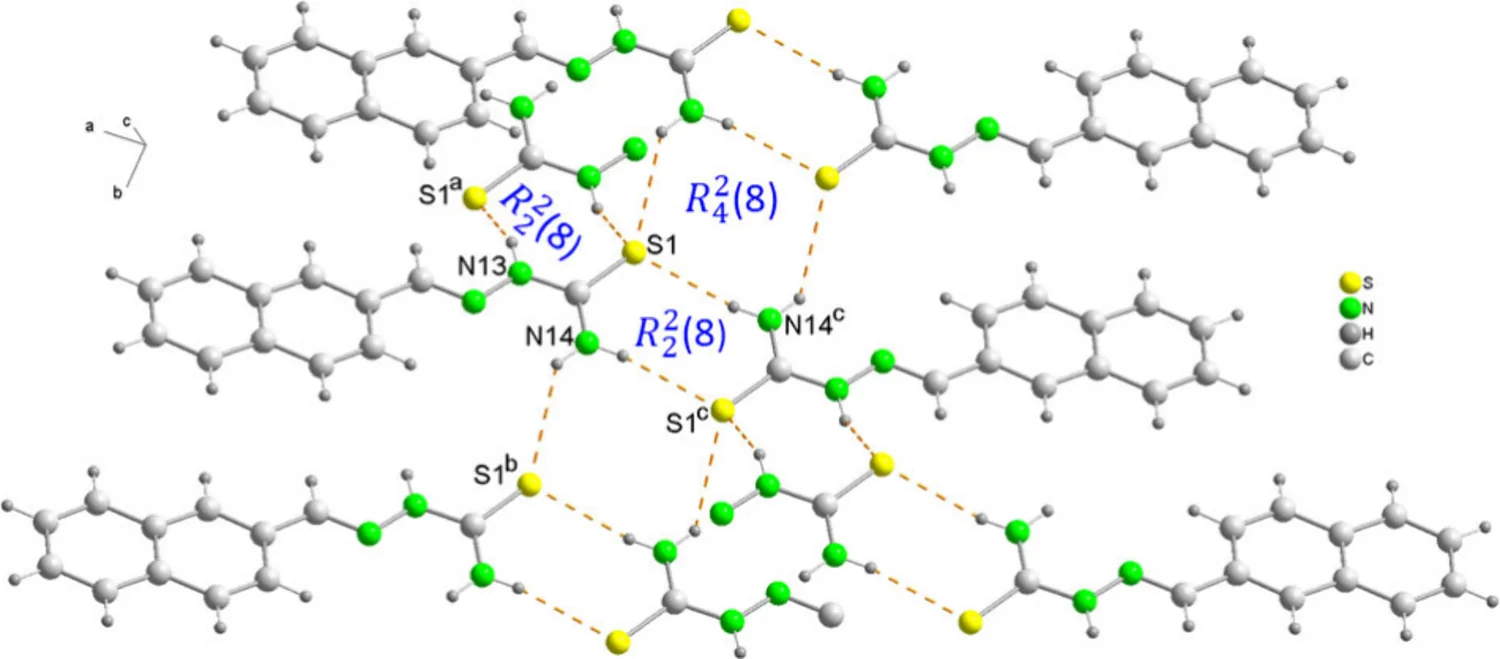
Crystal packing of the molecules in Hnatsc (**1**), including supramolecular synthons.

**Table 3:** Hydrogen Bond Parameters [Å, deg] for 2-(Naphthalen-2-ylmethylene)­hydrazinecarbothioamide (Hnatsc, **1**), [ZnCl_2_(Hnatsc)_2_] (**2**), and [CdCl_2_(Hnatsc)_2_Cl_2_]·2DMSO (**6**·2DMSO)^a^

Compound	D*–*H···A	D*–*H	H···A	D···A	∠DHA	Symmetry code
(**1**)	N13–H13A···S1^a^	0.88	2.51	3.3158(15)	151.9	–x, −y, −z
	N14–H14A···S1^b^	0.86	2.74	3.3720(15)	131.5	x, y + 1, z
	N14–H14B···S1^c^	0.84	2.51	3.3428(15)	171.2	–x – 1/2, −y + 1/2, −z
(**2**)	N13–H13A···S1^a^	0.88	2.81	3.607(7)	150.7	–x+1, −y + 1, −z
	N14–H14B···Cl2	0.88	2.65	3.465(7)	155.4	
	N23–H23A···Cl1^b^	0.88	2.58	3.396(7)	153.6	x + 1, y, z
	N23–H23A···Cl2^b^	0.88	2.97	3.512(7)	121.8	x + 1, y, z
	N24–H24A···Cl2^c^	0.88	2.71	3.499(7)	149.6	–x + 1, −y + 1, −z + 1
	N24–H24B···Cl2	0.88	2.50	3.345(7)	160.8	
(**6**·2DMSO)	N13–H13A···O2	0.88	2.03	2.790(9)	144.4	
	N14–H14A···O2^c^	0.88	2.05	2.863(8)	153.7	x, y – 1, z
	N14–H14B···Cl2	0.88	2.49	3.356(6)	168.4	

a The letters in superindex refer to the symmetry codes shown in the text and figures.

[ZnCl_2_(Hnatsc)_2_] (**2**). The asymmetric unit of compound **2** consists of a molecule of the complex formed by two thiosemicarbazone ligands acting in a neutral monodentate mode through their thione sulfur atoms and two chloride ions (Figure 7a). The coordination geometry around Zn^2+^ can therefore be described as distorted tetrahedral, with coordination sphere angles ranging from 105.45(8)° to 116.99(8)° (Table 4).

**Figure 7:**
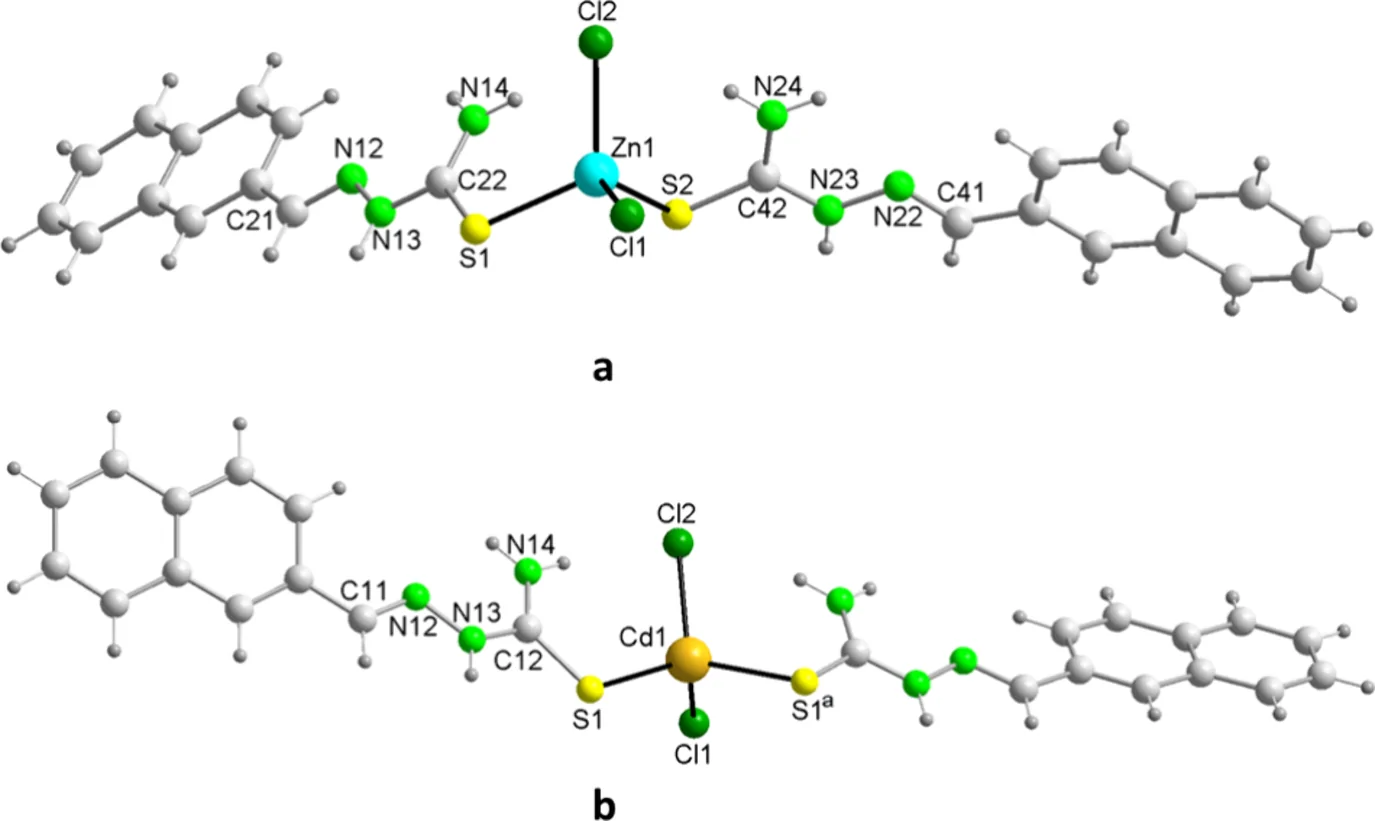
A perspective view showing the molecule and the atom-numbering scheme a) in the coordination sphere of the compound [ZnCl_2_(Hnatsc)_2_] (**2**) and b) in the asymmetric unit of [CdCl_2_(Hnatsc)_2_] (**6**).

**Table 4:** Selection of Bond Lengths and Angles (Å and deg) in the Coordination Environment of the Metal Ions of the Compounds [ZnCl_2_(Hnatsc)_2_] (**2**) and [CdCl_2_(Hnatsc)_2_]·2DMSO (**6**·2DMSO)

	**2**	**6**·2DMSO
*Distances*		
M1^a^-Cl1	2.243(2)	2.437(4)
M1-Cl2	2.287(2)	2.441(3)
M1-S1	2.340(2)	2.492(2)
M1-S2/S1^b^	2.341(2)	2.486(2)
*Angles*		
Cl1-M1-Cl2	107.58(8)	98.96(10)
Cl1-M1-S1	105.53(8)	115.84(11)
Cl2-M1-S1	113.55(8)	111.60(6)
Cl1-M1-S2/S1^b^	116.99(8)	101.71(11)
Cl2-M1-S2/S1^b^	107.93(8)	111.81(7)

a In **6**, the symmetry transformation employed to generate equivalent atoms. −x, y, −z.

b M = Zn (**2**) or Cd (**6**).

Compared to the ideal tetrahedral angle of 109.5° (Table 4), these angles provide an indication of the degree of distortion. Furthermore, the two largest angles (α and β) of the coordination neighborhood can be used to determine the geometric index parameter, *τ*_4_, which is defined as [360 – (α + β)]/141. This parameter was developed by Yang et al. for complexes with a coordination number of four, in order to quantify distortion.^83^ A value of 1 for *τ*_4_ corresponds to ideal tetrahedral geometry, whereas a value of 0 indicates perfect square-planar geometry. In complex **2**, the *τ*_4_ analysis of the distortions around the Zn­(II) ion gives a value of 0.92, indicating the aforementioned relatively low distortion. The Zn–S and Zn–Cl bond distances are very close at around 2.30 Å, although the Zn–Cl distances are somewhat shorter and the Zn–S distances are somewhat longer (Table 4). These distances are comparable with those observed in Zn complexes with related structures^84–87^ and with the average values of 2.253 Å for Zn–Cl and 2.372 Å for Zn–S found in the CSD database for Zn^2+^ complexes with the same coordination environment.^88^

In structure **2**, the two thiosemicarbazone molecules are almost perpendicular to each other, with the middle planes of the thiosemicarbazone moieties forming a dihedral angle of 82.3(3)°. However, the conformations of the two molecules differ since the rotation of the thiosemicarbazone group relative to the naphthyl ring differs. Consequently, the angle between the middle planes is 5.9(7)° in the molecule containing the S1 atom and 15.1(9)° in the molecule containing the S2 atom.

Thiosemicarbazones are known to occur in *E* and *Z* conformations, and exhibit thione-thiol tautomerism due to the presence of the thioamide functional group. In this complex, the C–S bond distances are 1.725(8) Å (S1–C22) and 1.699(8) Å (S2–C42). These values are very close to the 1.700 Å value of the uncoordinated ligand, and are intermediate between those of the single C–S bond (1.82 Å) and the double C=S bond (1.56 Å).^89^ This indicates a partial double bond character caused by delocalization. However, the C22–N44 and C42–N24 bonds are shorter than in Hnatsc. This shows that delocalization is prolonged due to coordination with Zn in the *Z* conformer mode in its thione form.

In complex **2**, both ligands have six N–H proton donor bonds, as well as sulfur and chlorine atoms that act as potential proton acceptors. These give rise to a series of hydrogen bonds in the crystalline packing. The thioamide nitrogen atoms (N14 and N24) form intramolecular hydrogen bonds with the Cl2 atom (Table 3), giving rise to two heterosynthons with the graph-set motif *R*_1_^1^(6) (Figure 8a). These heterosynthons share the Zn1–Cl_2_ bond and form a dihedral angle of 72.1(8)° between the midplanes. The other two N–H bonds on the thioamide and hydrazine nitrogen atoms (N13 and N23) form intermolecular hydrogen bonds with the S1 atom and two neighboring chlorine atoms (Table 3). This gives rise to dimers with neighboring molecules, forming homosynthons with the graph-set motif *R*_4_^2^(8) (Figure 8a). These dimers are linked to neighboring dimers through new hydrogen bonds formed between the N–H bond of the hydrazine nitrogen atom (N13) and the S1 atom of the neighboring dimer. This gives rise to new graph-set heterosynthons *R*_2_^2^(8), resulting in ribbons along the crystallographic ’c’ axis (Figure 8a). The molecular arrangement in this direction resembles a four-spoke helical chain (Figure S39). Furthermore, a new chain forms via a hydrogen bond between the N–H of the hydrazine nitrogen atom of the second ligand molecule (N23) and the Cl1 atom (Table 3), running in the direction of the crystallographic axis ″a″ (Figure 8b). Two intermolecular contacts can also be considered as π–π stacking interactions, given that the distances between the ring centroids are 3.753 Å for the C11/C12/C13/C18/C19/C20 ring and its (2 – x, 2 – y, −z)-symmetry-related form, and 3.727 Å for the C31/C32/C33/C38/C39/C40 ring and its (2 – x, −y, 1 – z)-symmetry-related form (Table 5). These data reinforce the 3D packing.^90^

**Figure 8:**
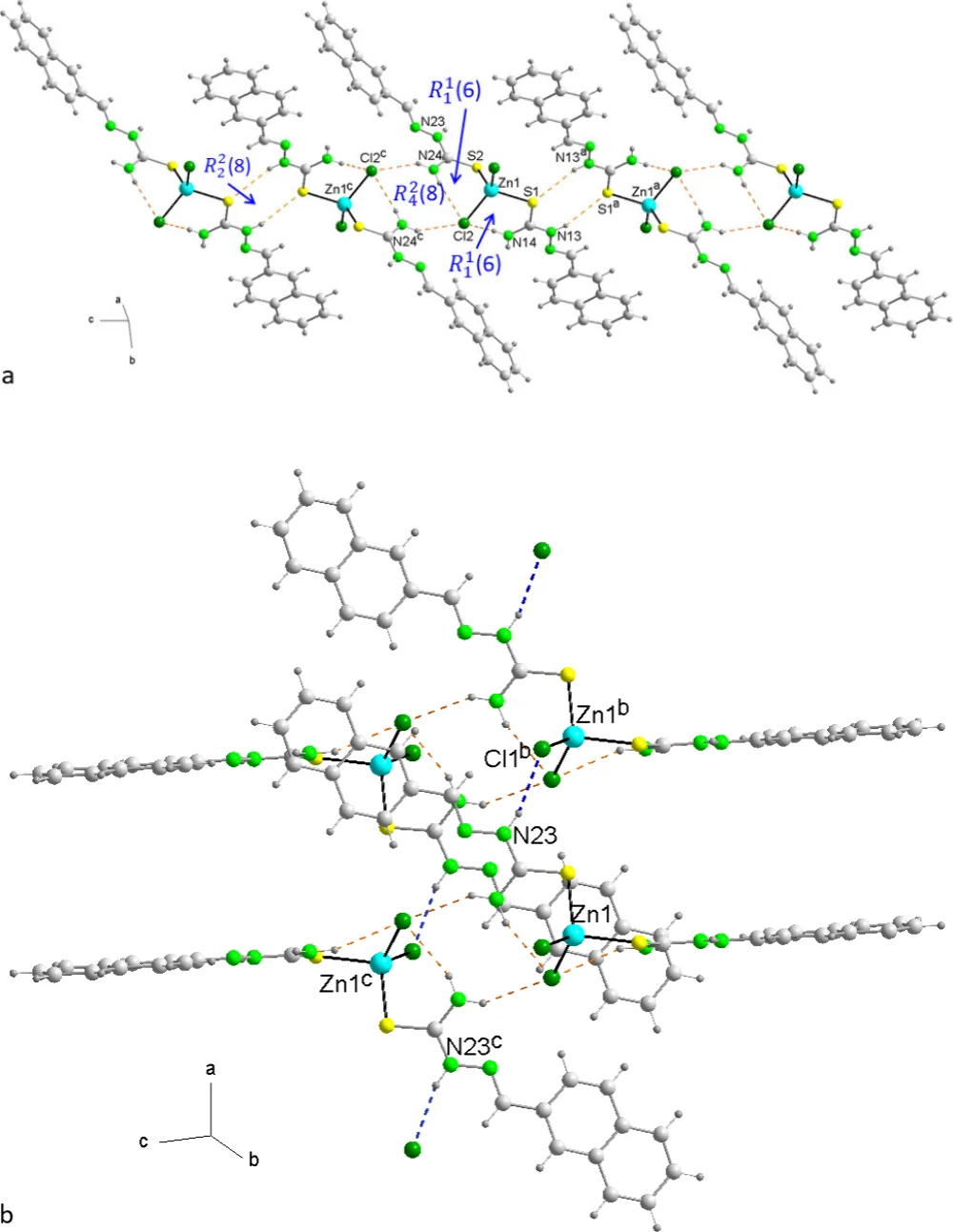
(a) A crystal packing diagram showing intermolecular interactions and supramolecular synthons for **2**. (b) A detailed view of the arrangement along the a-axis. The hydrogen bonds are shown as orange dashed lines and the N–H···Cl interactions as blue dashed lines.

**Table 5:** Intra- and Intermolecular π···π Interaction Parameters (Å, deg) for [ZnCl_2_(Hnatsc)_2_] (**2**)­^a^

π···π	Cg(I)···Cg(J)	α	CgI–Perp/CgJ–Perp	Slippage	Symmetry code
Cg(1)···Cg(1)	3.7531(9)	0	3.4833/3.4833	1.397	2 – x, 2 – y, −z
Cg(3)···Cg(3)	3.7276(9)	0	–3.6601/–3.6601	0.706	2 – x, −y, 1 – z

a Phenyl rings defined by atoms Cg(1): C11/C12/C13/C18/C19/C20; Cg(3): C31/C32/C33/C38/C39/C40. Cg­(I)···Cg­(J): centroid-centroid distance; α: Dihedral angle between planes I and J; CgI–Perp/CgJ–Perp: perpendicular distances between the planes.

[CdCl_2_(Hnatsc)_2_·2DMSO (**6**·2DMSO)]. The asymmetric unit of **6** 2DMSO consists of half a molecule of the complex and one DMSO molecule of crystallization. The molecular structure of the latter is analogous to that of the Zn complex previously described. In this case, the cadmium cation is tetrahedrally bonded to two chloride ions and to the sulfur thione atoms of a thiosemicarbazone, in a *Z* configuration, one of which is symmetrical related (Figure 7b). In the coordinated Hnatsc molecule, the middle plane of the thiosemicarbazone fragment forms an angle of 18.91(2)° with the middle plane of the naphthyl ring. It is evident that the molecular symmetry of the complex molecule under consideration results in a dihedral angle of 13.84(2)° between the middle plane of the thiosemicarbazone molecule and its symmetrical related form. These values are in stark contrast to those observed in complex **2**. Cadmium exhibits a distorted pattern with a *τ*_4_ value of 0.91, which is in close proximity to the calculated value for the Zn complex. However, the bond angles surrounding Cd^2+^ range from 98.96 to 115.84°, indicating a slightly broader interval compared to that observed around Zn^2+^. The Cd–S and Cd–Cl distances, which are very close (Table 4), are comparable with those of similar structures^14,91^ and are slightly lower than the average values of 2.622 (Cd–Cl) and 2.635 (Cd–S) found in the CSD database in Cd complexes with the same coordination environment.^88^

In the coordinated ligand, the bond lengths and angles corresponding to the thiosemicarbazone moiety are consistent with those of the uncoordinated ligand, particularly the approximate 1,700 Å C–S bond (Table 2), as observed in other analogous species.^81^ These geometric parameters are indicative of electron delocalization, as observed in the π bond, along with the bonds between C12, S1, and Cd^2+^.

The crystal packing of **6**·2DMSO is based on different types of hydrogen bonds (Table 3). Firstly, there are intramolecular hydrogen bonds between the thioamide nitrogen atom −NH_2_ and one of the chlorine atoms, Cl2. The Cl2···N14 distance is 3.356(6) Å. This gives rise to the *R*_1_^1^(6) heterosynthon, which was already seen in the Zn complex (Figure 9). Secondly, there are intermolecular hydrogen bonds between the oxygen atom of the nearest neighboring DMSO molecule and the thioamide nitrogen atom. The O2^c^···N14 distance (c = x, y – 1, z) is 2.863(8) Å. There are also hydrogen bonds between the hydrazine nitrogen atom and the O2···N13 atom. The O2···N13 distance is 2.790(9) Å. These hydrogen bonds give rise to a graph-set motif *R*_6_^3^(18). As illustrated in Figure 9, a heterosynthon is observed also to form between two molecules of the complex and two adjacent DMSO molecules in a *R*_8_^4^(20) graph-set motif. As demonstrated in Figure 10, the mononuclear units exhibit a linkage that results in the formation of a supramolecular ribbon structure extending along the crystallographic ″b″ axis. In the 3D network, the ribbons are stacked and intertwined with each other in the directions of the crystallographic axes ″a″ and ″c″ (Figure 10).

**Figure 9:**
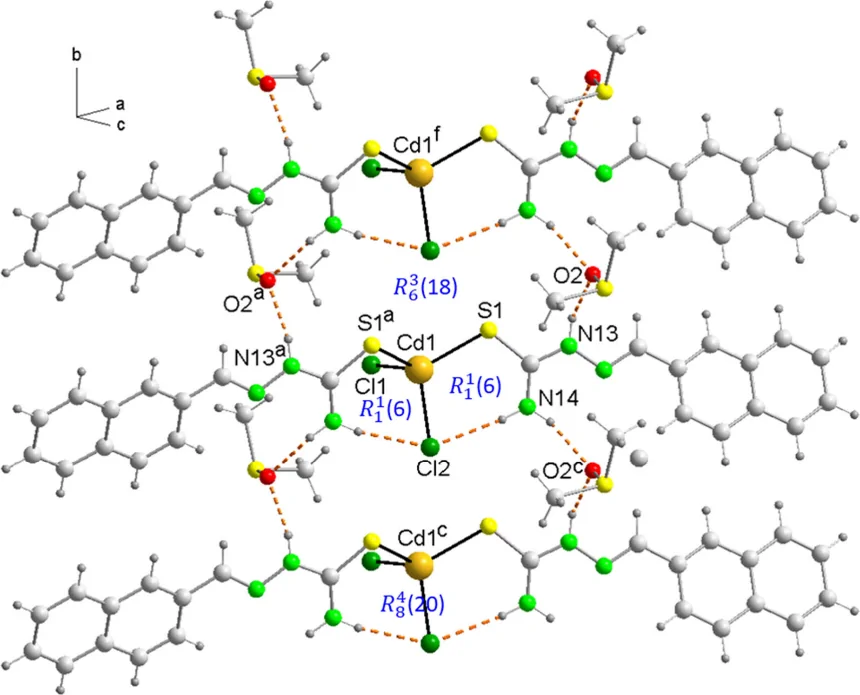
Partial packing diagram of **6**·2DMSO, showing a sequence of molecules connected by intermolecular interactions and supramolecular synthons along the ″b″ axis. Hydrogen bonds are shown as orange dashed lines.

**Figure 10:**
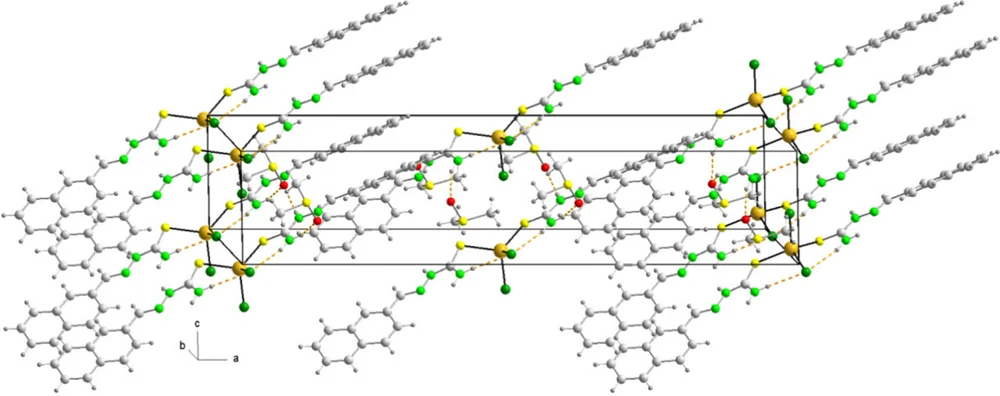
Molecular packing diagram by hydrogen bonding in **6**·2DMSO along the c-axis. Hydrogen bonds are shown as orange dashed lines.

### 3.5 DFT Study of Noncovalent Interactions in **1** and **2**

In this section, the hydrogen-bonded synthons of Hnatsc (**1**) and its zinc complex (**2**) are analyzed to investigate the strength of the hydrogen bonds and how zinc coordination affects them. This investigation is performed using density functional theory (DFT) calculations, specifically employing molecular electrostatic potential (MEP) surface analysis, quantum theory of atoms in molecules (QTAIM), and non-covalent interaction plots (NCIplot). The study focuses on motifs such as the *R*_2_^2^(8) and *R*_4_^2^(8) motifs identified in the uncoordinated ligand, as well as the *R*_4_^2^(8) and *R*_2_^2^(8) motifs observed in the zinc complex. Compound **6** was excluded from this specific comparative analysis because the presence of DMSO crystallization molecules disrupts these characteristic synthons. In compound **6**, the molecules primarily establish hydrogen bonds with the DMSO solvent rather than with each other, making it less suitable for comparing the uncoordinated ligand to the metal-coordinated form. Only those structures where single-crystal X-ray diffraction data were available (**1**, **2**, and **6**) were considered for this study.

Figure 11 illustrates the MEP surfaces of Hnatsc (**1**) and its Zn complex (**2**). In the uncoordinated ligand **1** (Figure 11a), the MEP maximum is located in the region influenced by the N13–H and C11–H groups, reaching 45.8 kcal/mol. This is followed by values of 37.0 and 38.3 kcal/mol at the terminal NH_2_ group. The MEP minimum is positioned at the S atom and exhibits an anisotropic distribution; the potential is significantly less negative along the extension of the C–S bond (−18.8 kcal/mol) than at the negative belt (−32.6 kcal/mol). The MEP over the aromatic ring is slightly negative, with values of −5.0 and −6.2 kcal/mol. In the Zn complex 2 (Figure 11b), the MEP minimum is located at the chlorido ligands (−58.2 kcal/mol), followed by the coordinated S atom (−22.0 kcal/mol). The MEP maximum is again found in the region influenced by both CH and NH groups, increasing to 55.2 kcal/mol upon coordination. An additional maximum of 40.8 kcal/mol is observed at the H atom of the terminal NH_2_ group that does not participate in the intramolecular NH···Cl H-bond. The MEP over the aromatic ring becomes almost negligible in the complex (0.0 and −1.6 kcal/mol). In summary, the coordination of the ligand to the metal center enhances its ability as an H-bond donor while decreasing its capacity as an H-bond acceptor via the S atom. Furthermore, the introduction of the chlorido ligands adds new potent H-bond acceptor sites.

**Figure 11:**
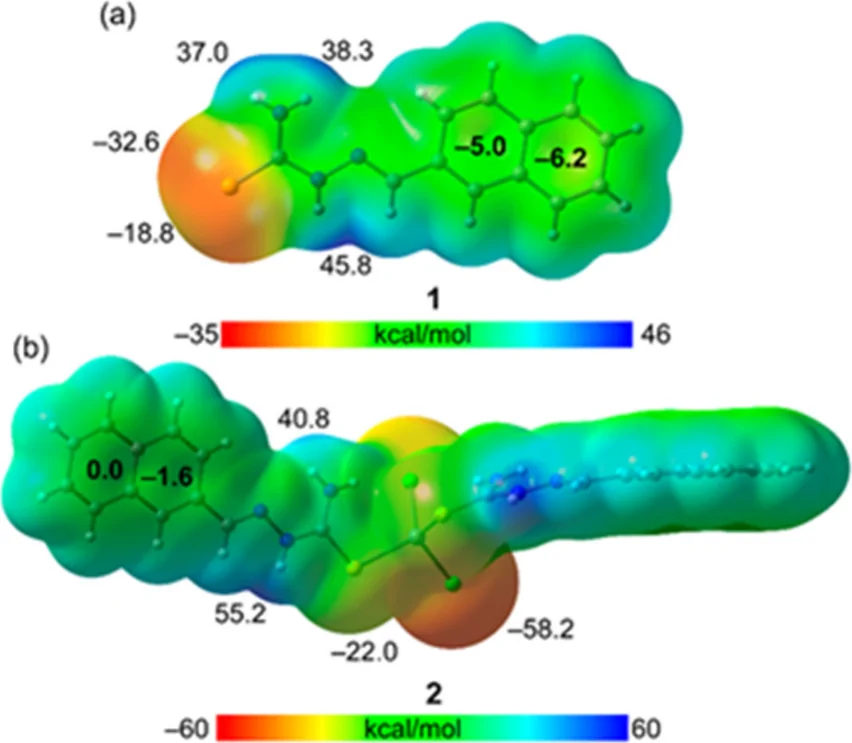
Molecular electrostatic potential (MEP) surfaces of (a) the uncoordinated ligand Hnatsc (**1**) and (b) the Zn complex [ZnCl_2_(Hnatsc)_2_] (**2**). The values at selected points (maxima and minima) are given in kcal/mol. Color ranges: −35 to 46 kcal/mol for (a) and −60 to 60 kcal/mol for (b).

The computational investigation was extended to the analysis of the characteristic hydrogen-bonded synthons observed in the solid-state architecture of **1** (as described in the previous crystallographic section and Figure 6). Figure 12 illustrates the QTAIM distribution, NCIplot surfaces, and calculated interaction energies for the three primary motifs. In the QTAIM analysis, all hydrogen bonds are characterized by corresponding bond critical points (BCPs, represented as red spheres) and bond paths (dashed lines), which are further supported by green (weak) or light blue (moderate) RDG isosurfaces.

**Figure 12:**
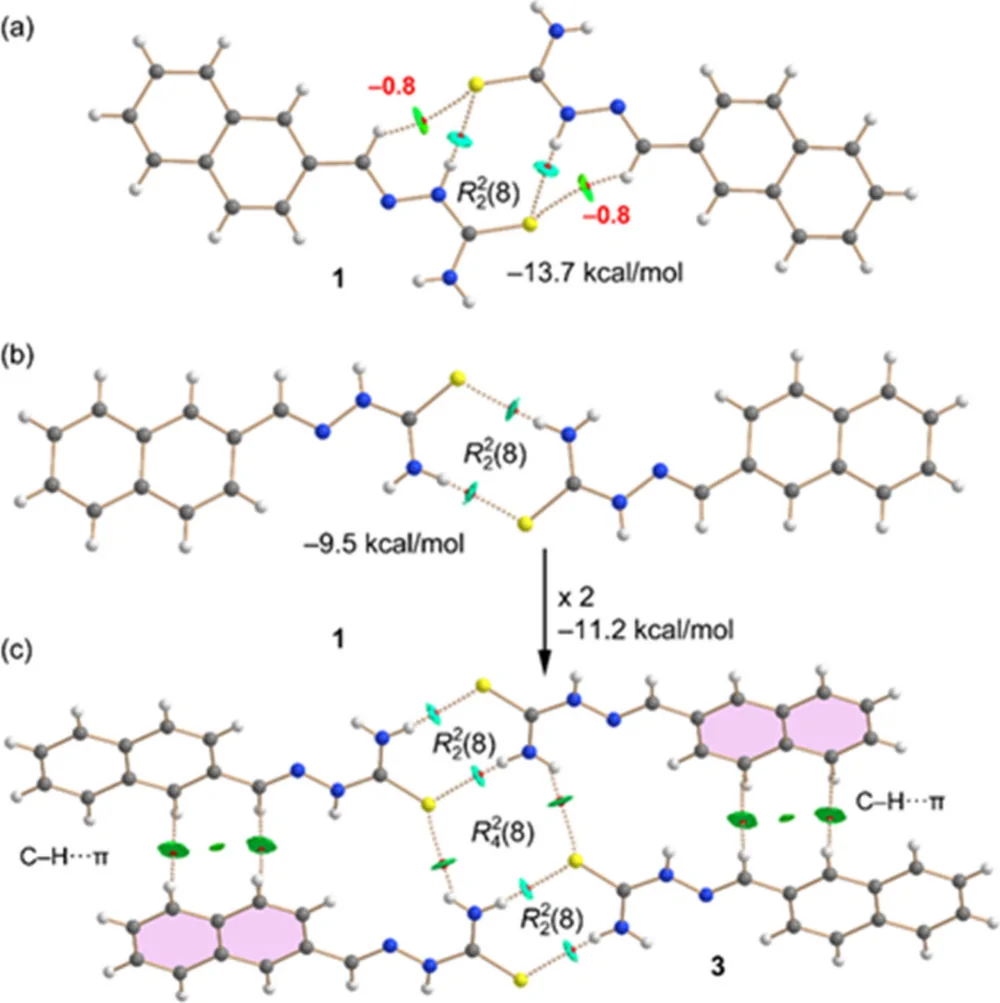
QTAIM distribution (BCPs as red spheres, bond paths as dashed lines), NCIplot (RDG = 0.45 a.u., color range −0.035 < sign­(λ_2_)­ρ < 0.035 a.u.), and dimerization energies (E_int_, kcal/mol) for the key supramolecular synthons in the crystal structure of **1**: (a) *R*_2_^2^(8) thioamide dimer, (b) *R*_2_^2^(8) terminal amine dimer, and (c) formation of the *R*_4_^2^(8) tetrameric synthon from dimer (b). The contribution of ancillary CH···S contacts is also indicated.

Figures 12a and 12b display the two distinct self-assembled dimers presenting *R*_2_^2^(8) synthons. The first dimer (Figure 12a), characterized by interactions between the central thioamide groups, exhibits a robust binding dimerization energy of -13.7 kcal/mol. The QTAIM analysis confirms the existence of symmetric NH···S hydrogen bonds, supplemented by two additional ancillary CH···S contacts, which rationalize this large dimerization energy. The contribution of each weak CH···S contact was estimated using Espinosa’s formula at −1.6 kcal/mol, confirming that the NH···S contacts are the dominant interaction. The second synthon (Figure 12b), which involves the terminal NH_2_ group, possesses a dimerization energy of −9.5 kcal/mol. This interaction is slightly weaker than that in Figure 12a, in line with the earlier MEP surface analysis which showed variations in the hydrogen donor capability across the molecule. Finally, the dimerization of the dimer shown in Figure 12b to form a tetramer (creating the *R*_4_^2^(8) synthon) was investigated (Figure 12c). The resulting dimerization energy of −11.2 kcal/mol is similar to the values computed for the *R*_2_^2^(8) dimers, suggesting comparable strengths for both the *R*_2_^2^(8) and *R*_4_^2^(8) synthons. Furthermore, the combined QTAIM/NCIplot analysis in Figure 12c reveals the presence of CH···π contacts. These are expected to be very weak, considering both the unfavorable directionality of these specific contacts and the minimal π-basicity of the aromatic rings revealed by the MEP surface analysis.

The characteristic supramolecular synthons were subsequently analyzed in the zinc complex **2** to assess the impact of metal coordination on the noncovalent interaction strengths, as illustrated in Figure 13. Regarding the self-assembled *R*_2_^2^(8) dimer shown in Figure 13a, which is equivalent to the thioamide dimer analyzed in **1**, the calculated dimerization energy is −25.4 kcal/mol. This value represents a very large stabilization, much greater than that computed for the corresponding synthon in the uncoordinated ligand (−13.7 kcal/mol). The QTAIM analysis reveals that this substantial energy increase is attributable to the modification of the ligand upon coordination. Specifically, the introduction of the chlorido ligands provides potent hydrogen bond acceptor sites (as predicted by MEP analysis), leading to the formation of two additional, symmetric CH···Cl hydrogen bonds between the chloride atoms and the aromatic protons of the naphthyl groups, which supplement the primary NH···S interactions.

**Figure 13:**
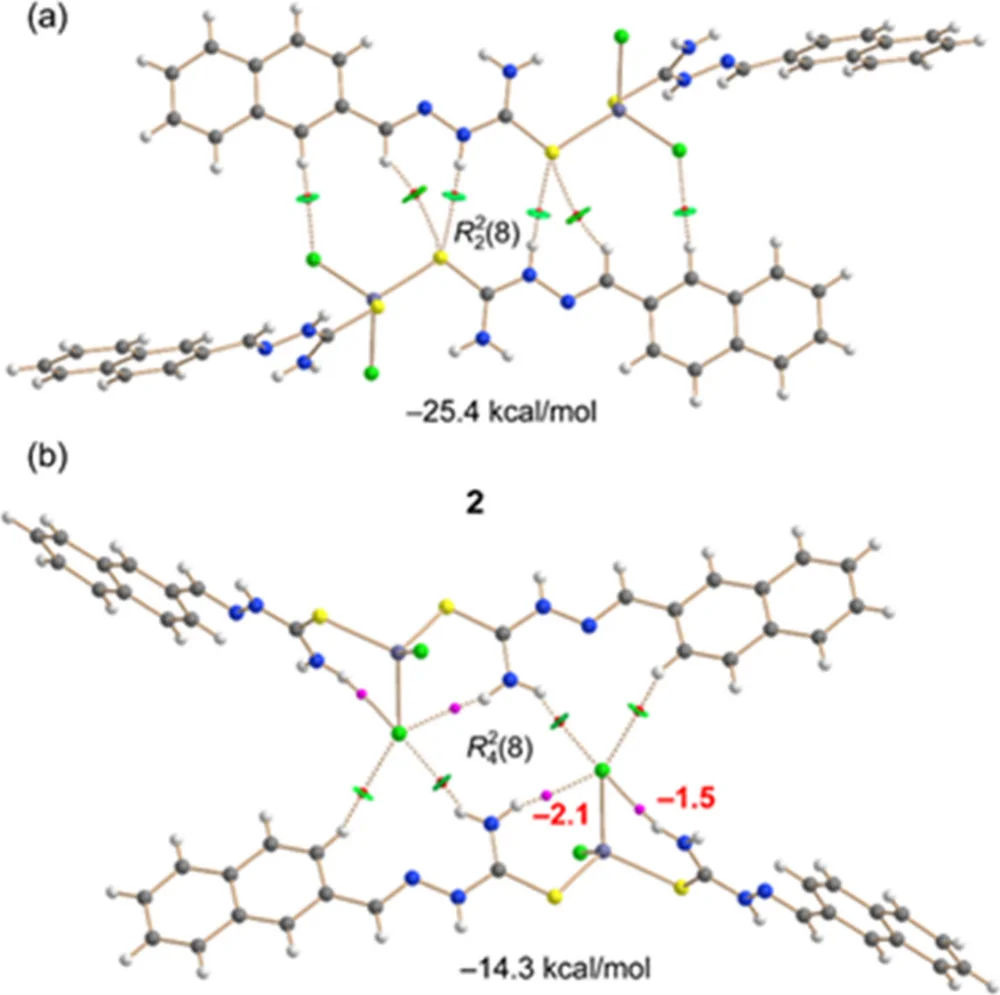
QTAIM distribution, NCIplot, and interaction energies (kcal/mol) for the key supramolecular synthons in the crystal structure of Zn complex **2**: (a) *R*_2_^2^(8) thioamide-based dimer and (b) complex and *R*_4_^2^(8) dimer featuring mixed intra- and intermolecular contacts. For (b), BCPs for intramolecular H-bonds are in pink, with individual strengths given in red text; RDG isosurfaces are omitted for intramolecular contacts. Intermolecular dimerization energies are indicated for both assemblies.

The *R*_4_^2^(8) dimer of compound **2**, shown in Figure 13b, was also examined. This complex assembly is stabilized by a combination of two intramolecular NH···Cl hydrogen bonds and two intermolecular NH···Cl contacts. In the computational visualization (QTAIM), the BCPs corresponding to the two intramolecular hydrogen bonds are highlighted using a pink color rather than the standard red to clearly distinguish them. Furthermore, the RDG isosurfaces associated with these specific intramolecular contacts have been omitted for clarity in this representation. The computed strengths of these intramolecular NH···Cl hydrogen bonds, indicated in red text adjacent to the corresponding BCPs, are −1.5 and −2.1 kcal/mol. The calculated intermolecular dimerization energy for this *R*_4_^2^(8) synthon is −14.3 kcal/mol. It is crucial to note that this stabilization energy specifically accounts for the intermolecular interactions, namely, two NH···Cl and two CH···Cl contacts, and does not include the energetic contribution of the pre-existing intramolecular contacts. Comparatively, this dimerization energy is also significantly greater than that computed for the *R*_4_^2^(8) synthon in ligand **1** (represented in Figure 12c by the tetramerization energy of −11.2 kcal/mol).

Collectively, these results reveal that the coordination of the Hnatsc ligand to the zinc­(II) metal center enhances the stability of both the characteristic *R*_2_^2^(8) and *R*_4_^2^(8) synthons. While the ligand itself becomes a better H-bond donor upon coordination, the dominant factor in the enhanced stabilization of these specific dimers is the prominent role played by the extra CH···Cl contacts formed with the newly introduced chlorido ligands.

### 3.6 Fluorescence Spectroscopy

The photophysical and fluorescent behavior study is predicated on a comparative analysis of the absorption, emission, and excitation spectra. Table 6 presents the main spectroscopic absorption and emission data for thiosemicarbazone and its complexes. Figures 14 and S40 show the electronic absorption spectra of the Hnatsc (**1**) ligand and complexes **3**, **5**–**7**, as measured from methanolic solutions (d_0_ = 1.0 × 10^–4^ mol/L for the ligand and 1.0 × 10^–5^ mol/L for the complexes). The absorption profile of all the compounds is essentially the same, showing an intense and low-lying (near UV region) absorption band. If the sum of the Gaussian-shaped bands corresponding to the different types of transition is reproduced accurately, four bands can be observed in the range 270–340 nm. The high molar absorption coefficients (log ε = 2–5) of the near-UV band indicate the presence of highly conjugated π systems. The shortest wavelength peak in each compound corresponds to the π→π* absorption of larger π-conjugated systems and the second peak is due to the n→π* absorption associated with p−π conjugation between nitrogen lone pairs and naphthalene. Above 350 nm, no additional peaks due to metal–ligand charge transfer (MLCT) absorption are observed in the complexes’ spectra.^3^

**Figure 14:**
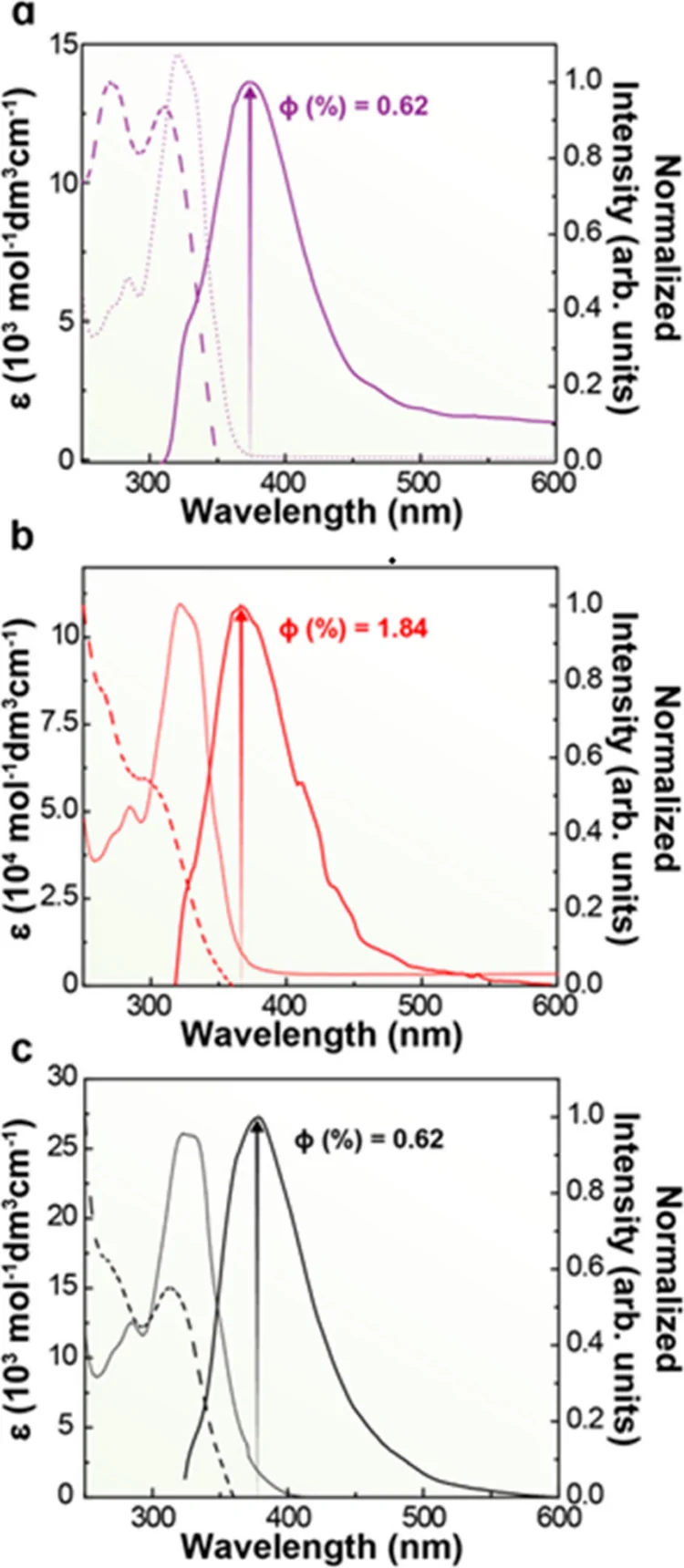
Absorption (dotted line), excitation (dashed line) and emission (solid line)­spectrum of (a) the thiosemicarbazone Hnatsc (**1**), (b) [Zn­(natsc)_2_]·3H_2_O (**3**), and (**c**) [CdCl_2_(Hnatsc)_2_] (**6**).

**Table 6:** Spectroscopic Absorption (λ_max_/nm) and Luminescence Data for the Synthesized Compounds

Compound	Absortion	Emission (λ_exc_ = 284 nm)	ϕ (%)
Hnatsc (**1**)	272, 284, 321, 329	374	0.6226
[ZnCl_2_(Hnatsc)_2_]·H_2_O (**2**·H_2_O)	272, 284, 321, 329	380	0.0849
[Zn(natsc)_2_]·3H_2_O (**3**·3H_2_O)	272, 284, 321, 332	378	1.8416
[Zn(natsc)_2_(Hnatsc)_2_]·3H_2_O (**4**·3H_2_O)	272, 284, 321, 329	378	0.3092
[Zn(OAc)(natsc)(Hnatsc)]·2H_2_O (**5**·2H_2_O)	272, 284, 321, 329	366	0.3494
[CdCl_2_(Hnatsc)_2_] (**6**)	272, 284, 322, 330	378	0.6220
[Cd(natsc)_2_] (**7**)	272, 284, 321, 329	385	0.3028
[Cd(OAc)(natsc)(MeOH)]·(**8**)	272, 284, 321, 331	380	0.1052

The emission spectra, recorded with an excitation wavelength of 284 nm, exhibit a single maximum that varies between 366 and 385 nm. In all complexes the maximum appears to be slightly shifted towards the red with respect to the ligand, such as in [Zn­(natsc)_2_]·3H_2_O, (Figure 14b), while in [CdCl_2_(Hnatsc)_2_] (Figure 14c), the maximum appears to be at a shorter wavelength.

The excitation spectra recorded at the emission maximum show at least one intense band around 380 nm. It should be noted that the excitation spectrum does not necessarily reproduce the absorption spectrum, since it is obtained by monitoring the emission at a fixed wavelength and therefore reflects only those excitation processes that contribute to the observed emission. Consequently, differences between the absorption and excitation spectra are expected, as they provide complementary information about the photophysical behavior of the compounds.^92,93^

Although the emission and excitation spectra are mirror images, the Stokes shifts, calculated between the emission and absorption maximum, which range from 3073 cm^–1^ (minimum for [Zn­(OAc)­(natsc)­(Hnatsc)]_2_·2H_2_O) to 4421 cm^–1^ (maximum for [Cd­(natsc)_2_] (Figures S40a and 40b), imply changes in the geometry of the molecule in the excited state.^36,94^ All the compounds are emissive, and in view of the spectra, the emission appears to be due to the ligand itself. However, coordination of the ligand to the metal center, except in the complex [Zn­(natsc)_2_]·3H_2_O, slightly modifies the fluorescence, as can be deduced from the ϕ values (Table 6). In fact, in most complexes, coordination produces a quenching effect, as observed in the lower quantum yield of the complexes with respect to the ligand, except in [CdCl_2_(Hnatsc)_2_] (**6)** (Figure 14c) where the ϕ value is equal to that obtained for uncoordinated thiosemicarbazone. However, a CHEF effect is observed in [Zn­(natsc)_2_]·3H_2_O, since this complex exhibits a quantum yield that is practically three times that of the ligand (Figure 14b). This is quite common, as in many cases, the fluorescence of organic ligands is increased by coordination to the metal center due to the formation of chelate rings and, therefore, an increase in the system’s rigidity. Furthermore, by comparing the ϕ values of structurally similar zinc­(II) and cadmium­(II) complexes, we can deduce that Zn^2+^ is more effective than Cd^2+^ in the ligand.

To gain deeper insight into the absorption and fluorescence properties of the compounds, TD-DFT calculations were performed at the CAM-B3LYP/def2-TZVP level of theory. The theoretical absorption and emission spectra (Figure 15) show good agreement with the experimental profiles, correctly identifying the main transitions in the near-UV region. For the uncoordinated ligand **1**, the primary excitation (S_0_ → S_2_) at 285.4 nm is dominated by a HOMO to LUMO transition (91%), see Table 7. In the metal complexes, coordination leads to a mixing of states; for instance, the S_0_ → S_1_ transition in complex **2** (287.1 nm) and complex **6** (288.1 nm) involves significant contributions from both HOMO to LUMO and HOMO–1 to LUMO+1 configurations.

**Figure 15:**
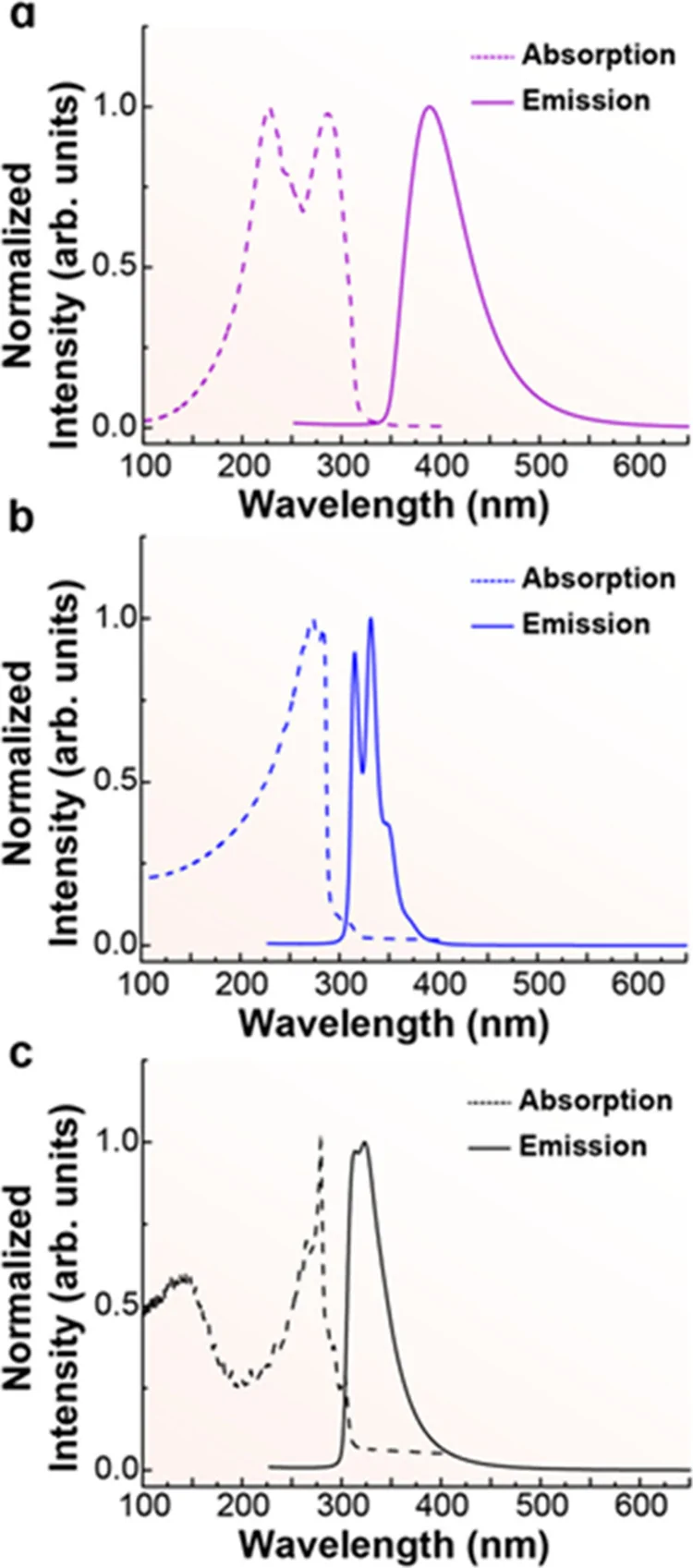
Normalized theoretical absorption and fluorescence emission spectra for a) **1**, b) **2**, and c) **6** calculated at the CAM-B3LYP/def2-TZVP level of theory.

**Table 7:** Computed Excitation Wavelengths (λ, nm), Oscillator Strengths (f_osc_), and Electronic Compositions for the Main Electronic Transitions of **1**, **2**, and **6** at the CAM-B3LYP/def2-TZVP Level of Theory

Excitation	λ	f_osc_	Composition
Compound **1**			
S_0_ → S_2_	285.4 nm	1.2017	HOMO → LUMO (91%)
S_0_ → S_5_	231.5 nm	0.4197	HOMO → LUMO (65%)
			HOMO–1 → LUMO+1 (33%)
Compound **2**			
S_0_ → S_1_	287.1	1.4313	HOMO → LUMO (54%)
			HOMO–1 → LUMO+1 (28%)
S_0_ → S_3_	280.0	1.0900	HOMO → LUMO (46%)
			HOMO–1 → LUMO+1 (30%)
Compound **6**			
S_0_ → S_1_	288.1	1.9350	HOMO → LUMO (43 %)
			HOMO–1 → LUMO+1 (40 %)

### 3.7 Theoretical Photophysical Study

The molecular orbital analysis (Figure 16) reveals that the HOMO and HOMO–1, as well as the LUMO and LUMO+1, are nearly degenerate in complexes **2** and **6**, in line with the mixing composition of the excitations. These orbitals are primarily localized on the π-system of the thiosemicarbazone ligand, confirming that the electronic transitions are ligand-centered (LC) in nature, with minimal metal participation. This is further supported by the electron density difference maps (EDDM) for the S_1_ → S_0_ fluorescence band (Figure 17), which illustrate the charge redistribution occurring upon relaxation to the ground state.

**Figure 16:**
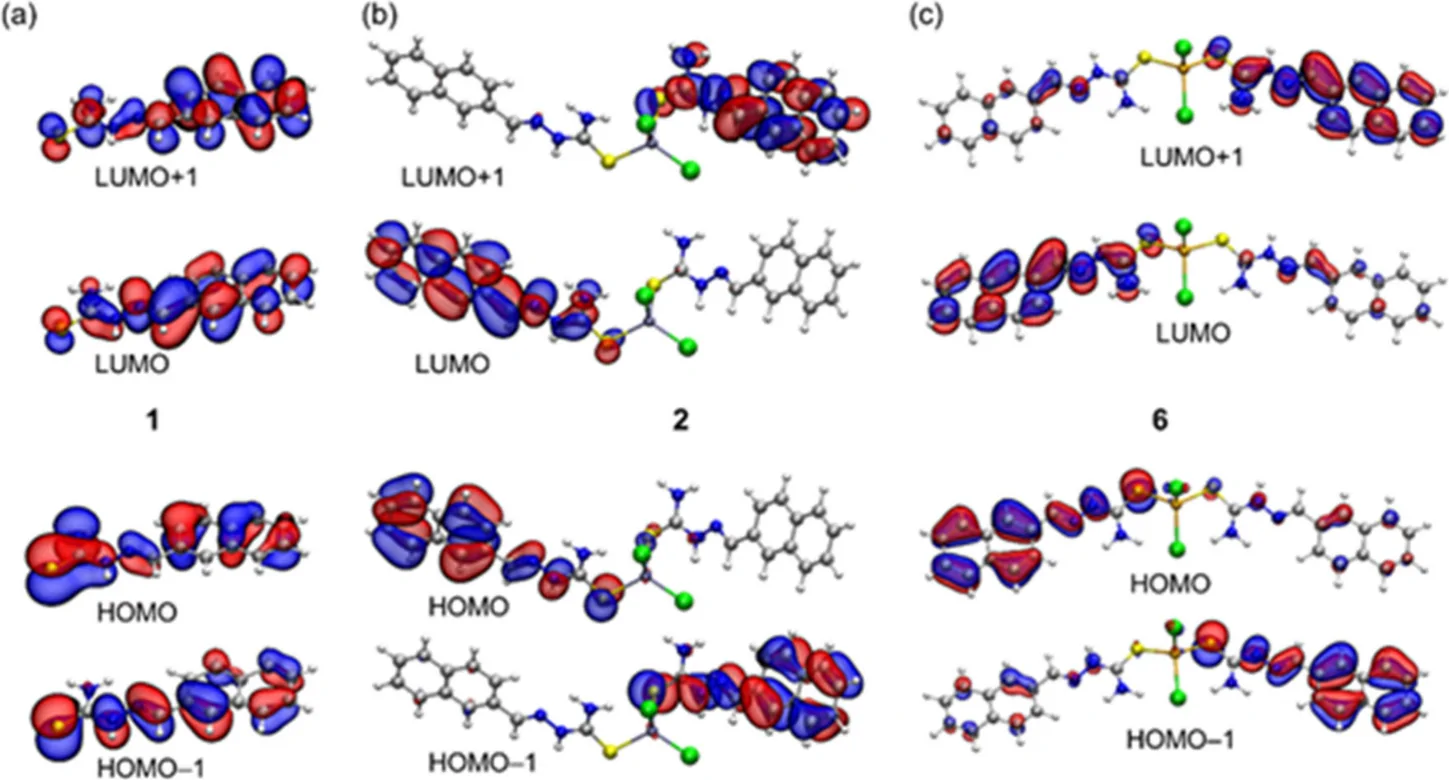
Isosurface plots of the nearly degenerate frontier molecular orbitals (HOMO–1 to LUMO+1) for compounds **1** (a), **2** (b), and **6** (c).

**Figure 17:**
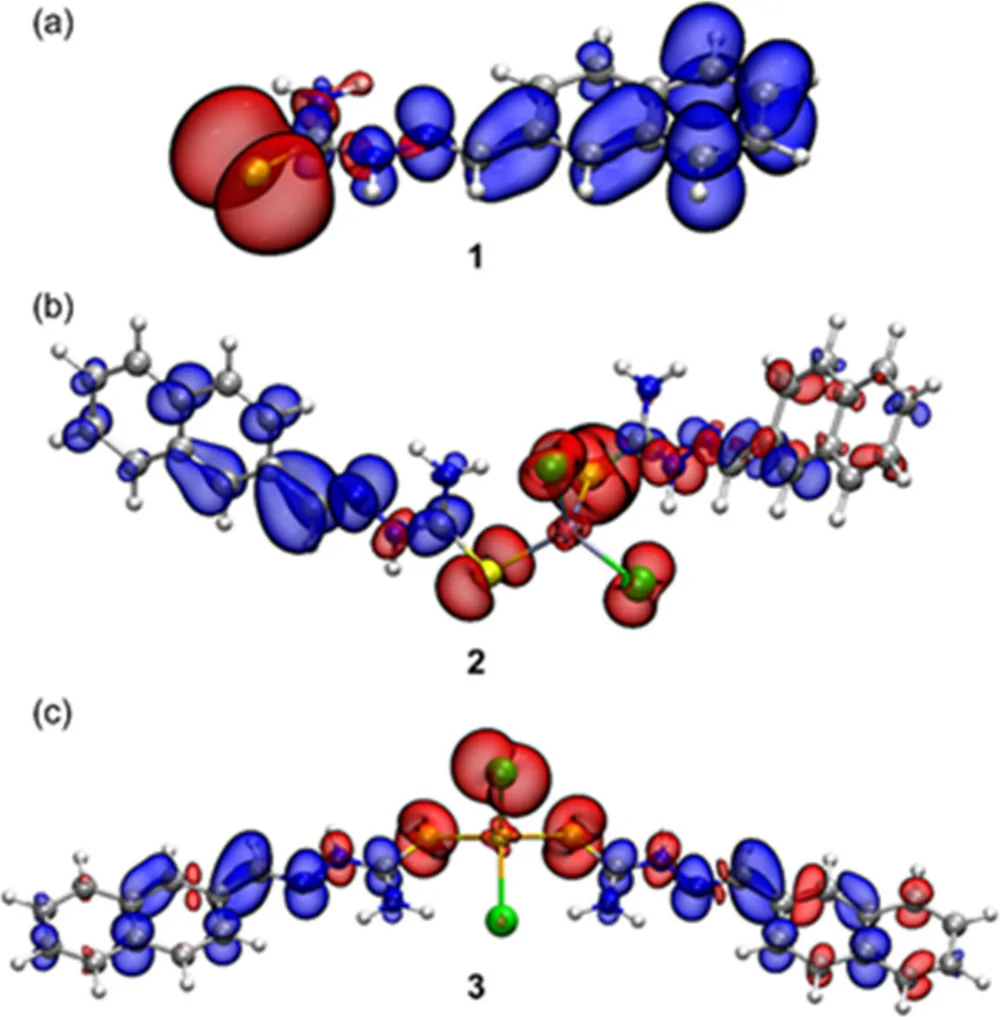
Electron density difference maps (EDDM) for **1** (a), **2** (b), and **6** (c) corresponding to the S_1_ → S_0_ fluorescence transition. Blue and red regions indicate areas of electron density increase and decrease, respectively.

The calculated fluorescence parameters (Table 8) successfully reproduce the experimental quantum yield tendencies. The theoretical quantum yields (φ) were determined to be 1.25% for **1**, 0.86% for **2**, and 2.41% for **6**. The decrease in quantum yield for the zinc complex **2** compared to the uncoordinated ligand is associated with a high internal conversion rate (k_IC_ = 1.42 × 10^11^ s^–1^), which outweighs the radiative rate (*k*_f_). In contrast, complex **6** exhibits the highest efficiency among the three, consistent with the experimental observation that cadmium coordination can sustain or enhance the fluorescent response in these thiosemicarbazone derivatives.

**Table 8:** Calculated Radiative (*k*_f_) and Non-Radiative (k_IC_) Rate Constants, and Theoretical Fluorescence Quantum Yields (ϕ) for **1**, **2**, and **6** Determined between the S_0_ and S_1_ States at the CAM-B3LYP/def2-TZVP Level of Theory

	*k*_f_ (s^–1^)	k_IC_ (s^–1^)	φ (%)
Ligand Hnatsc, **1**	2.71·10^7^	2.15·10^9^	1.25
[ZnCl_2_(Hnatsc)_2_]·H_2_O, (**2**·H_2_O)	1.22·10^9^	1.42·10^11^	0.86
[CdCl_2_(Hnatsc)_2_], **6**	1.09·10^9^	4.41·10^10^	2.41

## 4 Conclusions

In the present study, in addition to the previously structurally uncharacterized 2-thiophenaldehyde-N(4)-naphthylthiosemicarbazone, four zinc­(II) and three cadmium­(II) complexes with this thiosemicarbazone were synthesized in the presence of MCl_2_ or M­(OAc)_2_ (M = Zn, Cd). Spectroscopic characterization of all compounds was also conducted. Furthermore, the thiosemicarbazone and the ZnCl_2_ and CdCl_2_ complexes were subjected to X-ray diffraction analysis to facilitate their structural elucidation. As demonstrated by the spectroscopic and structural studies presented here, two different coordination modes (monodentate and bidentate) were identified in the thiosemicarbazone. This study has demonstrated the capacity of HNTSC to generate mononuclear and dinuclear species with these coordination modes. The examples presented in this study demonstrate two coordination modes: The compound is classified as S-monodentate in its neutral form and N,S-bidentate in its monoanionic form. The latter forms a five-membered chelate ring with the metal ion. Furthermore, the coordination mode appears to be contingent on the basicity of the reaction medium, the presence of acetate as an additional ligand, the ligand/metal ratio, and the identity of the metal salt. Moreover, an analysis of the intermolecular interactions in the three molecules was conducted. Crystal structures of complexes [ZnCl_2_(Hnatsc)_2_] and [CdCl_2_(Hnatsc)_2_]·2DMSO exhibit N–H···Cl hydrogen bonds. DFT analysis further quantified these interactions, demonstrating that the coordination to Zn­(II) enhances the stability of characteristic *R*_2_^2^(8) and *R*_4_^2^(8) synthons. Structural analysis using X-ray diffraction has revealed the presence of both classical and non-classical intramolecular and intermolecular interactions. These interactions are the primary contributors to the stabilization of the crystal structures of these compounds. All synthesized compounds exhibit a fluorescent response in methanol solution, displaying a Stokes shift. Furthermore, the complexes demonstrate systems with enhanced π conjugation and electron delocalization in comparison to uncoordinated thiosemicarbazone. The present study establishes the foundation for the development of efficient luminescent sensors for environmental and safety purposes, while also exploring new avenues for the research of zinc­(II) and cadmium­(II) complexes in materials science. Subsequent research will concentrate on enhancing the sensitivity and selectivity of these complexes for a broader range of substances and on investigating their applicability in real-world scenarios. TD-DFT calculations confirmed that these transitions are ligand-centered, involving nearly degenerate frontier orbitals in the metal complexes. The theoretical study successfully rationalized the experimental quantum yields, showing that the fluorescence quenching in the zinc complex **2** results from an exceptionally high internal conversion rate, whereas the cadmium complex **6** maintains high efficiency due to a lower non-radiative deactivation rate.

## Supplementary Material



## Notes

ESI–MS, FT-IR spectra, ^1^H, ^13^C, and ^113^Cd NMR spectra (PDF)
