# Supplementary material for: Structural Variety, Luminescent Properties, and DFT Calculations of 2‑Naphthaldehyde Thiosemicarbazone and Its Complexes with Zinc(II) and Cadmium(II)
Source: Inorg Chem. 2026 Sep 15;65(38):22343–61. doi: 10.1021/acs.inorgchem.6c02677 (PMC13625970; doi:10.1021/acs.inorgchem.6c02677)
Supplement: Supplementary file 1 [file ic6c02677_si_001.pdf]

Supporting Information

Structural variety, luminescent properties and DFT calculations of 2-naphthaldehyde thiosemicarbazone and its complexes with zinc(II) and cadmium(II)

Alfonso Castiñeiras,[1] Isabel García-Santos,[1] Lorenzo Seijo,[1] Julio Corredoira-Vázquez,[2,3] Sergi Burguera,[4] Antonio Frontera[4]**

[1]Department of Inorganic Chemistry, Faculty of Pharmacy, University of Santiago de Compostela, 15782 Santiago de Compostela, Spain

[2]Departamento de Química Inorgánica, Facultade de Química, Universidade de Santiago de Compostela, 15782 Santiago de Compostela, Spain.

[3]Institute of Materials (iMATUS), Universidade de Santiago de Compostela, 15782 Santiago de Compostela, Spain

[4]Departament de Química, Universitat de les Illes Balears, Crta e Valldemossa km 7.5, 07122 Palma de Mallorca (Baleares), Spain

*email address: *Alfonso Castiñeiras* (alfonso.castineiras@usc.es); *Antonio Frontera* (toni.frontera@uib.es)

Index

EI-MS spectra ……………………………………………3

Figures S1 to S5

FT-IR Spectra…………………………………………….9

Figures S6 to S21

^{1}H NMR Spectra……………………………………26

Figures S22 to S31

^{13}C{^{1}H] NMR Spectra………………………………38

Figures S32 to S38

Crystallography……………………………………46

Figure S39

Fluorescence Spectra………………………………48

Figure S40

EI-MS spectra

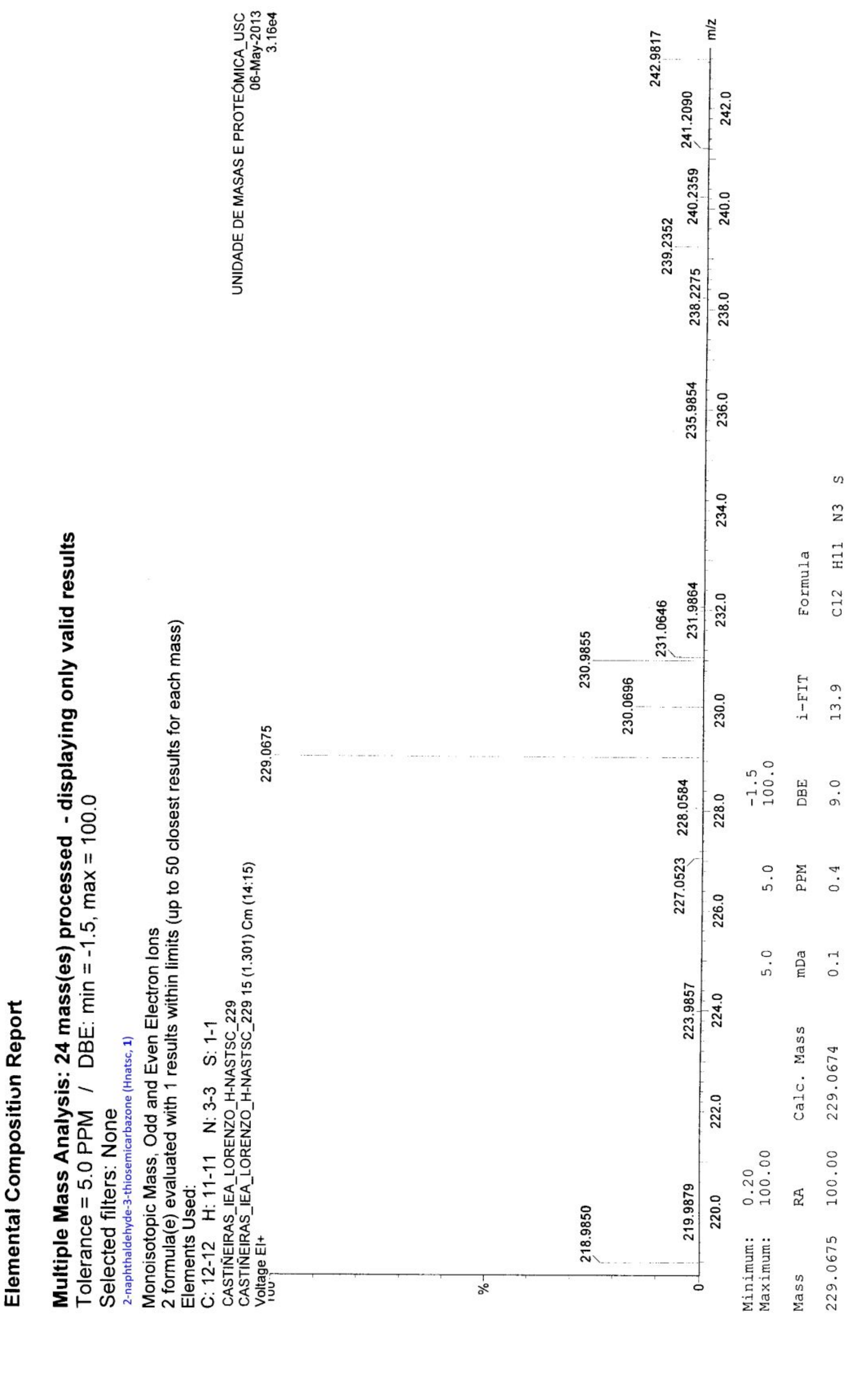

Figure S1: The EI-MS spectrum of the compound **1**

USC
UNIVERSIDADE DE SANTIAGO DE COMPOSTELA

RIAIDT
UNIDADE DE ESPECTROMETRIA DE MASAS E PROTEÓMICA
Edificio CACTUS * Campus Vida
15782 Santiago de Compostela (Spain)
Tel. 981 563 100. Ext. 16242 * Fax 981 528098
E-mail: sxemasas@usc.es
Web: http://www.ti.usc.es/riaidt/masas/masas.htm

ESPECTRO ESI-FIA-TOF

Analysis Info

Sample Name	ZN11_611_1-D,9_01_9990.d [Zn(Hnatsc)$_2$Cl$_2$] (**2**)	Acquisition Date	6/4/2013 8:09:55 PM
Method	fia_pos_medio_350-1500_egf.m	Instrument	micrOTOF

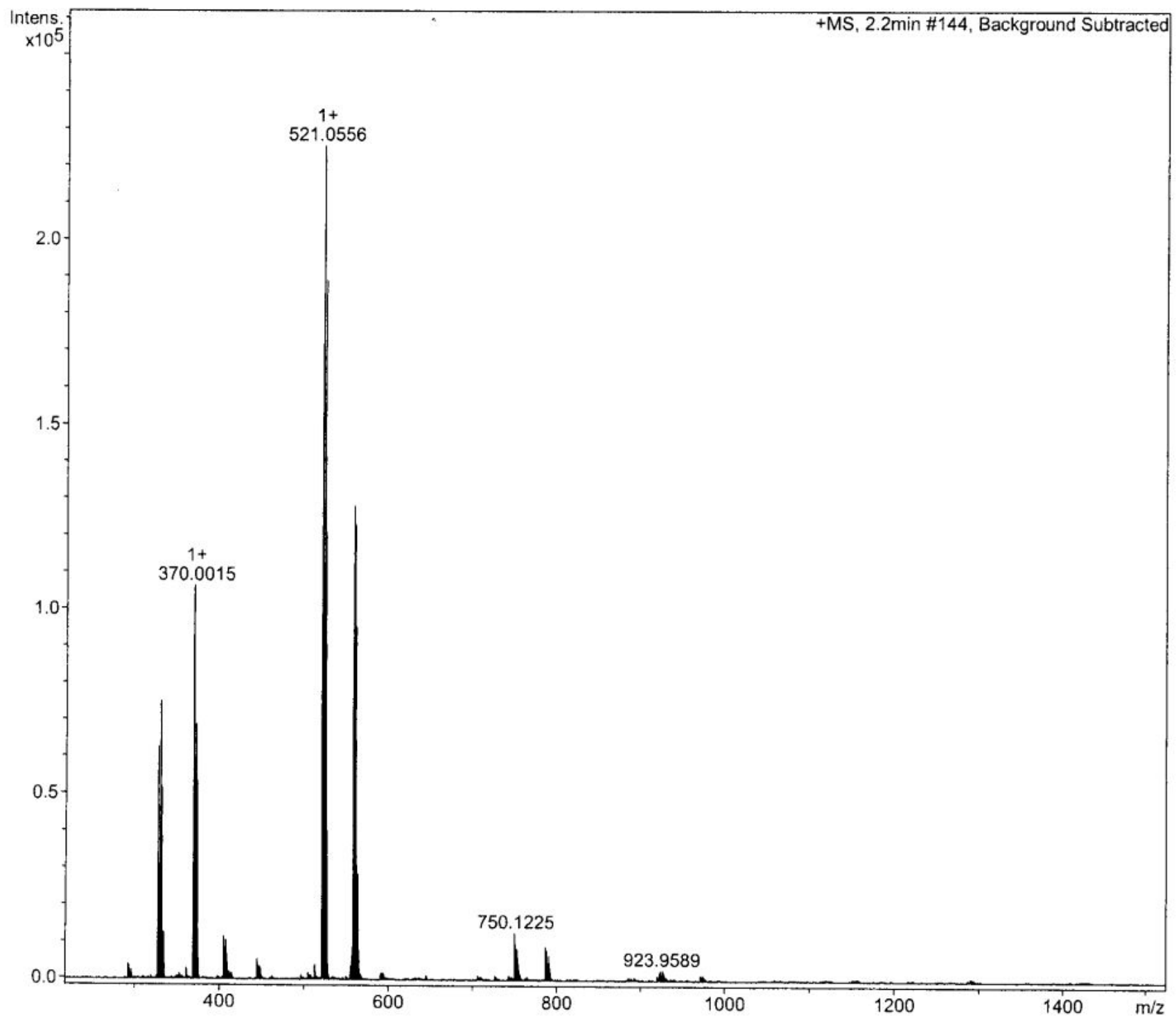

Bruker Compass DataAnalysis 4.0

Figure S2: The EI-MS spectrum of the compound **2**

⑦

USC UNIVERSIDADE DE SANTIAGO DE COMPOSTELA

RIAIDT
UNIDADE DE ESPECTROMETRIA DE MASAS E PROTEÓMICA
Edificio CACTUS * Campus Vida
15782 Santiago de Compostela (Spain)
Tel. 981 563 100. Ext. 16242 * Fax 981 528098
E-mail: sxemasas@usc.es
Web: http://www.ti.usc.es/riaidt/masas/masas.htm

ESPECTRO ESI-FIA-TOF

Analysis Info

Sample Name	ZN11B_---_1-D,12_01_10583.d [Zn(natsc)$_2$(Hnatsc)$_2$]·3H$_2$O (**4**·3H$_2$O)	Acquisition Date	7/22/2013 1:09:24 PM
Method	fia_pos_medio_350-1500_egf.m	Instrument	micrOTOF

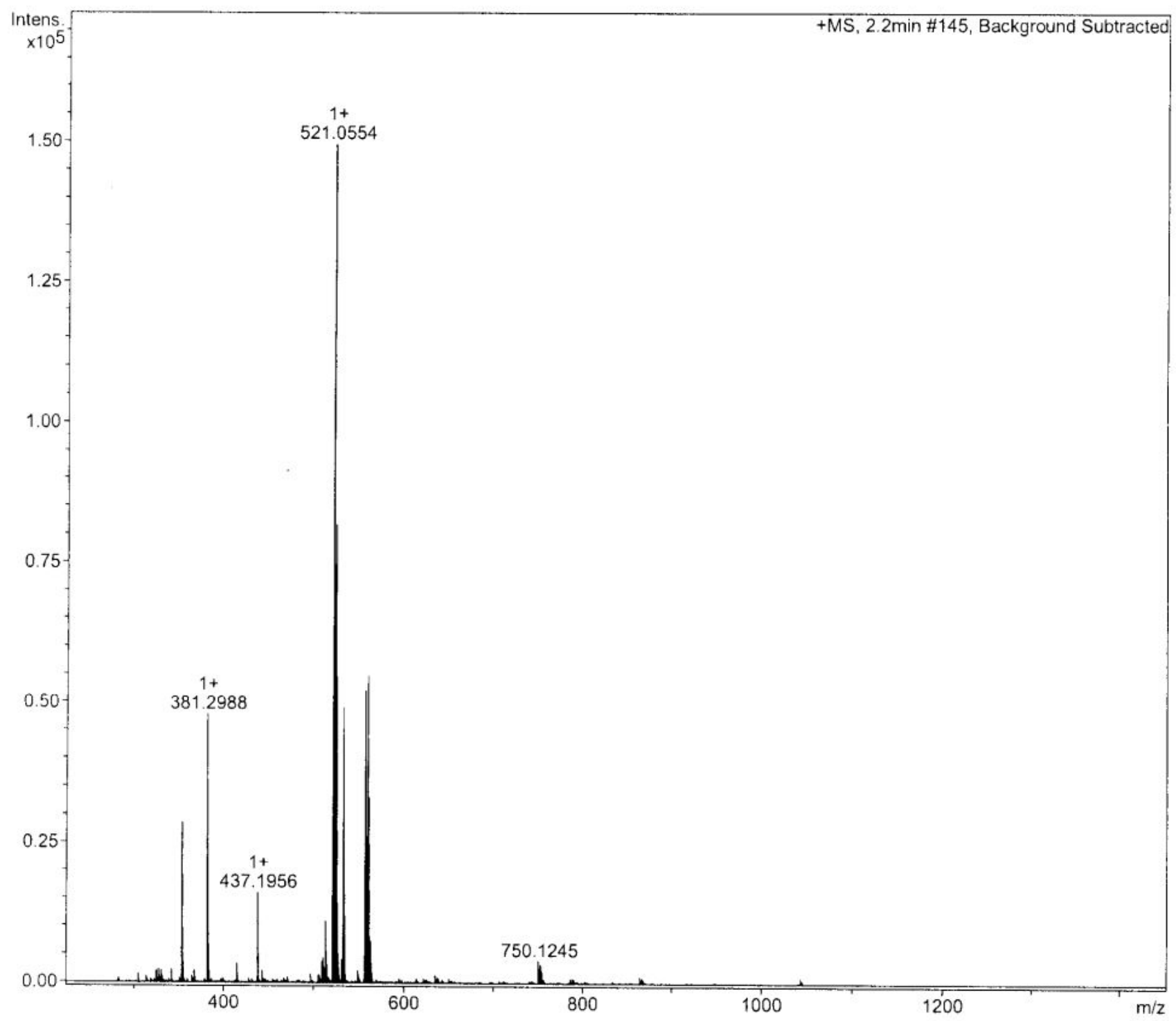

Bruker Compass DataAnalysis 4.0

Figure S3: The EI-MS spectrum of the compound **4**

RIAIDT
UNIDADE DE ESPECTROMETRIA DE MASAS E PROTEÓMICA
Edificio CACTUS * Campus Vida
15782 Santiago de Compostela (Spain)
Tel. 981 563 100. Ext. 16242 * Fax 981 528098
E-mail: sxemasas@usc.es
Web: http://www.ti.usc.es/riaidt/masas/masas.htm

ESPECTRO ESI-FIA-TOF

Analysis Info

Sample Name	ZNAC12_640_1-F,4_01_10206.d	Acquisition Date	6/20/2013 7:24:21 PM
Method	fia_pos_medio_350-1500_egf.m	Instrument	micrOTOF
	[Zn(OAc)(natsc)(Hnatsc)]·2H_2O (**5**·2H_2O)		

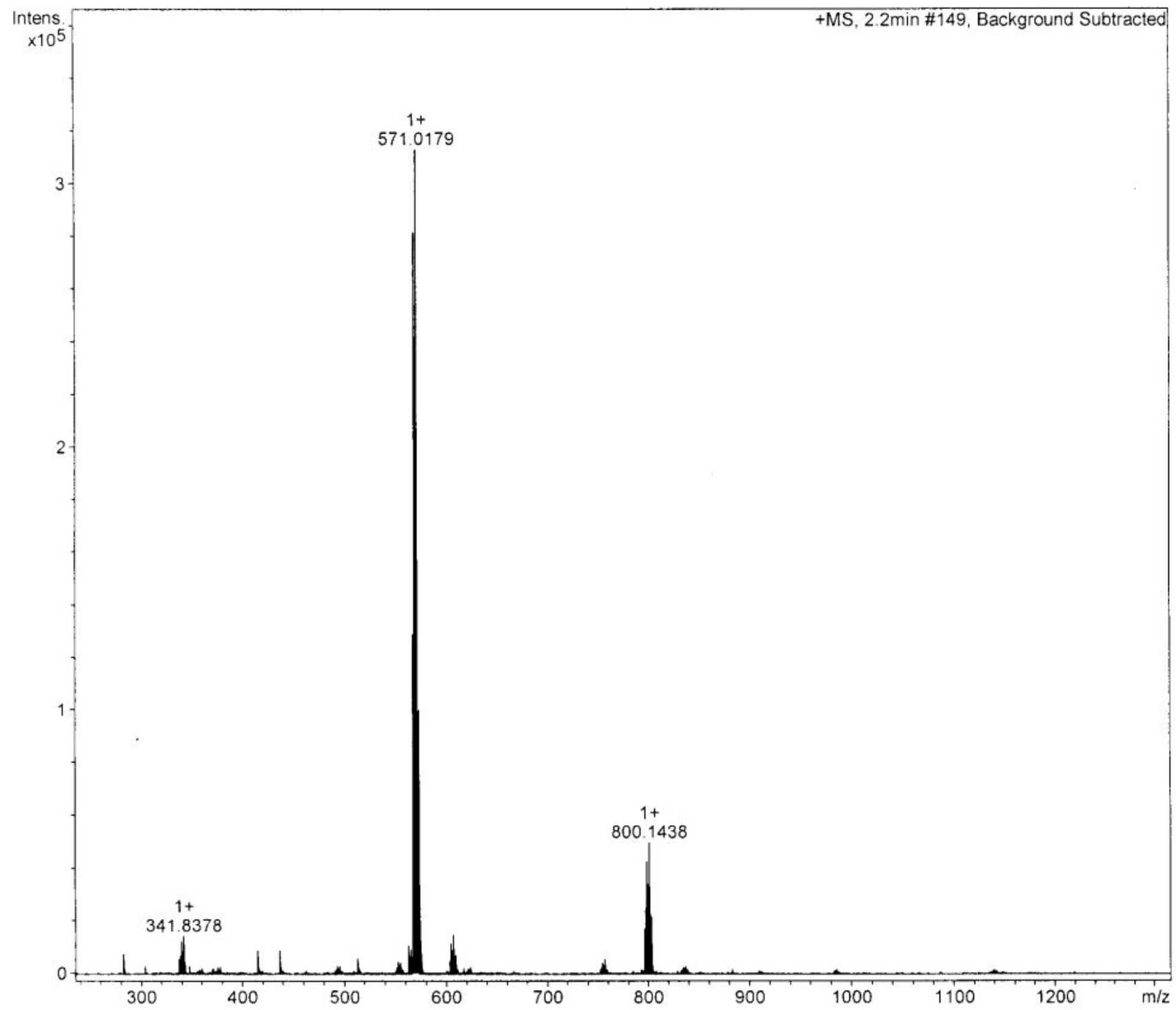

Bruker Compass DataAnalysis 4.0

Figure S4: The EI-MS spectrum of the compound **5**

RIAIDT
UNIDADE DE ESPECTROMETRIA DE MASAS E PROTEÓMICA
Edificio CACTUS * Campus Vida
15782 Santiago de Compostela (Spain)
Tel. 981 563 100. Ext. 16242 * Fax 981 528098
E-mail: sxemasas@usc.es
Web: http://www.ti.usc.es/riaidt/masas/masas.htm

ESPECTRO ESI-FIA-TOF

Analysis Info

Sample Name	CD11_640_1-D,10_01_9991.d $[CdCl_2(Hnatsc)_2]\cdot H_2O$ (**6**$\cdot H_2O$)	Acquisition Date	6/4/2013 8:19:14 PM
Method	fia_pos_medio_350-1500_egf.m	Instrument	micrOTOF

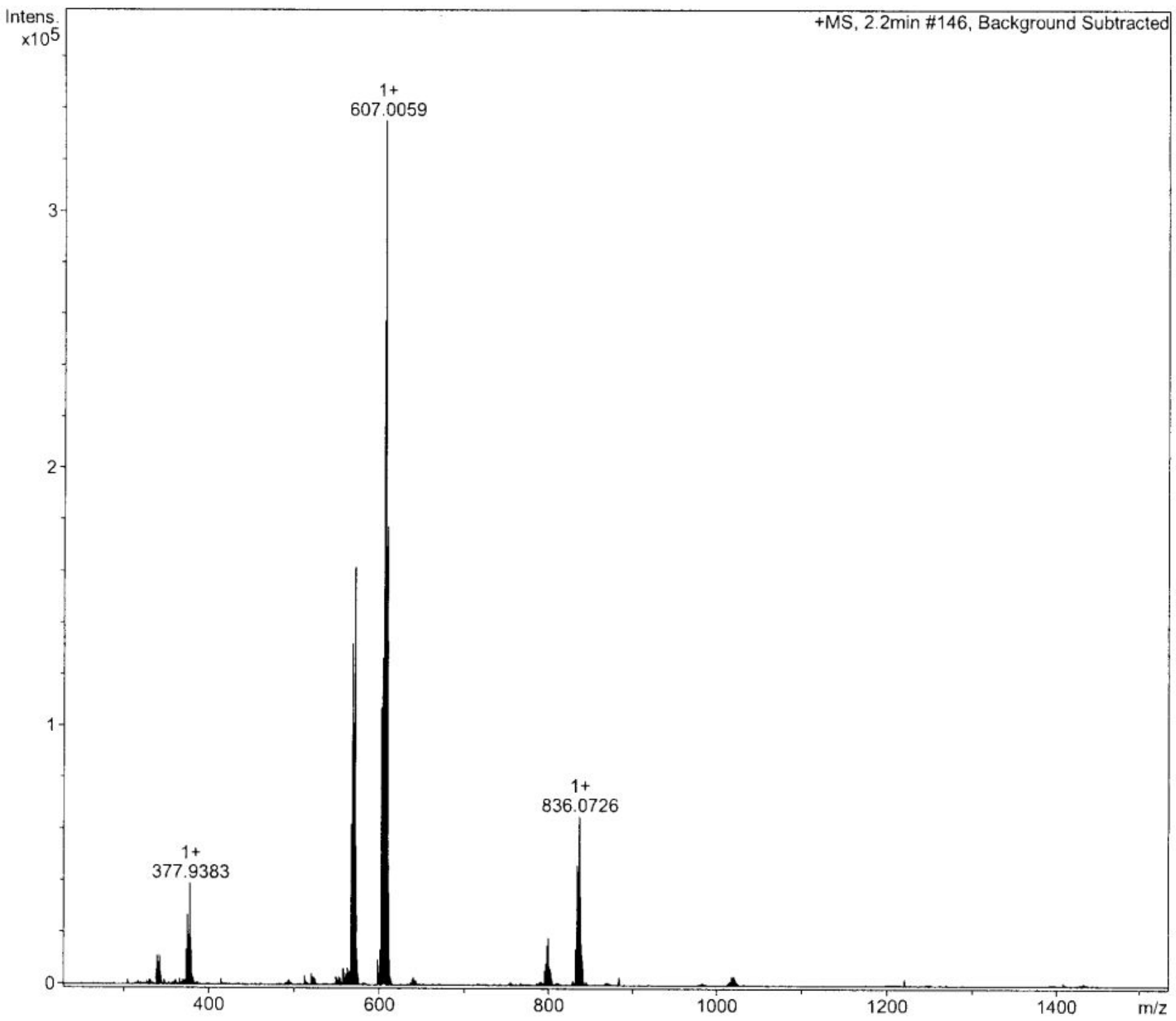

Bruker Compass DataAnalysis 4.0

Figure S5: The EI-MS spectrum of the compound **6**

FT-IR Spectra

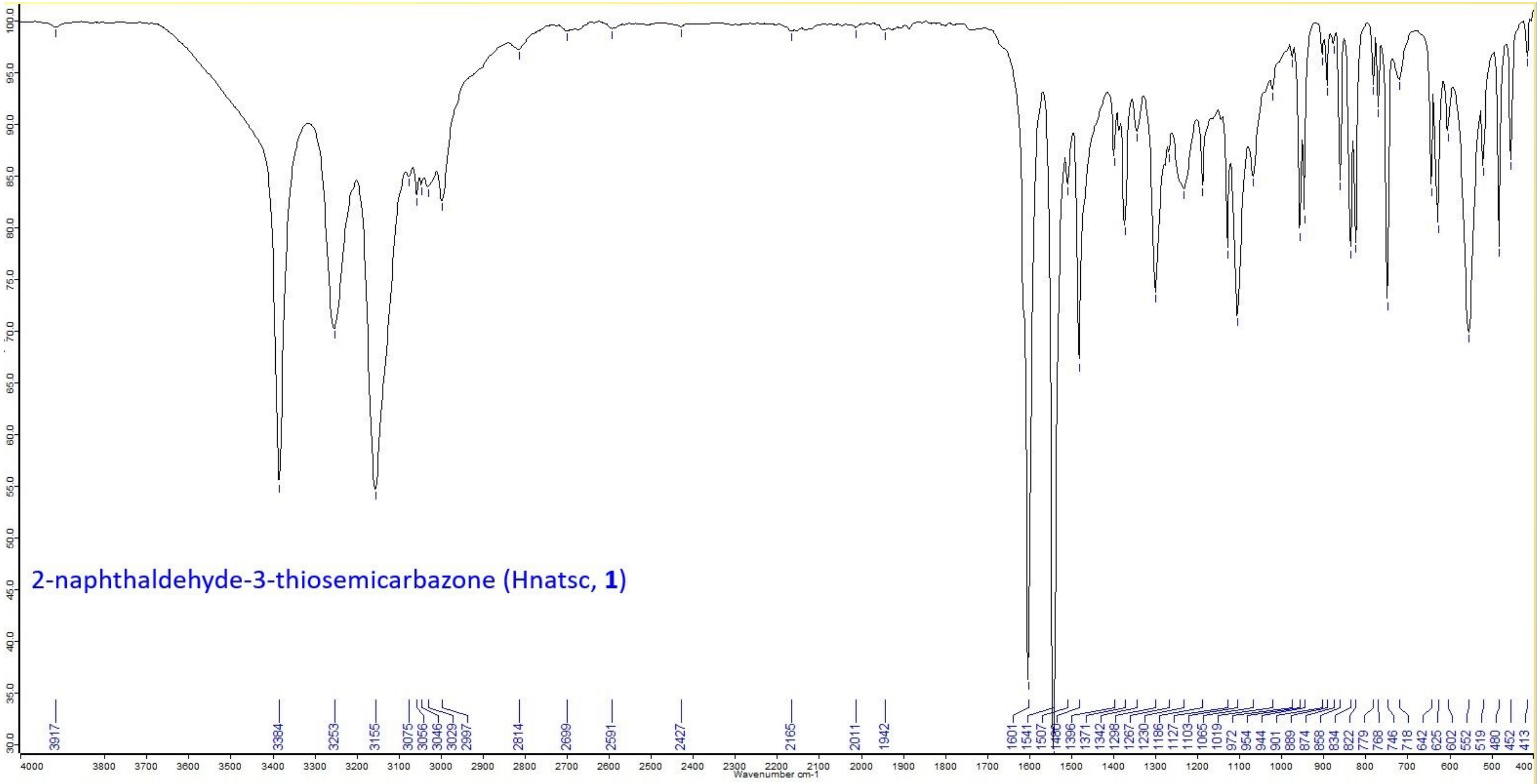

Figure S6. FT-IR spectrum for **1** between 4000 and 400 cm^{-1}.

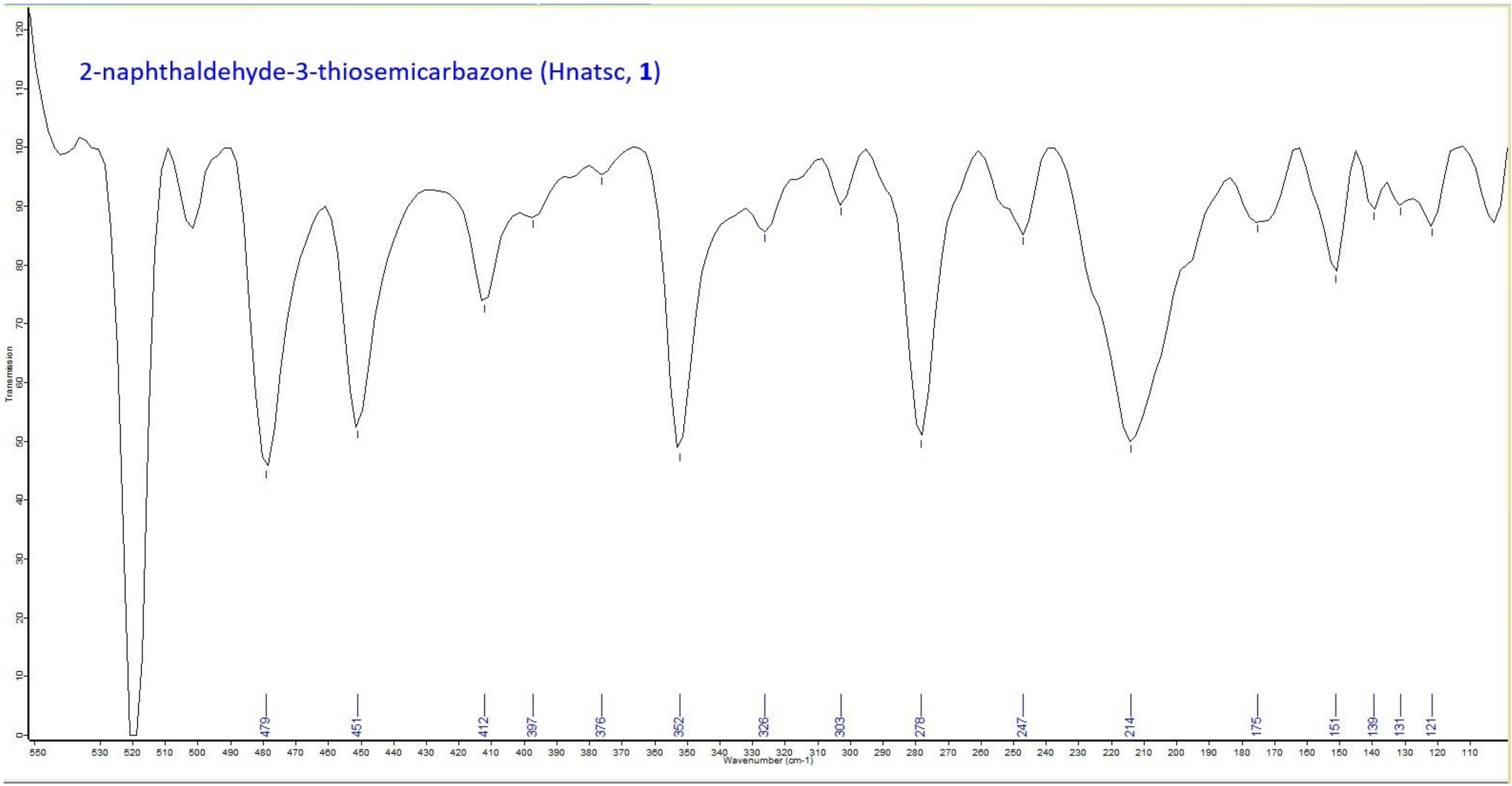

Figure S7. FT-IR spectrum for **1** between 550 and 100 cm^{-1}

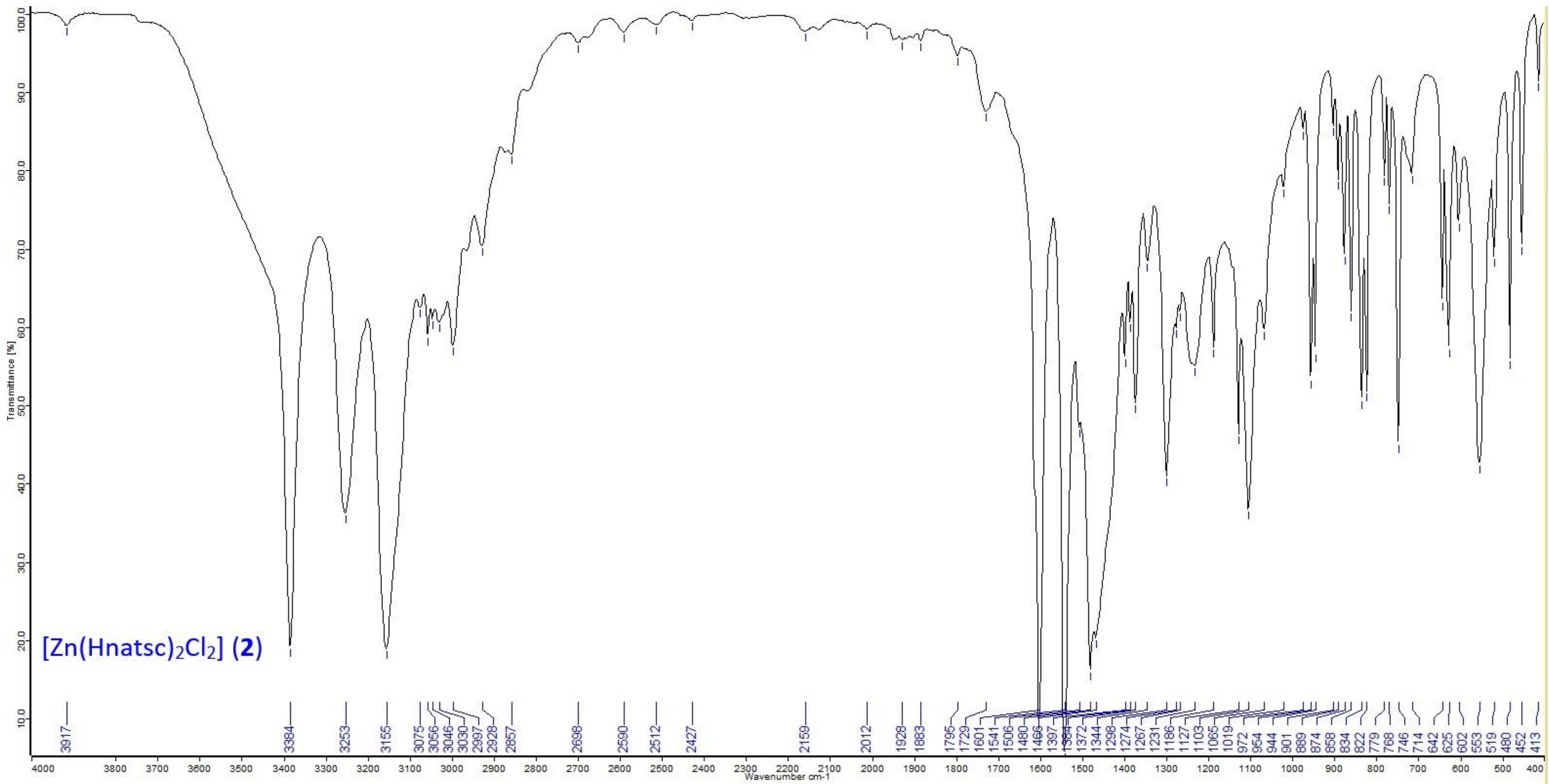

Figure S8. FT-IR spectrum for **2** between 4000 and 400 cm^{-1}

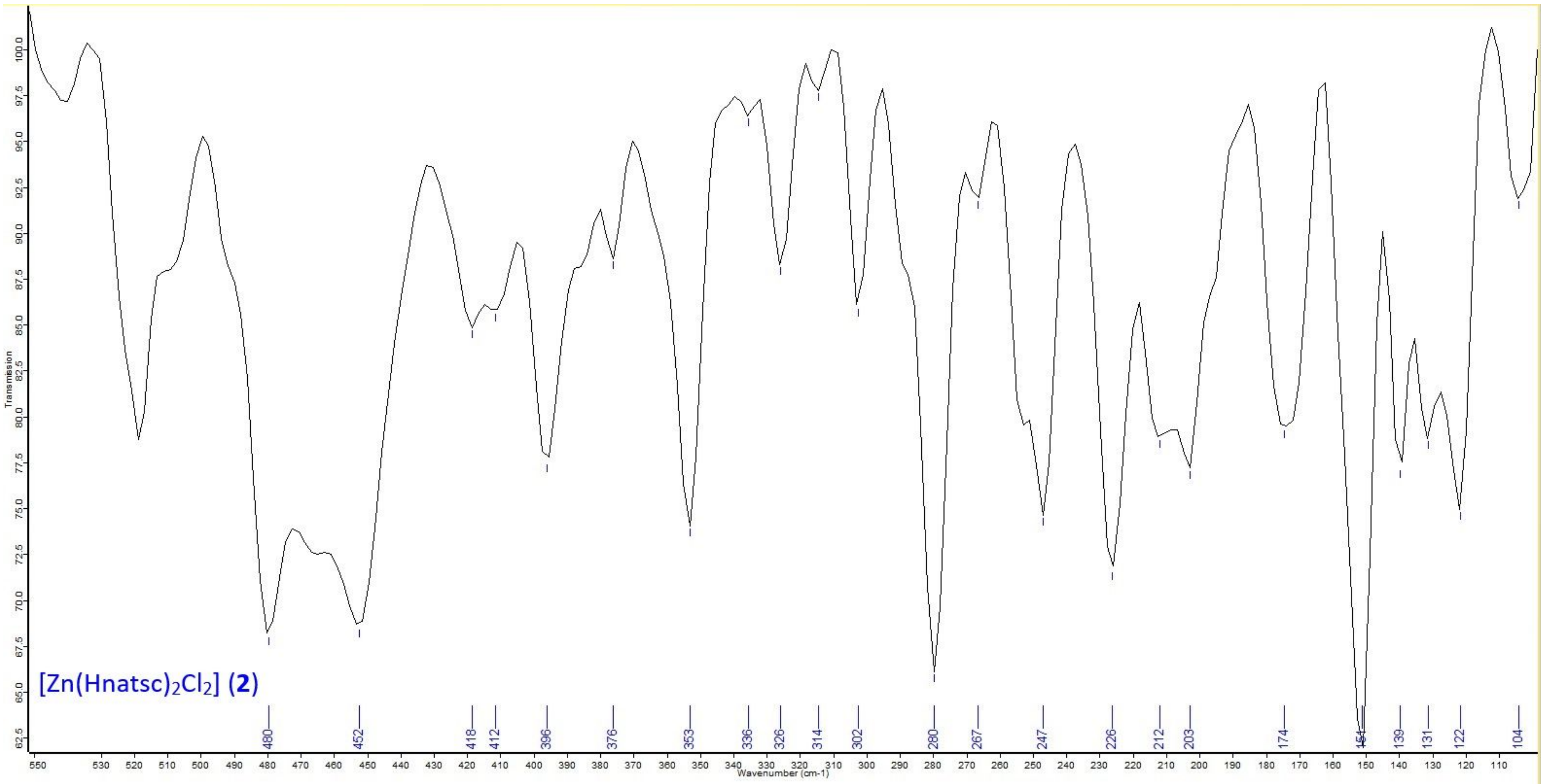

Figure S9. FT- IR spectrum for **2** between 550 and 100 cm^{-1}

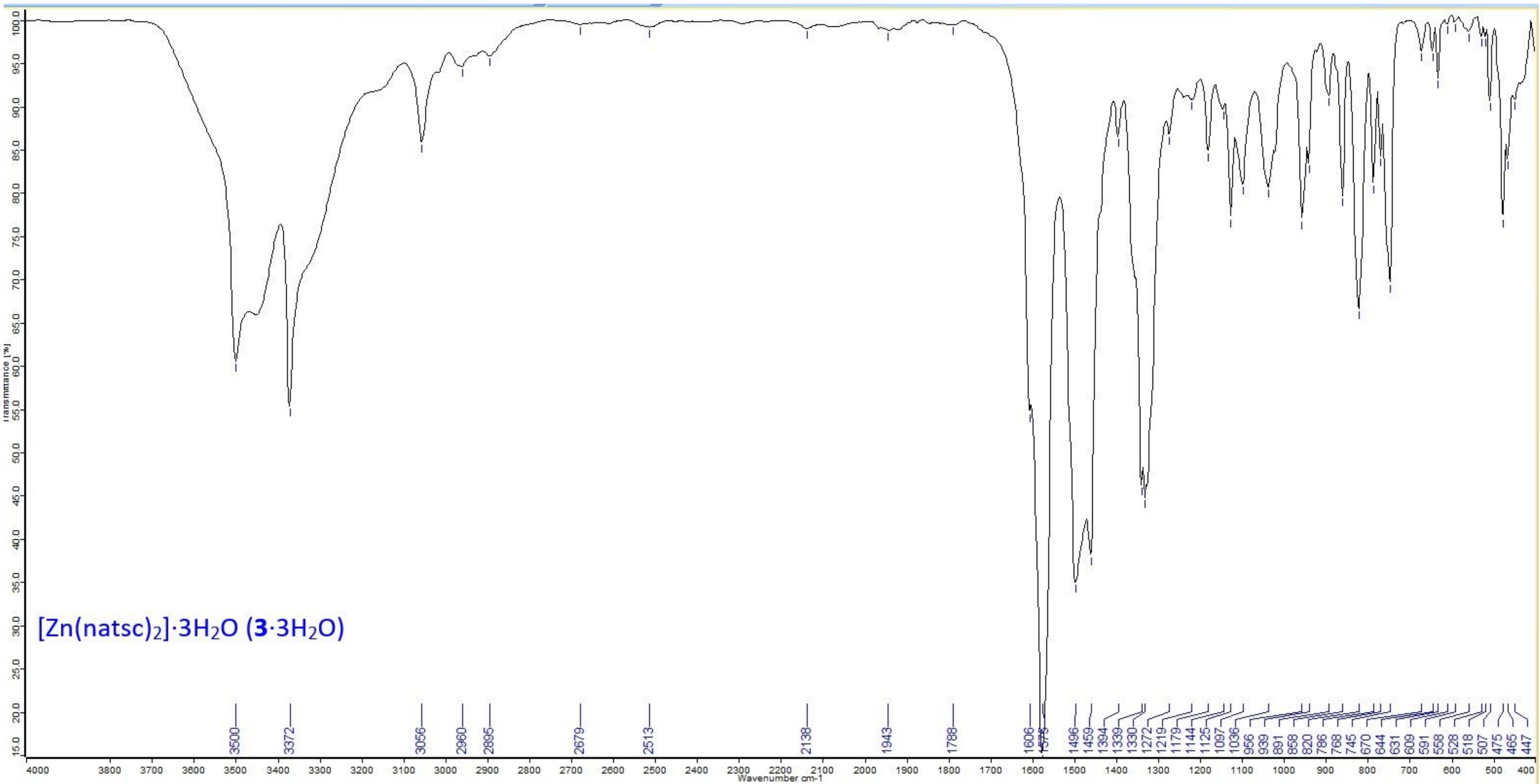

Figure S10. FT-IR spectrum for **3** between 4000 and 400 cm^{-1}

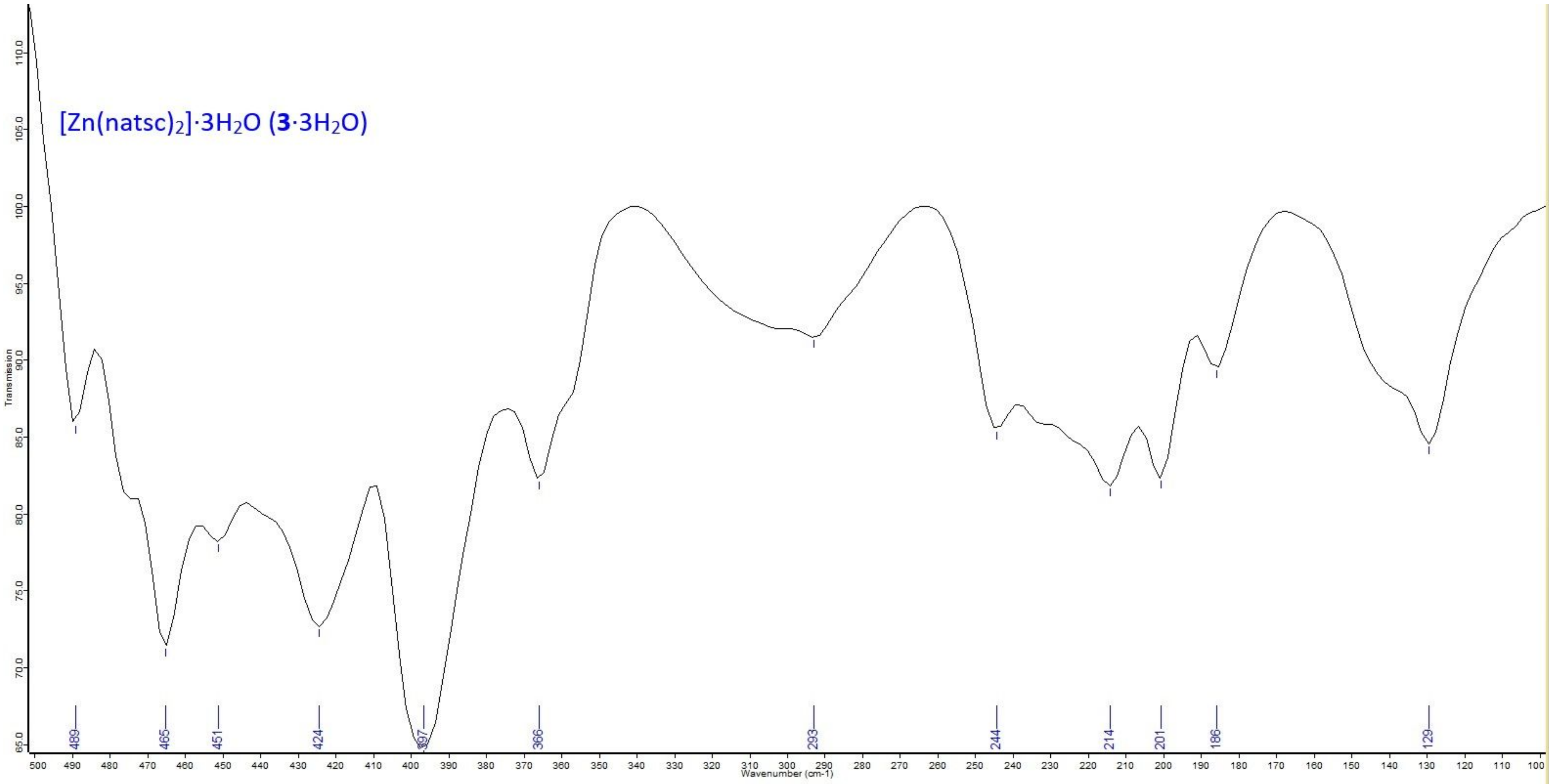

Figure S11. FT-IR spectrum for **3** between 500 and 100 cm^{-1}

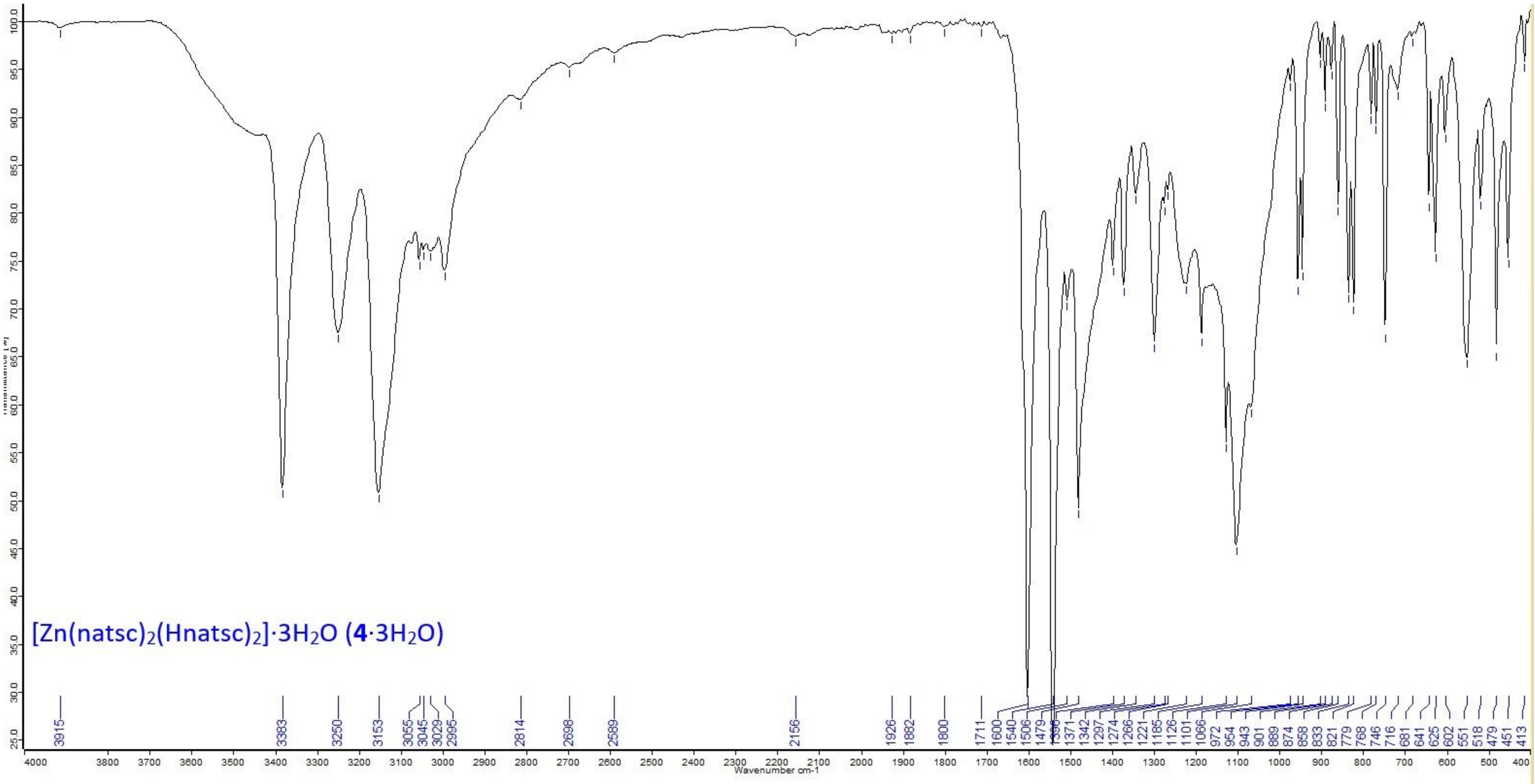

Figure S12. FT-IR spectrum for **4** between 4000 and 400 cm^{-1}

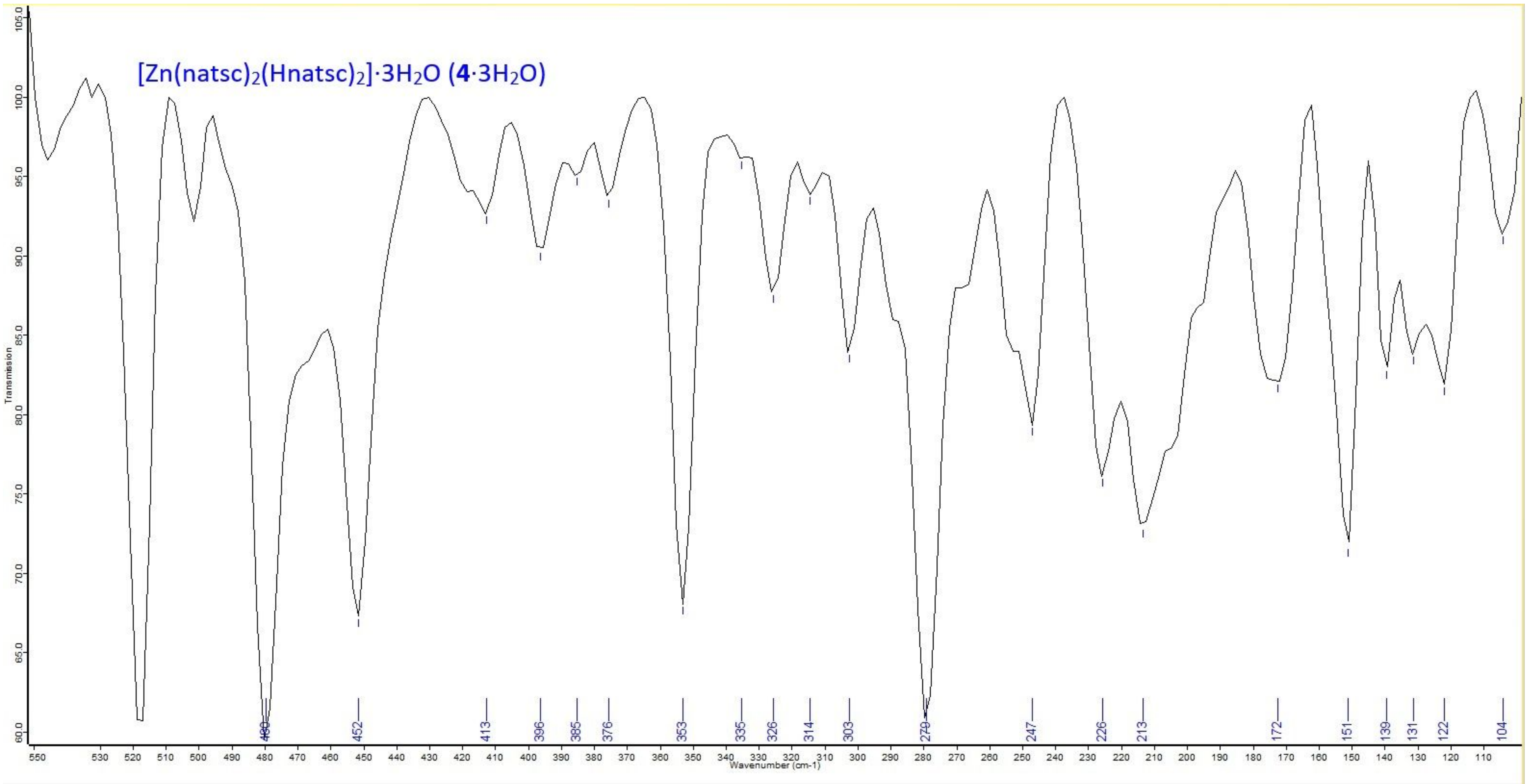

Figure S13. FT-IR spectrum for **4** between 550 and 100 cm^{-1}

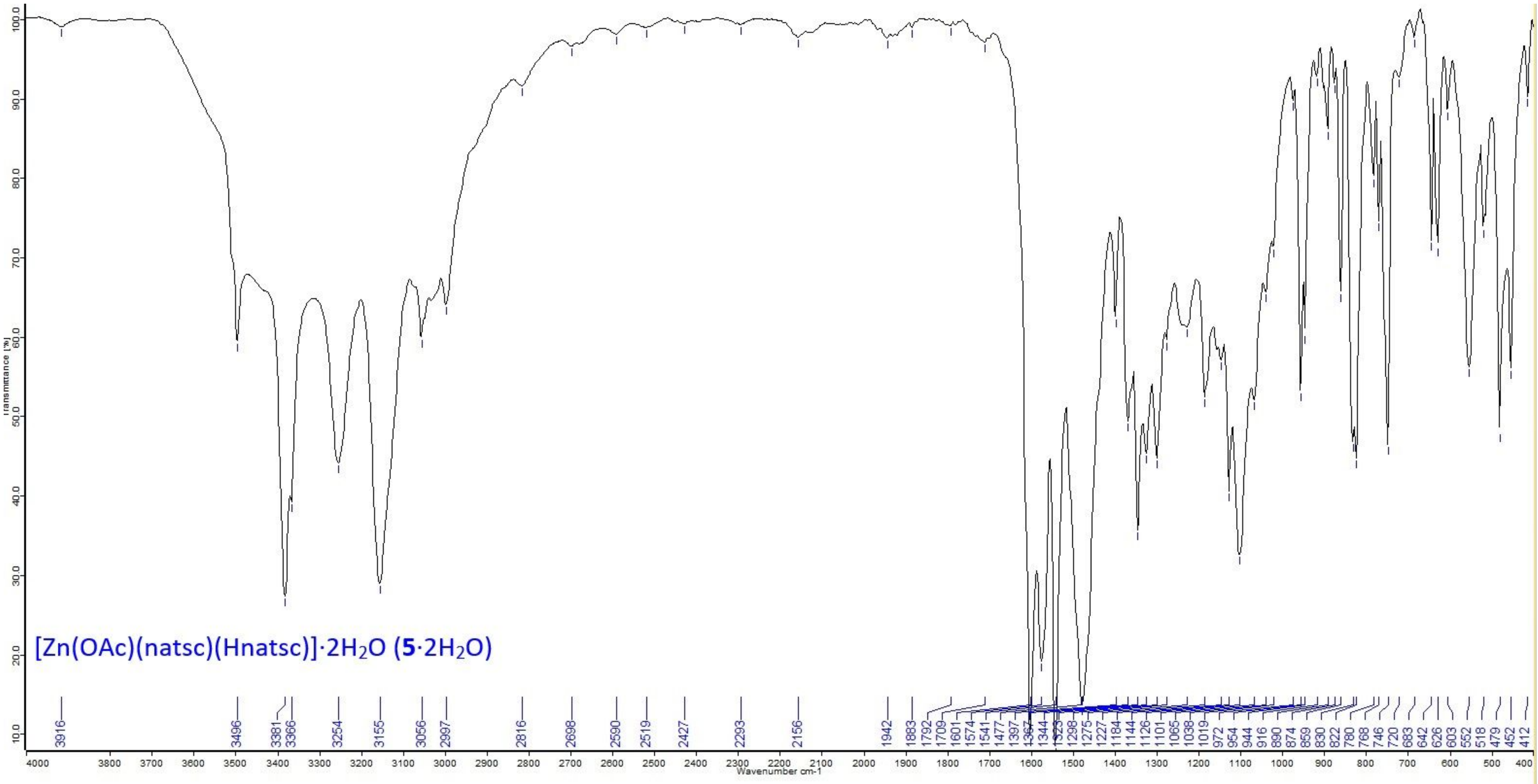

Figure S14. FT-IR spectrum for **5** between 4000 and 400 cm^{-1}

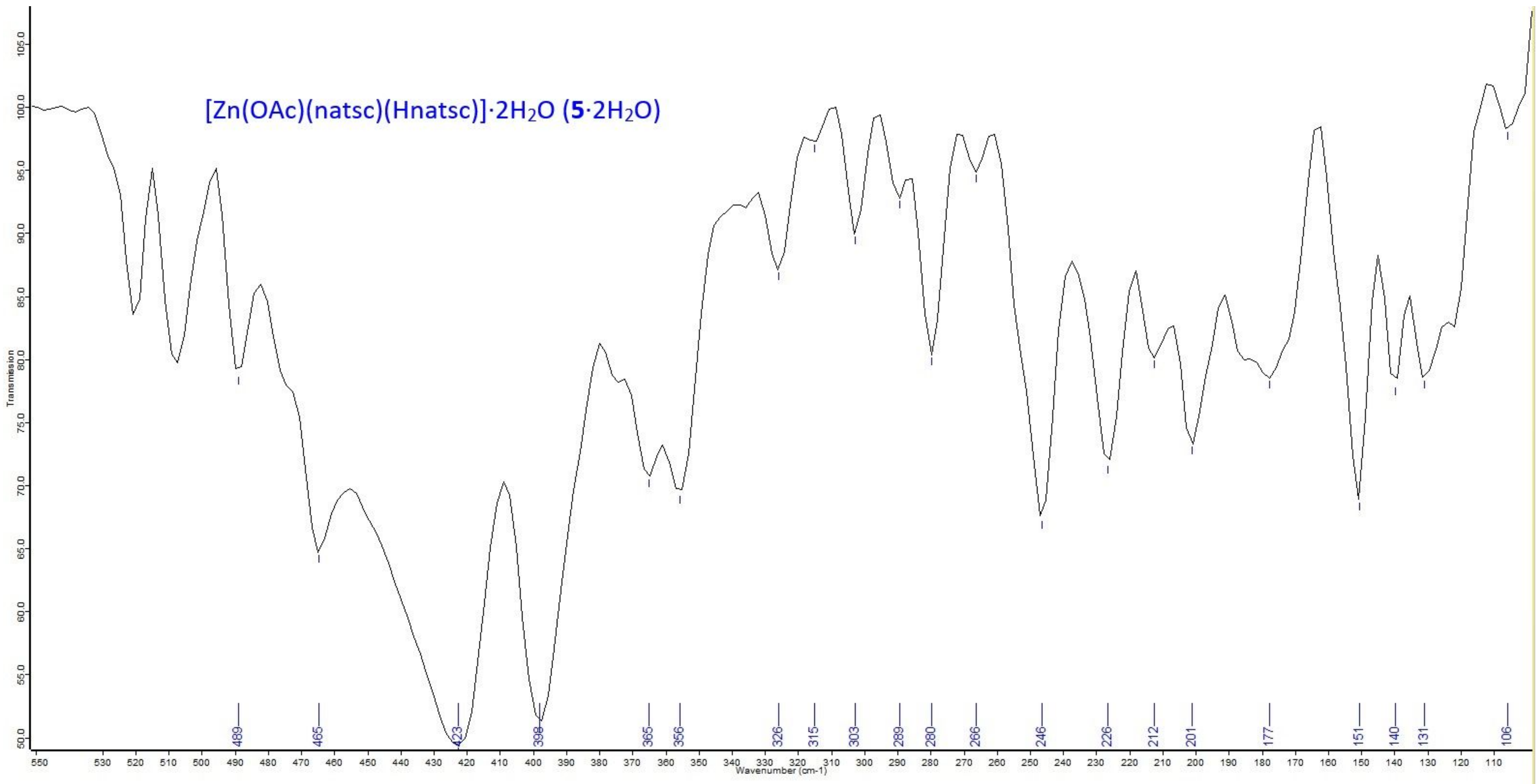

Figure S15. FT-IR spectrum for **5** between 550 and 100 cm^{-1}

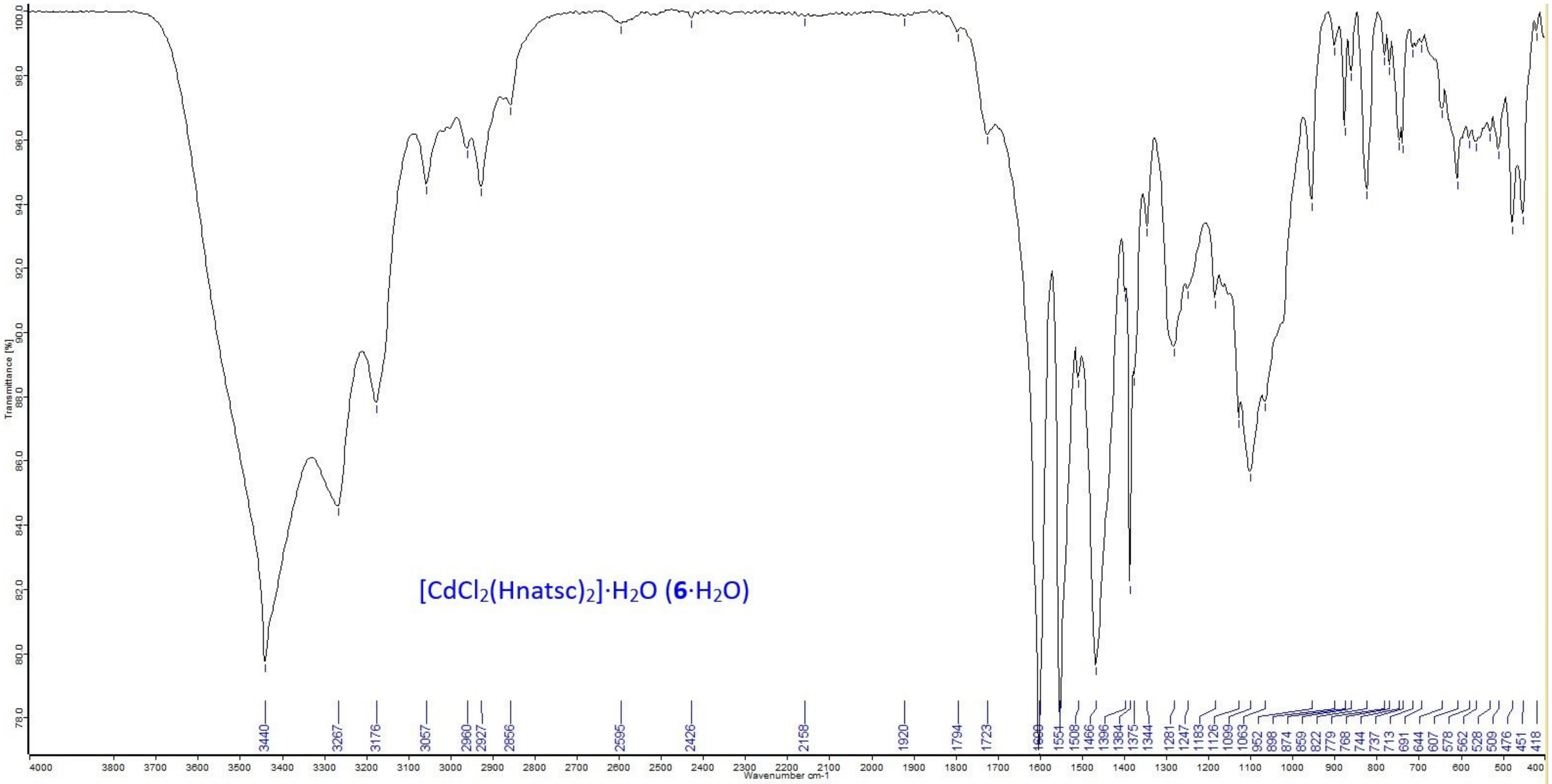

Figure S16. FT-IR spectrum for **6** between 4000 and 400 cm^{-1}

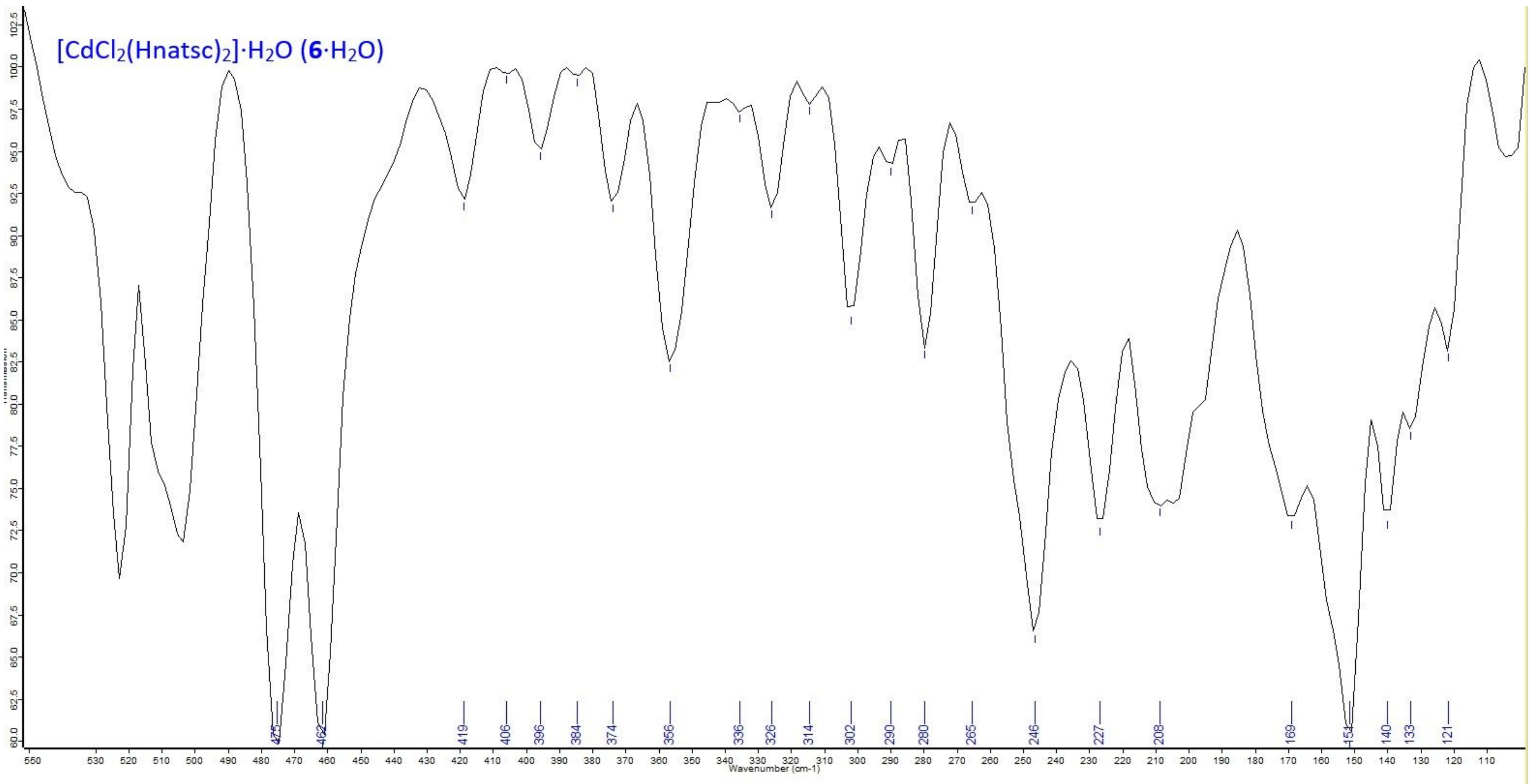

Figure S17. FT-IR spectrum for **6** between 550 and 100 cm^{-1}

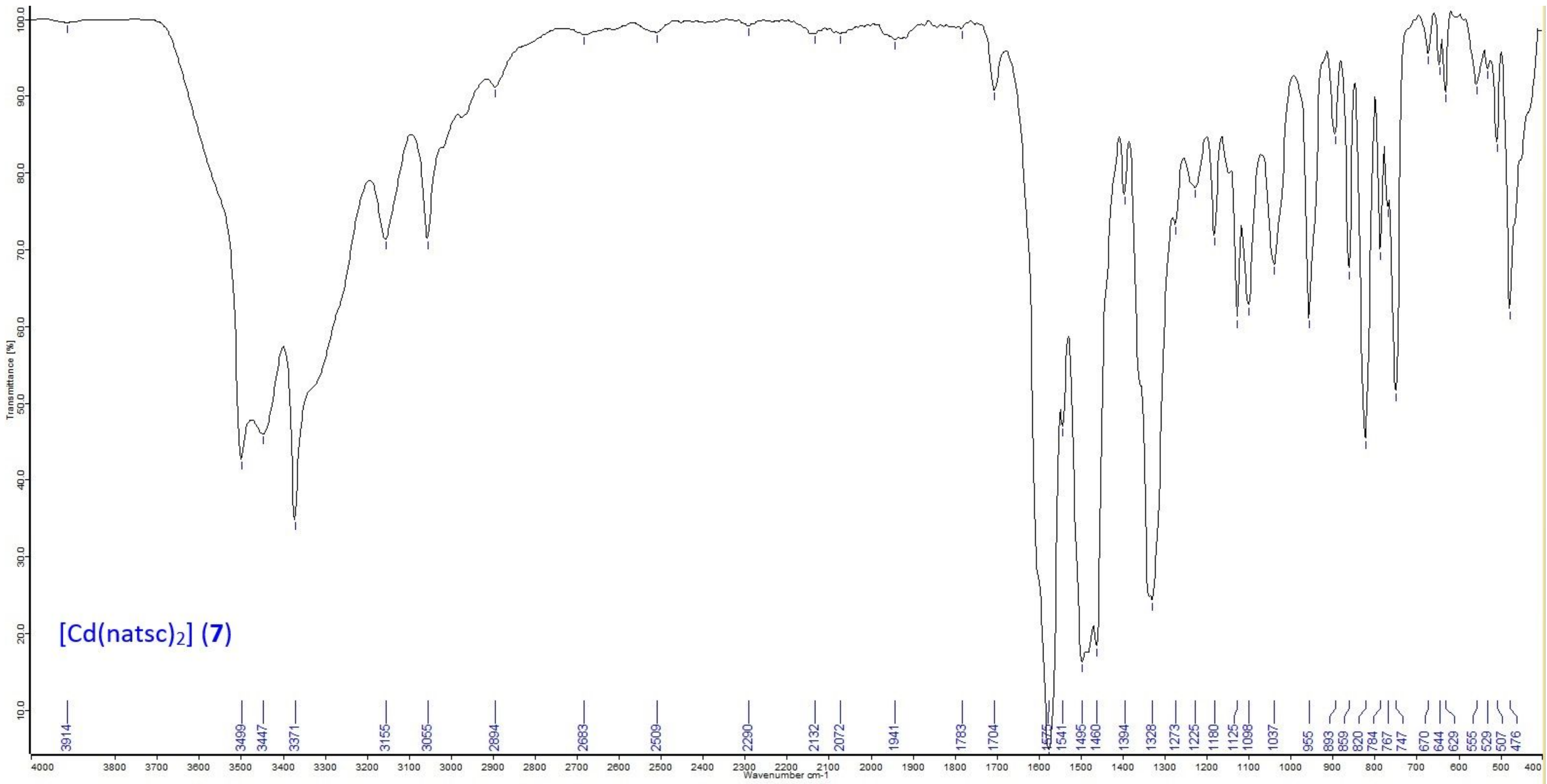

Figure S18. FT-IR spectrum for **7** between 4000 and 400 cm^{-1}

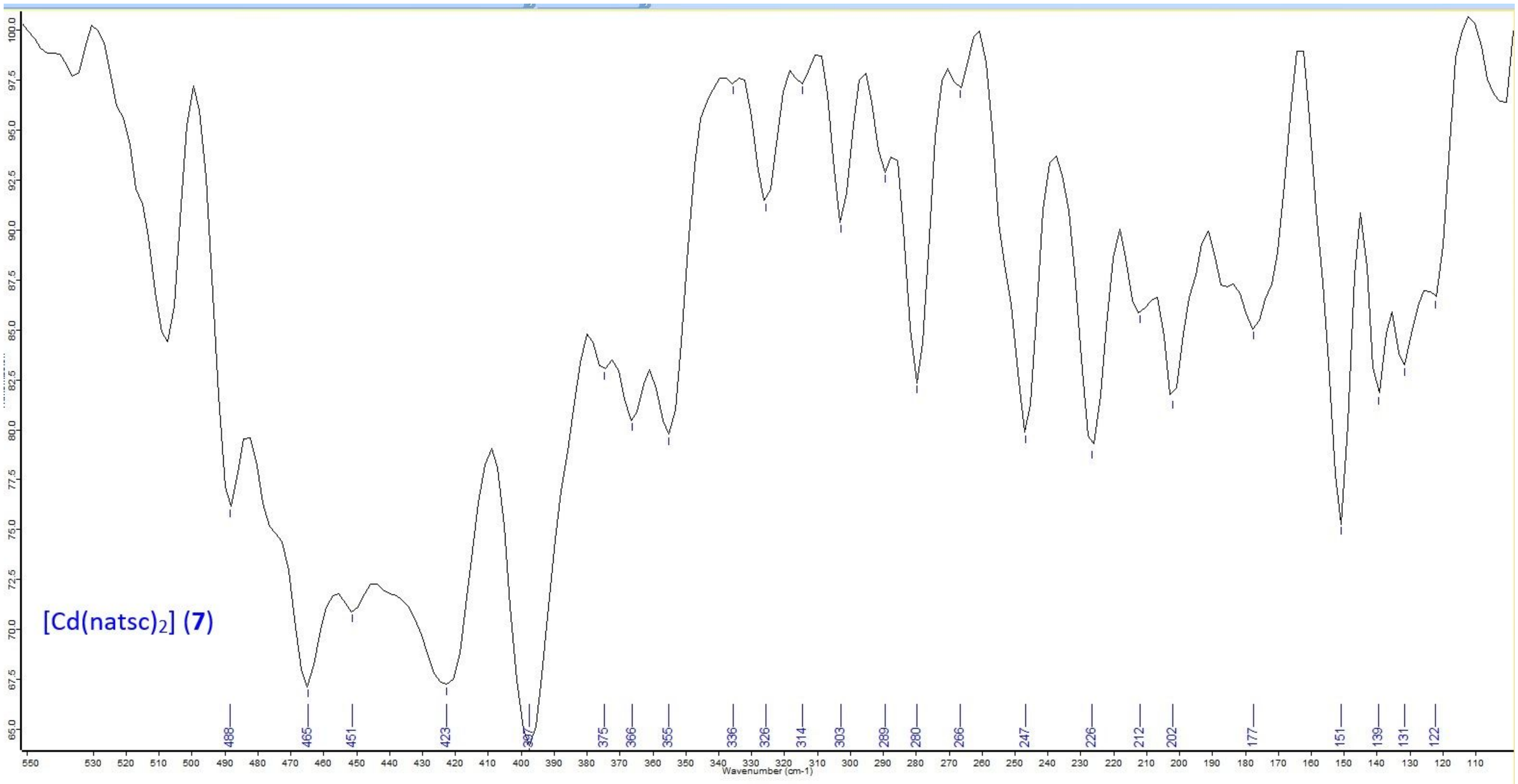

Figure S19. FT-IR spectrum for **7** between 550 and 100 cm^{-1}

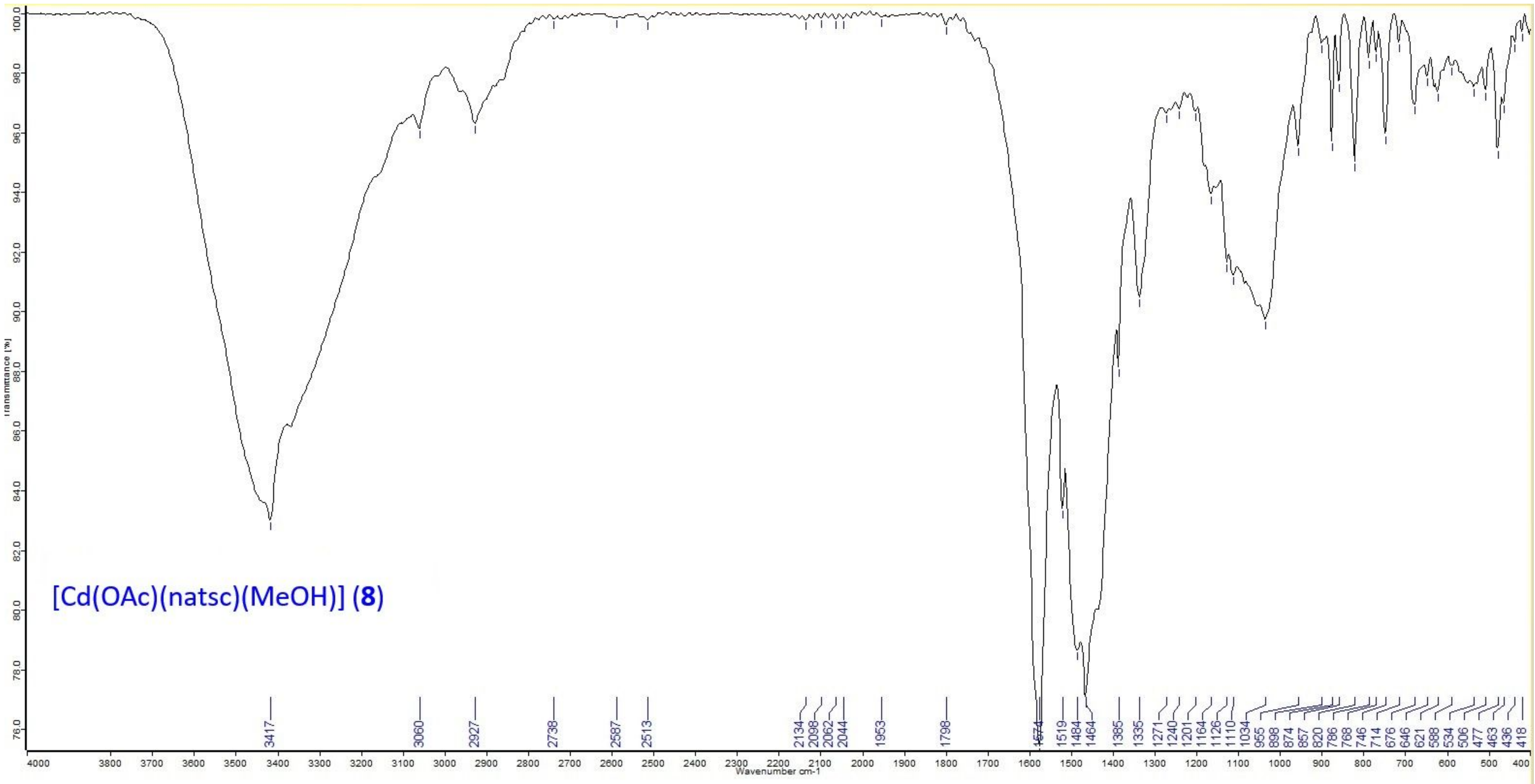

Figure S20. FT-IR spectrum for **8** between 4000 and 400 cm^{-1}

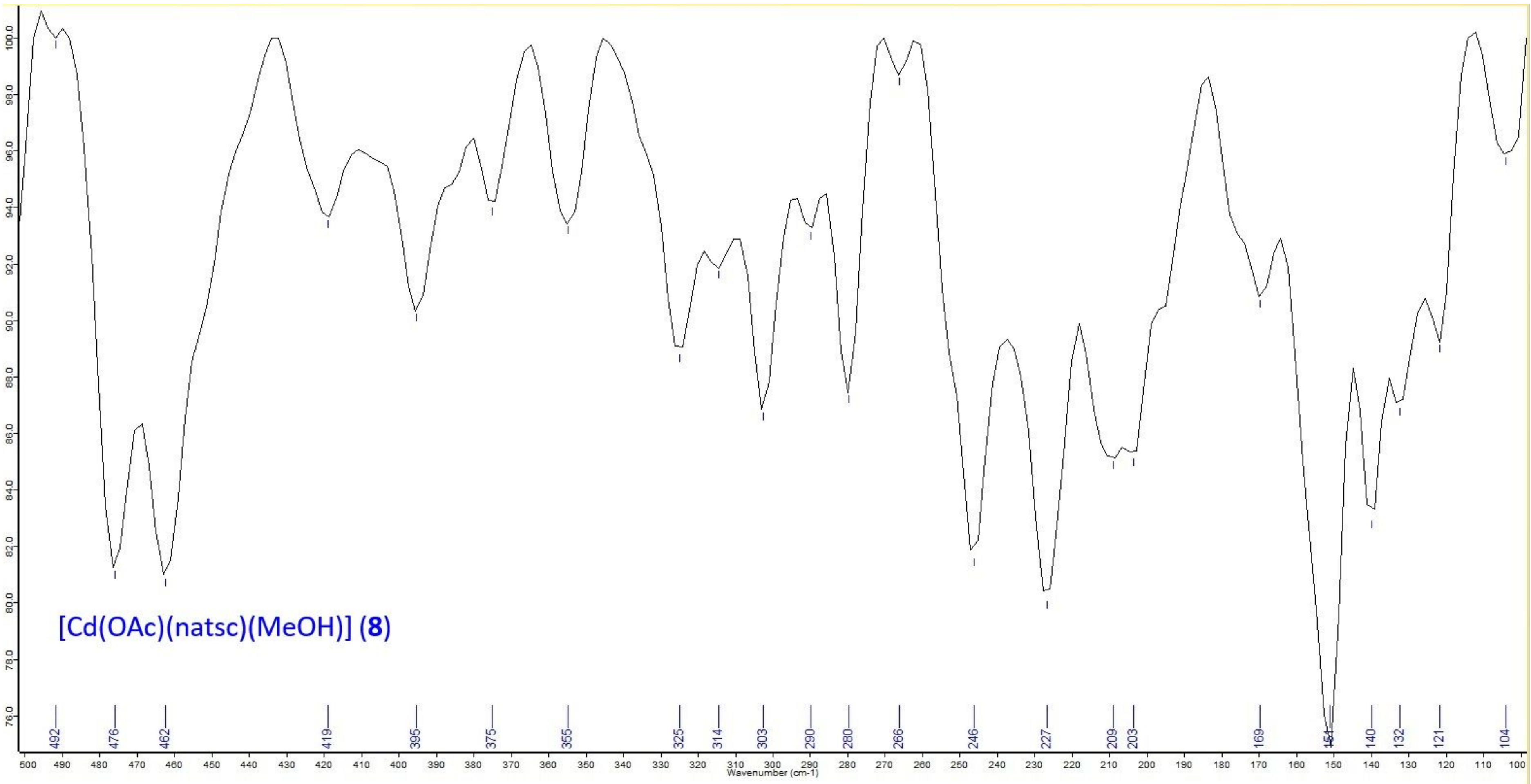

Figure S21. FT-IR spectrum for **8** between 500 and 100 cm^{-1}

^{1}H NMR Spectra

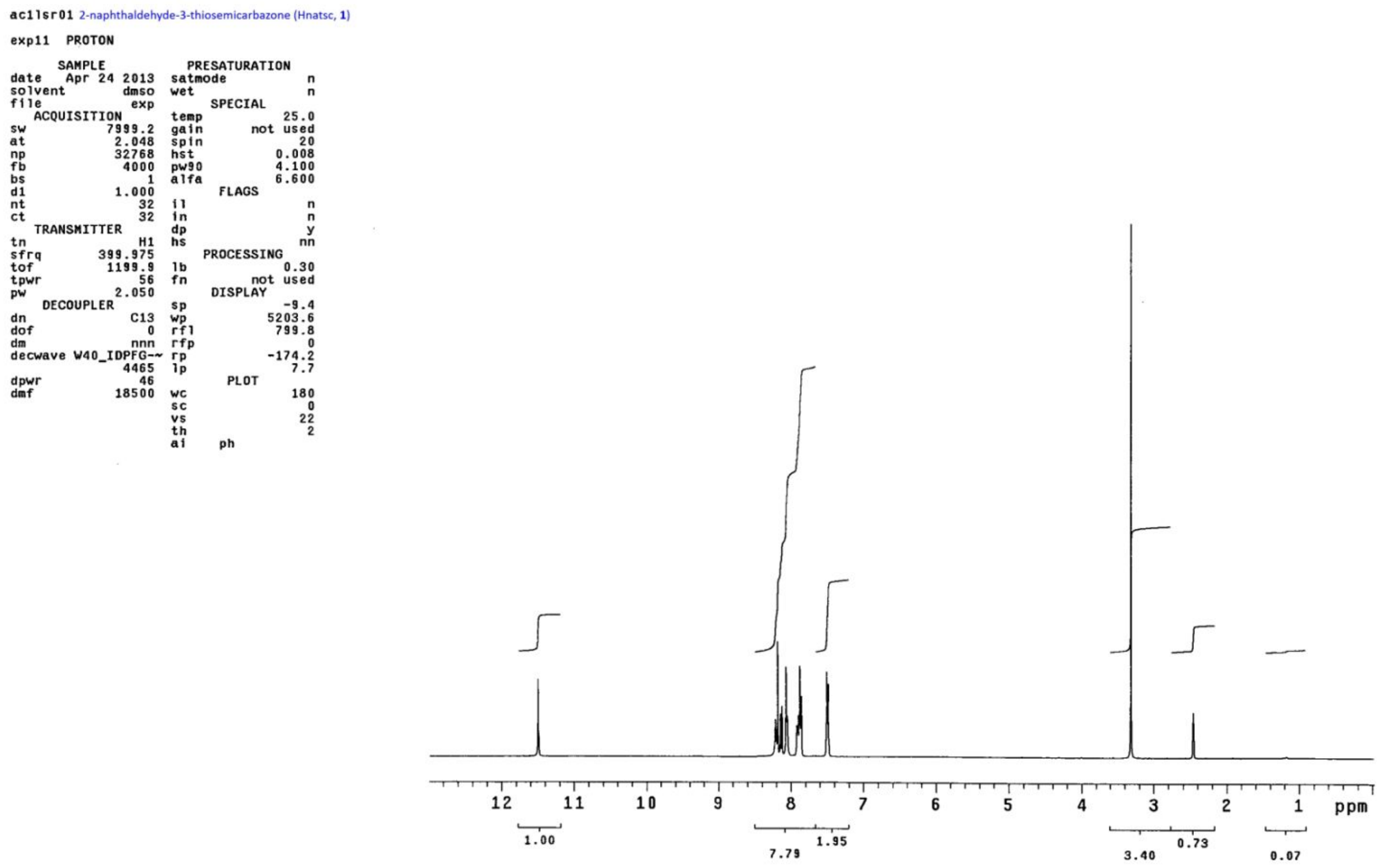

Figure S22. ^{1}H NMR spectrum of **1** in DMSO-d^6 (300 MHz).

In the ^{1}H NMR spectrum of **1** in DMSO-d^6 (Figure S22), the C-H signal appears as a singlet at 8.18 ppm. The ring protons (H1-H9) appear between 7.50 and 8.20 ppm. The most deshielded ring signal corresponds to H9, although two distinct signals appear for this proton, clearly indicating the presence of two conformers in solution, characteristic of naphthyl-imine derivatives[1,2].

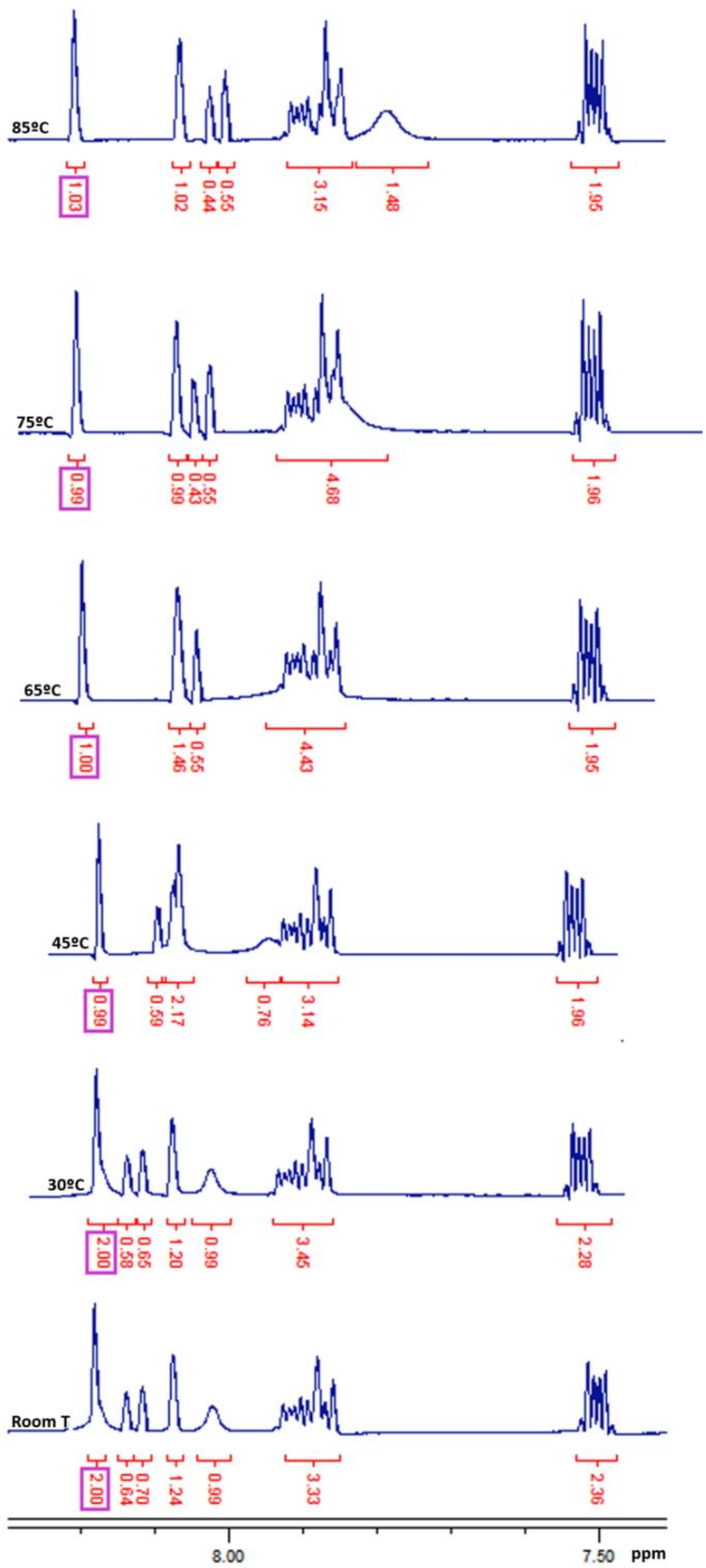

Figure S23. ^{1}H NMR spectrum of the Hnatsc ligand at different temperatures

However, it is important to note that only one signal appears for the other C-H groups (Figure S22). Therefore, the appearance of two signals for H9 can be attributed to the different orientation of H9 with respect to the C=N bond in the two conformers. In one of the conformers, H9 is oriented in such a way that it falls within the shielding region of the C=N bond (anisotropy cone), experiences a lower effective magnetic field, and resonates at a lower δ value. In the other conformer, H9 lies outside this region and therefore retains a higher chemical shift.This results in the appearance of two distinct resonances for H9, one corresponding to each conformer.

An anisotropy cone predicts that any nucleus situated above the double bond is shielded, and in the plane of the double bond deshielded[3]. In contrast, protons H1–H8 do not significantly change their relative position with respect to the C=N bond when moving from one conformer to another. The difference in chemical shift between the two conformers for these protons is very small, so their resonances overlap and a single signal is observed for each of them. In summary, there is a conformational equilibrium throughout the molecule, but only H9 is sensitive enough to conformational change for both magnetic environments to be experimentally resolved. To demonstrate the existence of the two conformers in DMSO, a ^{1}H NMR spectrum study was performed at different temperatures, observing their presence even at high temperatures (Figure S23).

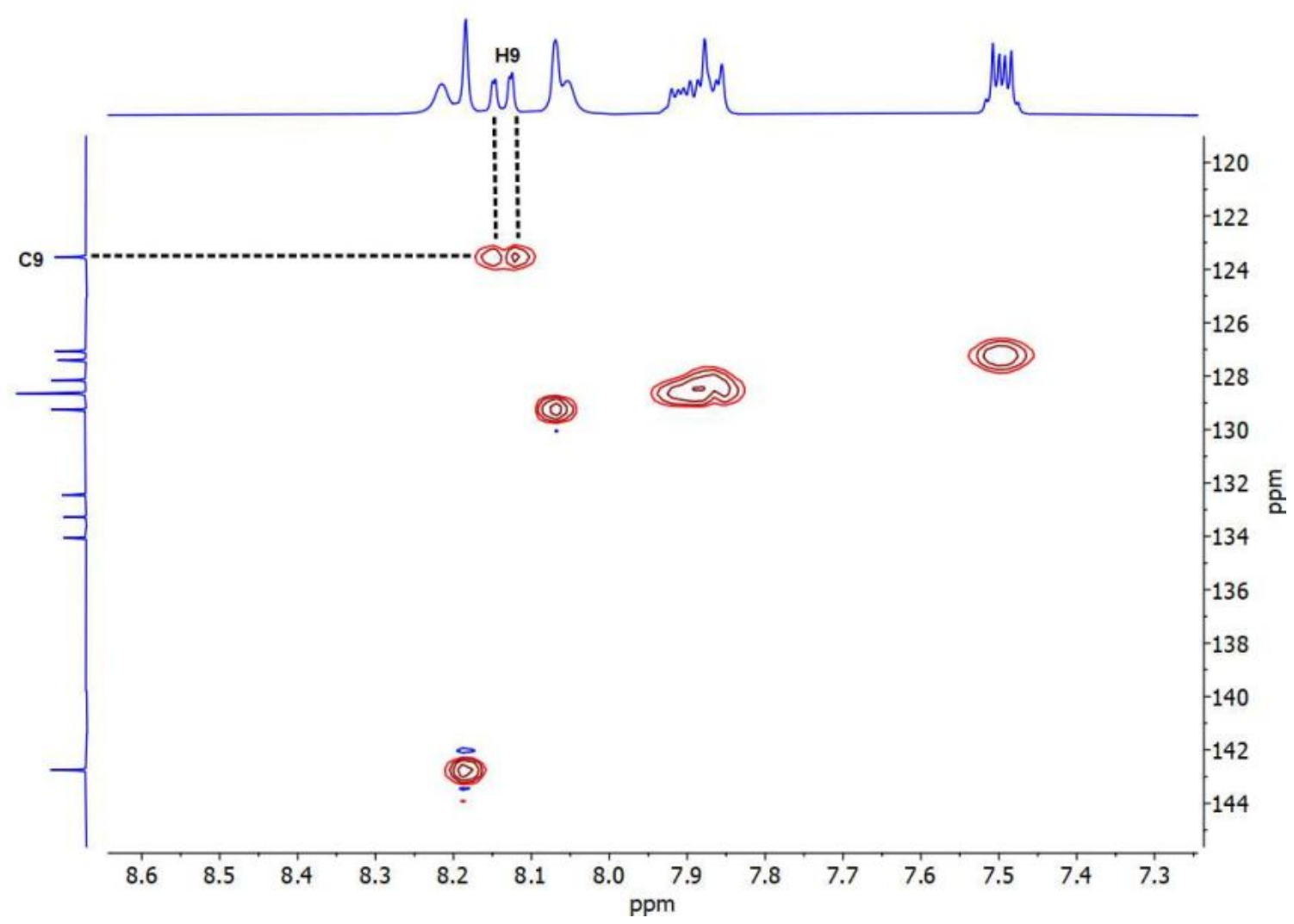

Figure S24. Hnatsc Heteronuclear Multiple Bond Correlation (HMBC) Spectrum

In any case, this assignment is unequivocal because both H9 protons show correlation with the same C atom in the HMQC spectrum (Figure S24).

References

[1] Casarini, D.; Lunazzi, L.; Macciantelli, D. Conformational studies by dynamic NMR. Part 48. Conformational preferences and stereomutational processes of the rotational enantiomers of hindered naphthylimines and imonium salts. *J. Chem. Soc., Perkin Trans.* 2. **1992**, *21*, 1363–1370. https://doi.org/10.1039/P29920001363

[2] Hamor, T. A.; Jennings, W. B.; Proctor, L. D.; Tolley, M. S.; Boyd, D. R.; Mullan, T. Imines and derivatives. Part 23. Anomalous ^{1}H NMR spectrum of N-[1-(1-naphthyl)ethylidene]-1-phenyl-2-propylamine: conformation in solution, atropisomerism and an X-ray crystal structure *J. Chem. Soc., Perkin Trans.* 2. **1990**, *19*, 25–30. https://doi.org/10.1039/P29900000025

[3] Baranac-Stojanovic, M. New insight into the anisotropic effects in solution-state NMR spectroscopy *RSC Adv.* **2015**, *4*, 308–321. https://doi.org/10.1039/c3ra45512b

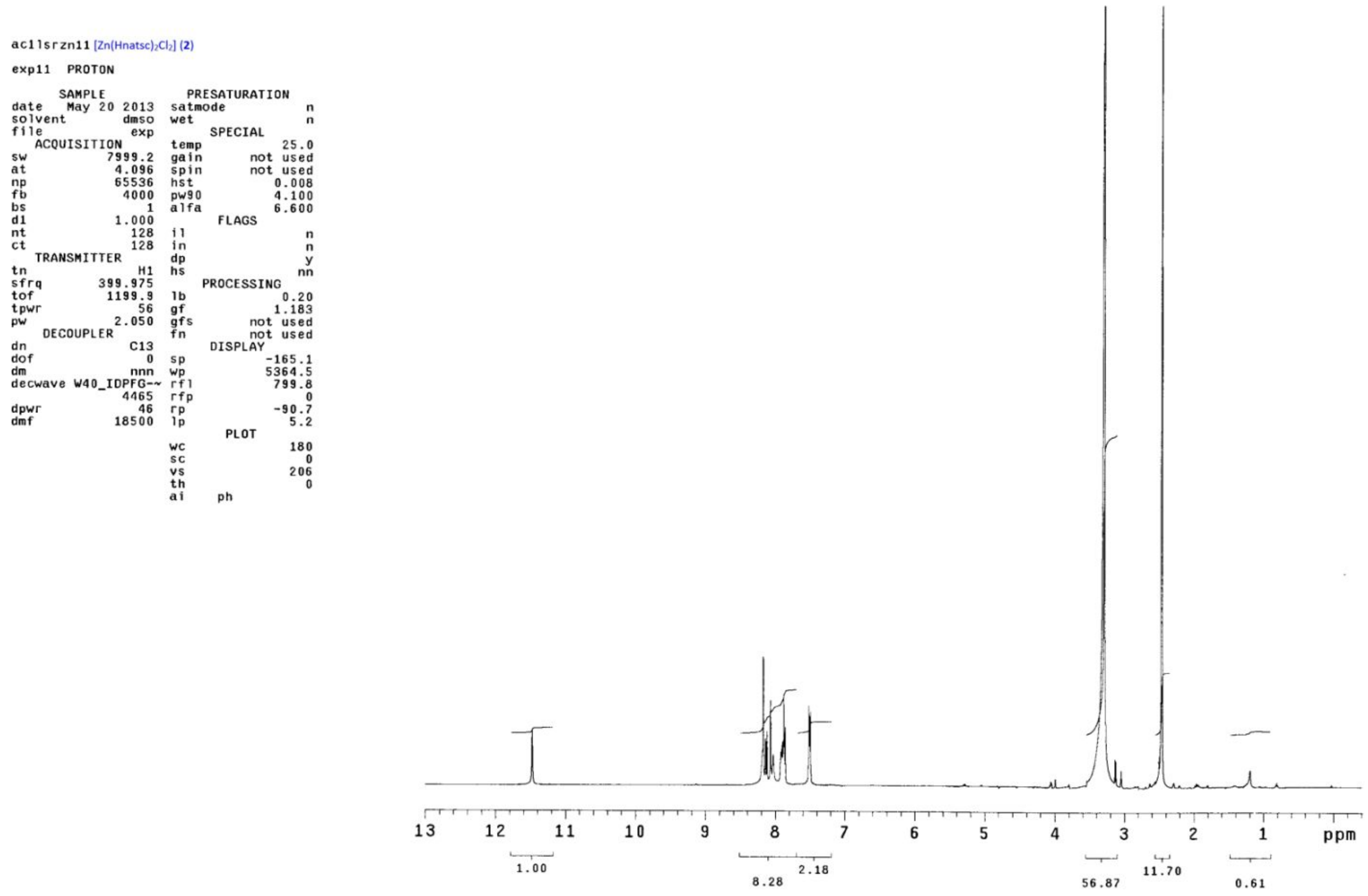

Figure S25. ^{1}H NMR spectrum of **2** in DMSO-d^6 (300 MHz).

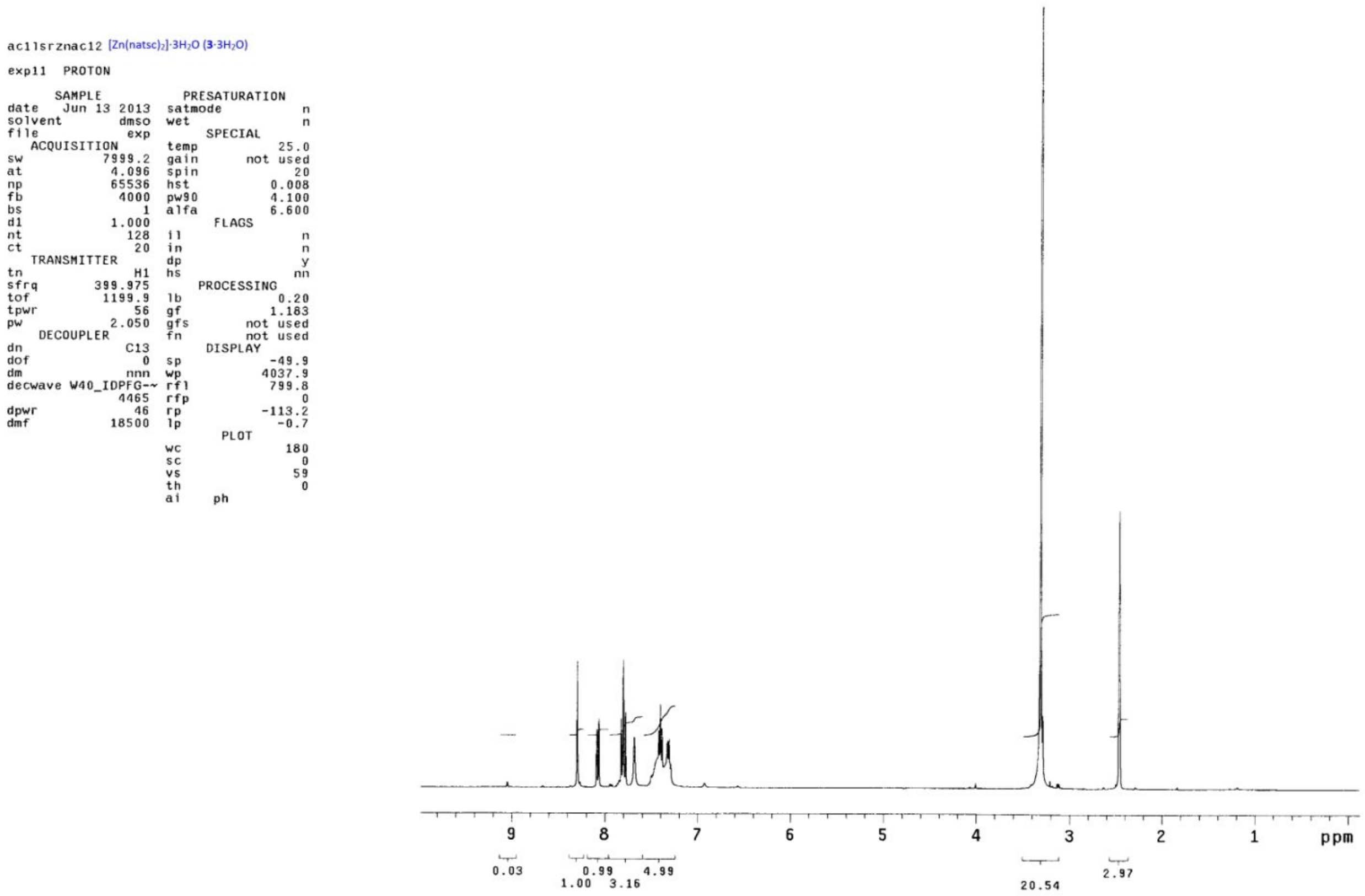

Figure S26. ^{1}H NMR spectrum of **3** in DMSO-d^{6} (300 MHz).

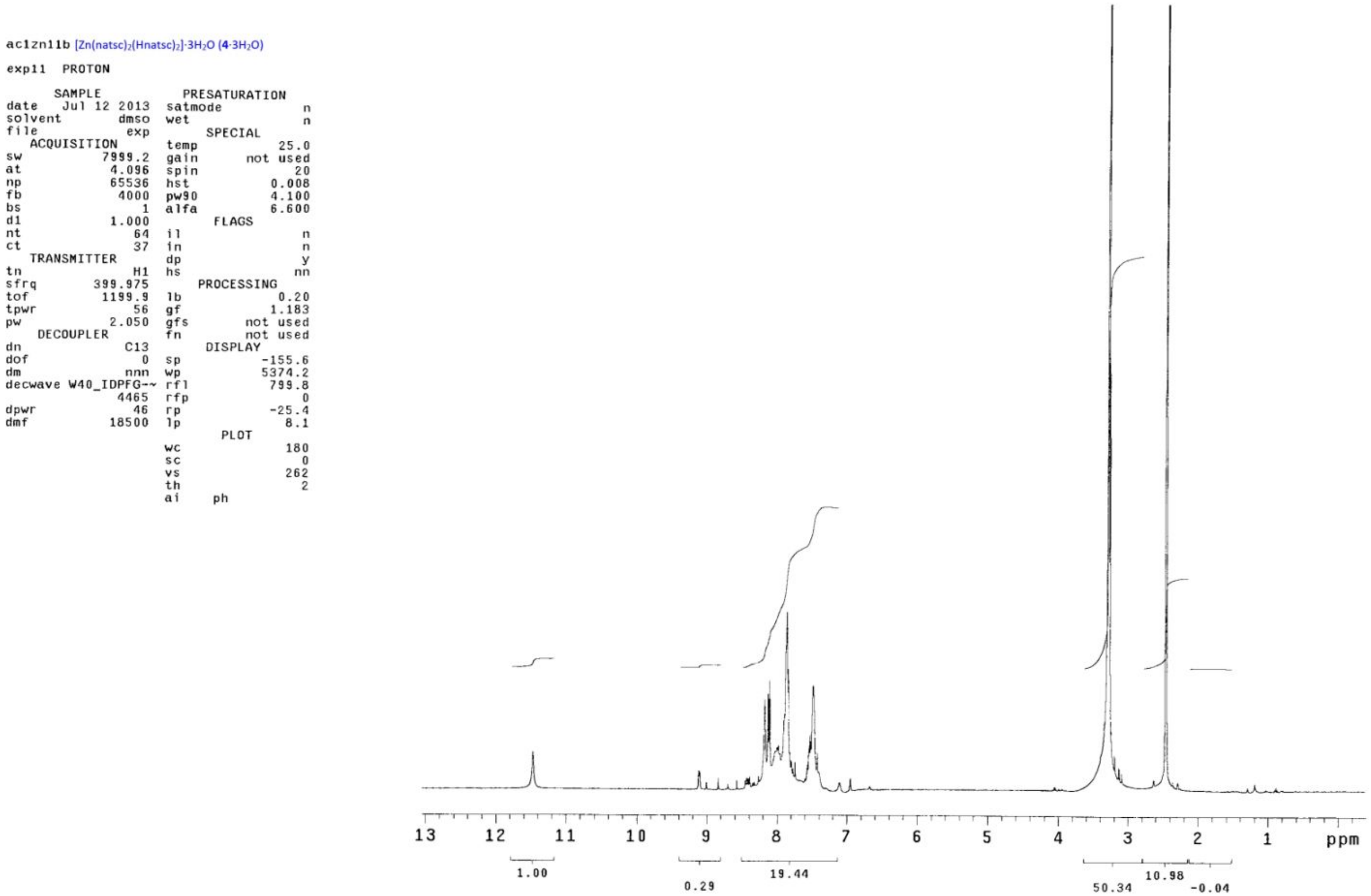

Figure S27. ^{1}H NMR spectrum of **4** in DMSO-d^6 (300 MHz).

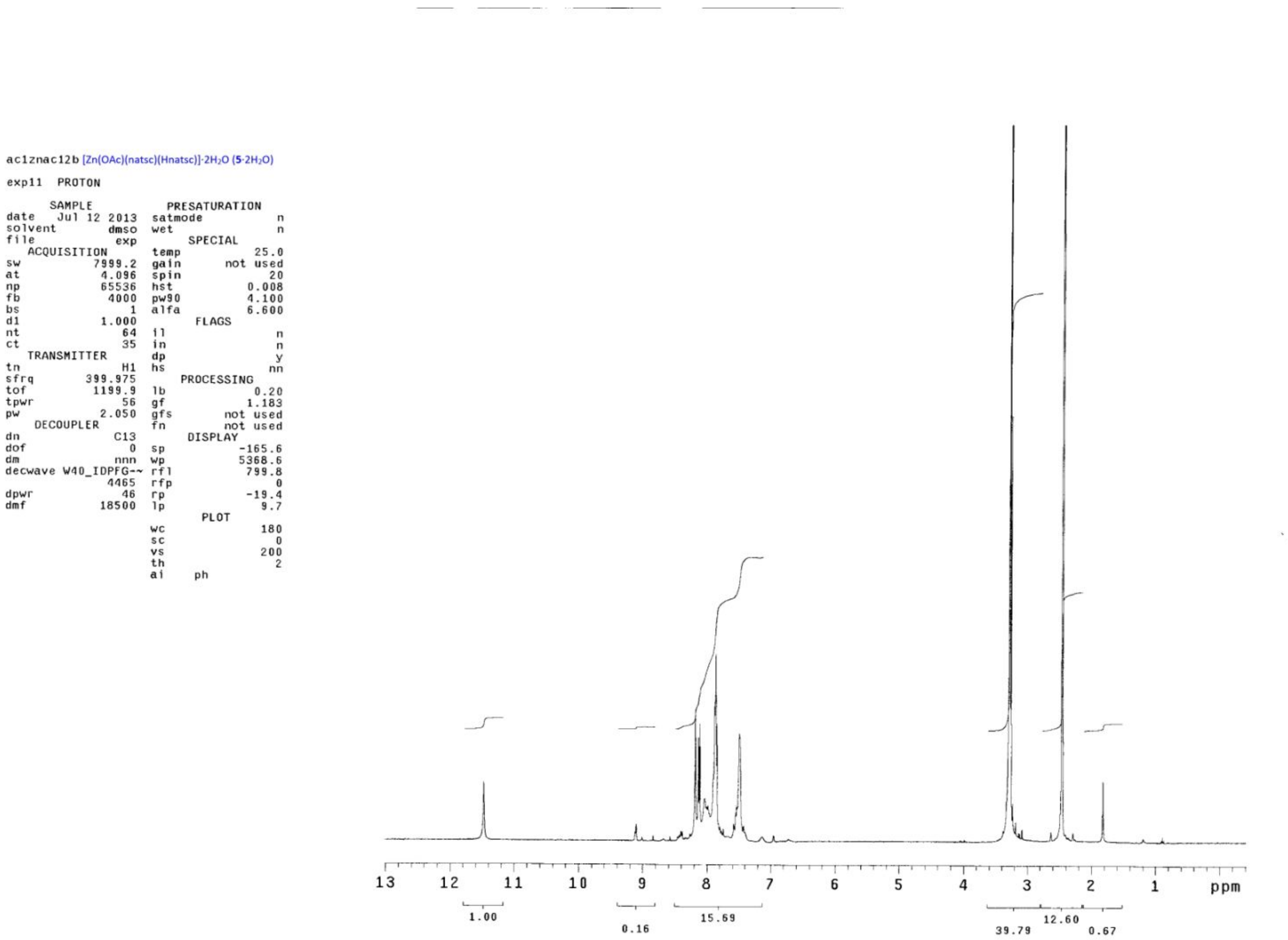

Figure S28. ^{1}H NMR spectrum of **5** in DMSO-d[6] (300 MHz).

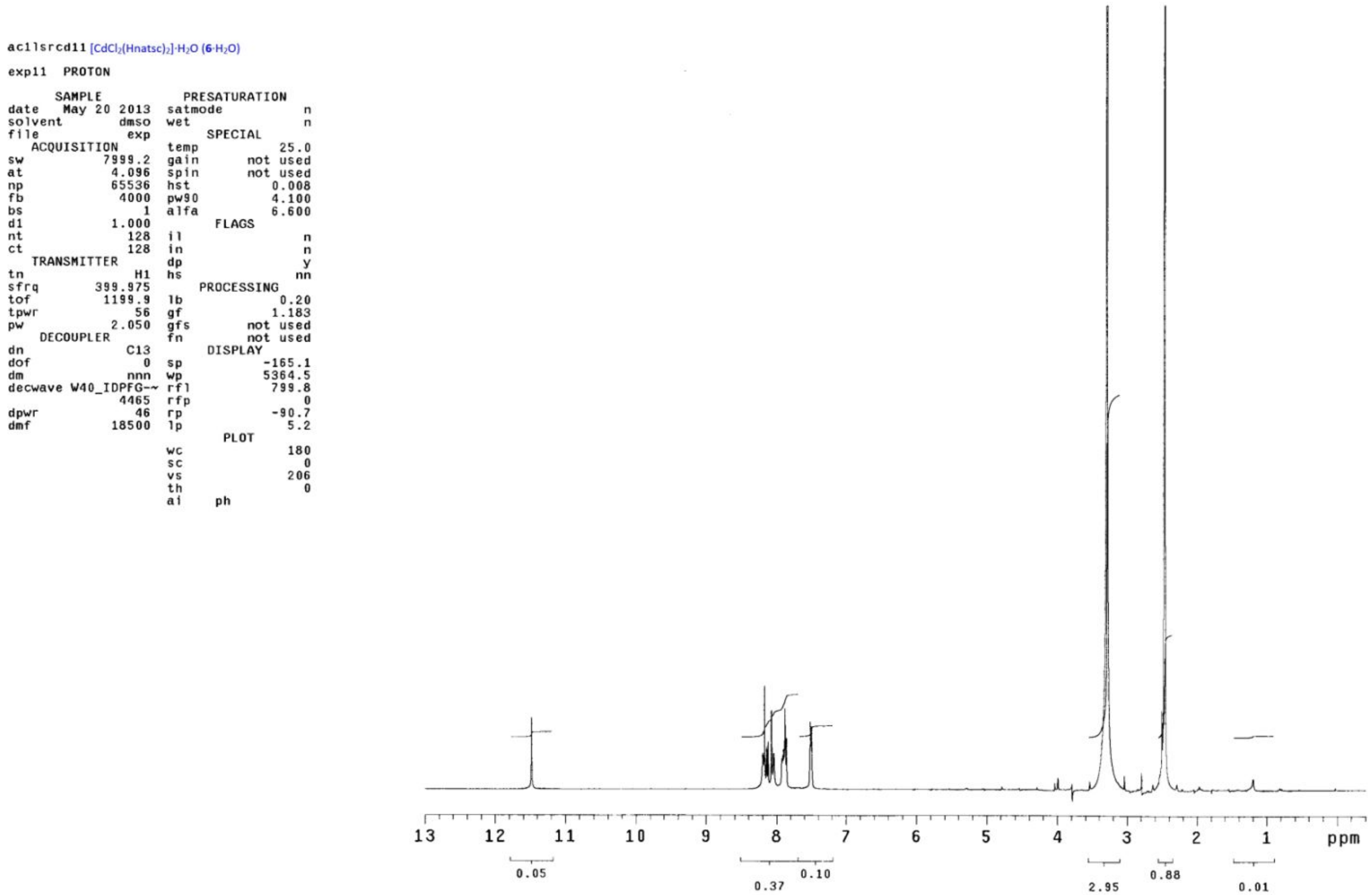

Figure S29. ^{1}H NMR spectrum of **6** in DMSO-d^6 (300 MHz).

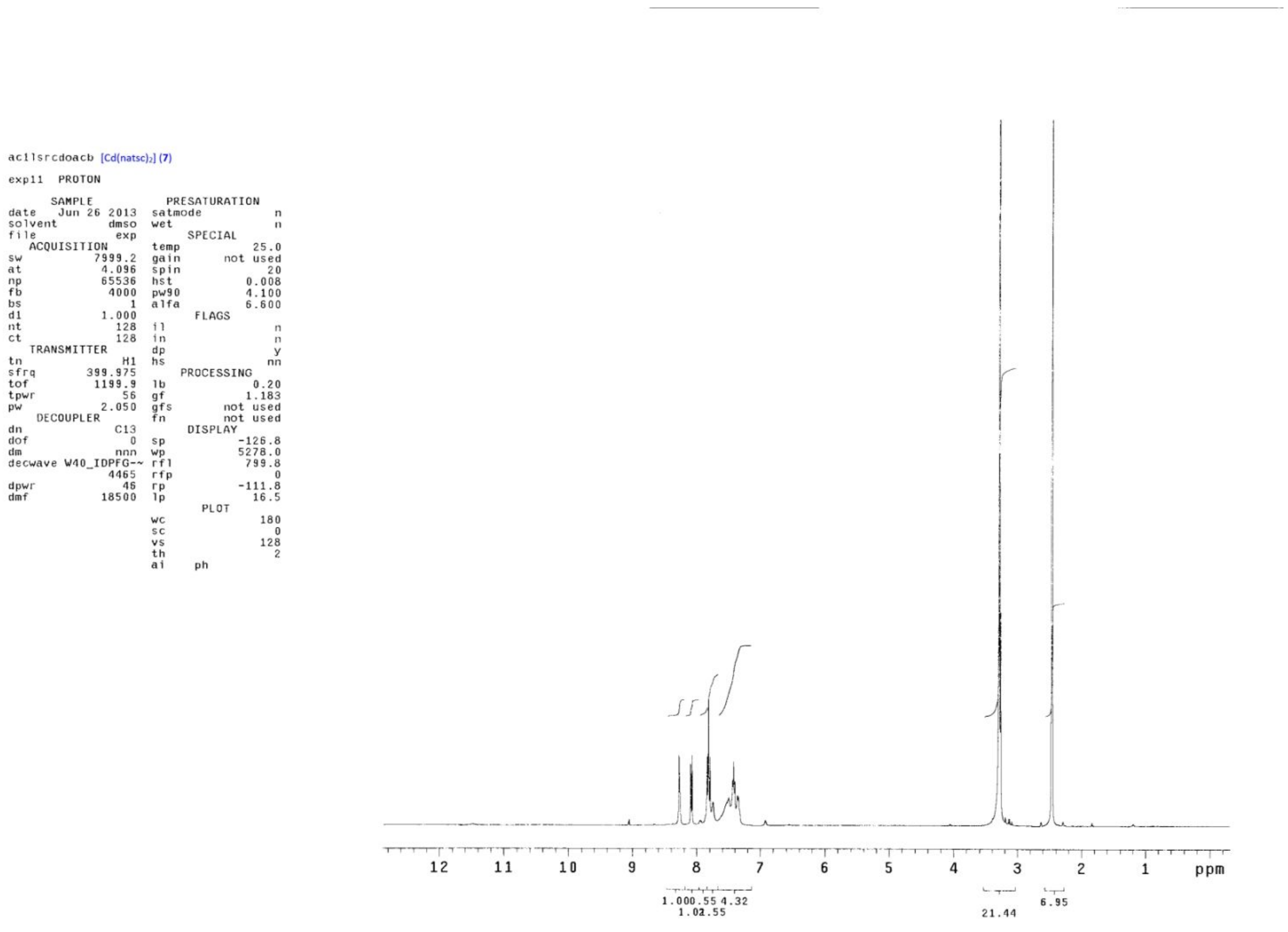

Figure S30. ^{1}H NMR spectrum of **7** in DMSO-d^6 (300 MHz).

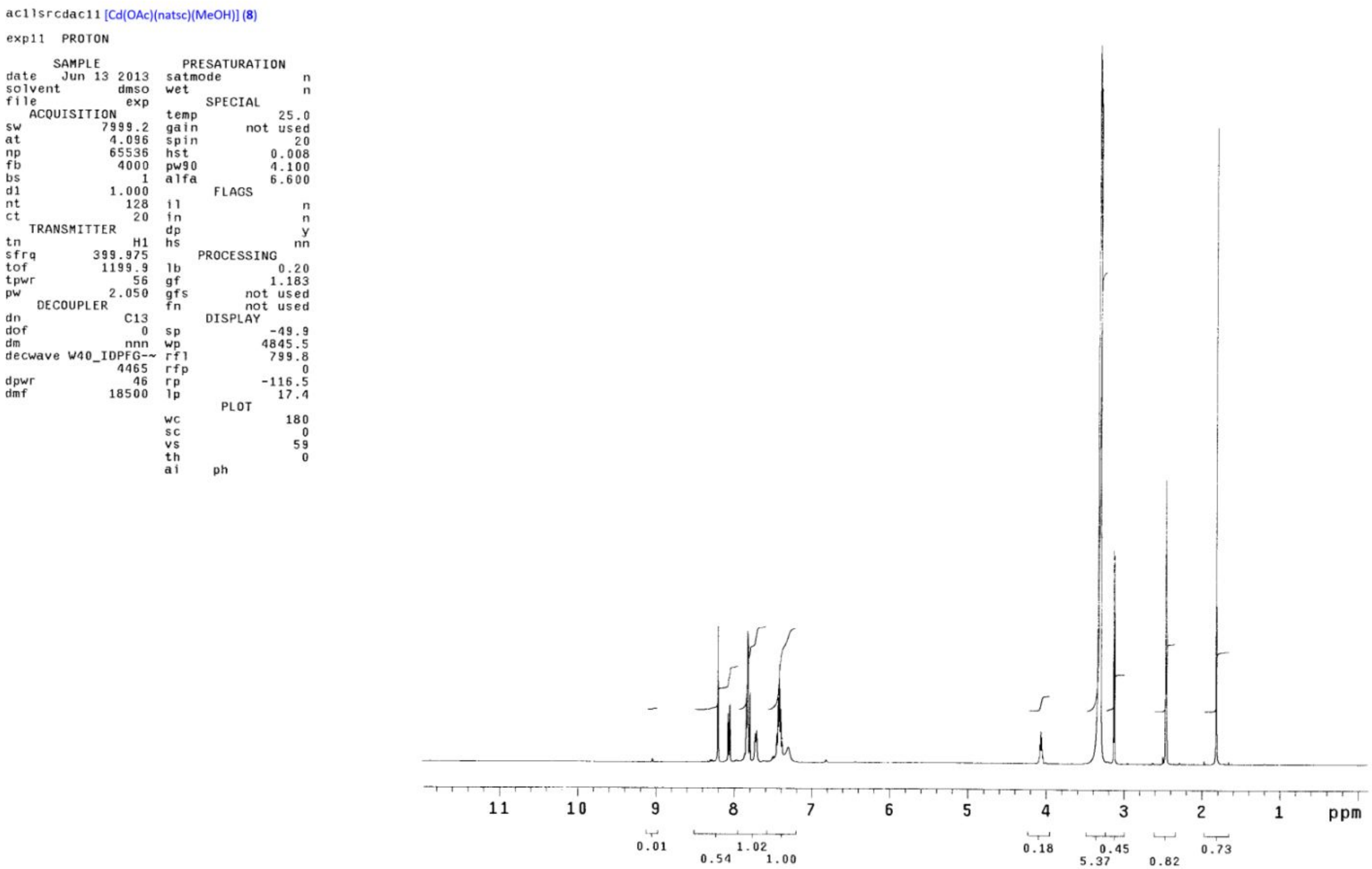

Figure S31. ^{1}H NMR spectrum of **8** in DMSO-d^{6} (300 MHz).

$^{13}C\{^1H\}$ NMR Spectra

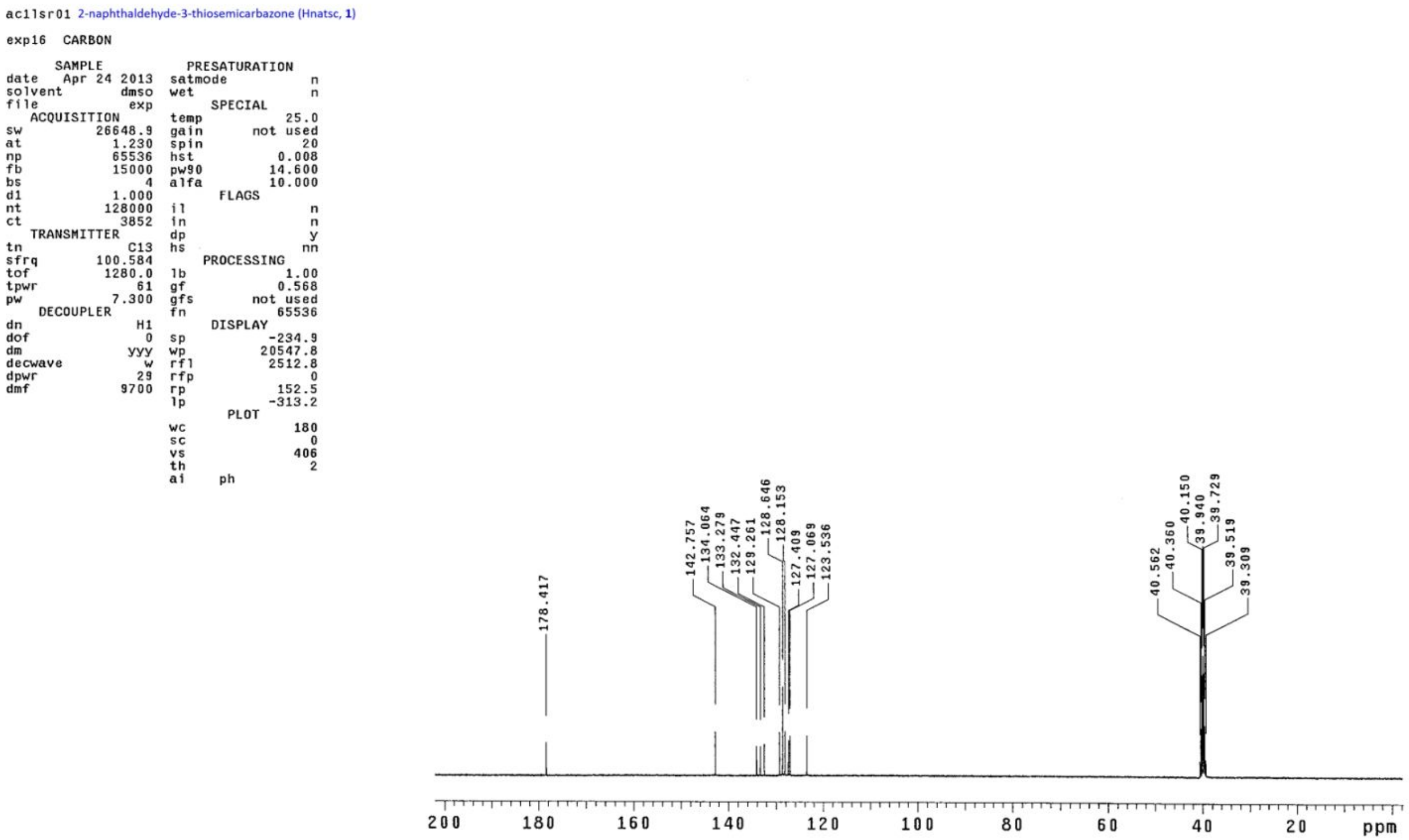

Figure S32. $^{13}C\{^{1}H\}$ NMR spectrum of **1** in DMSO-d6 (300 MHz).

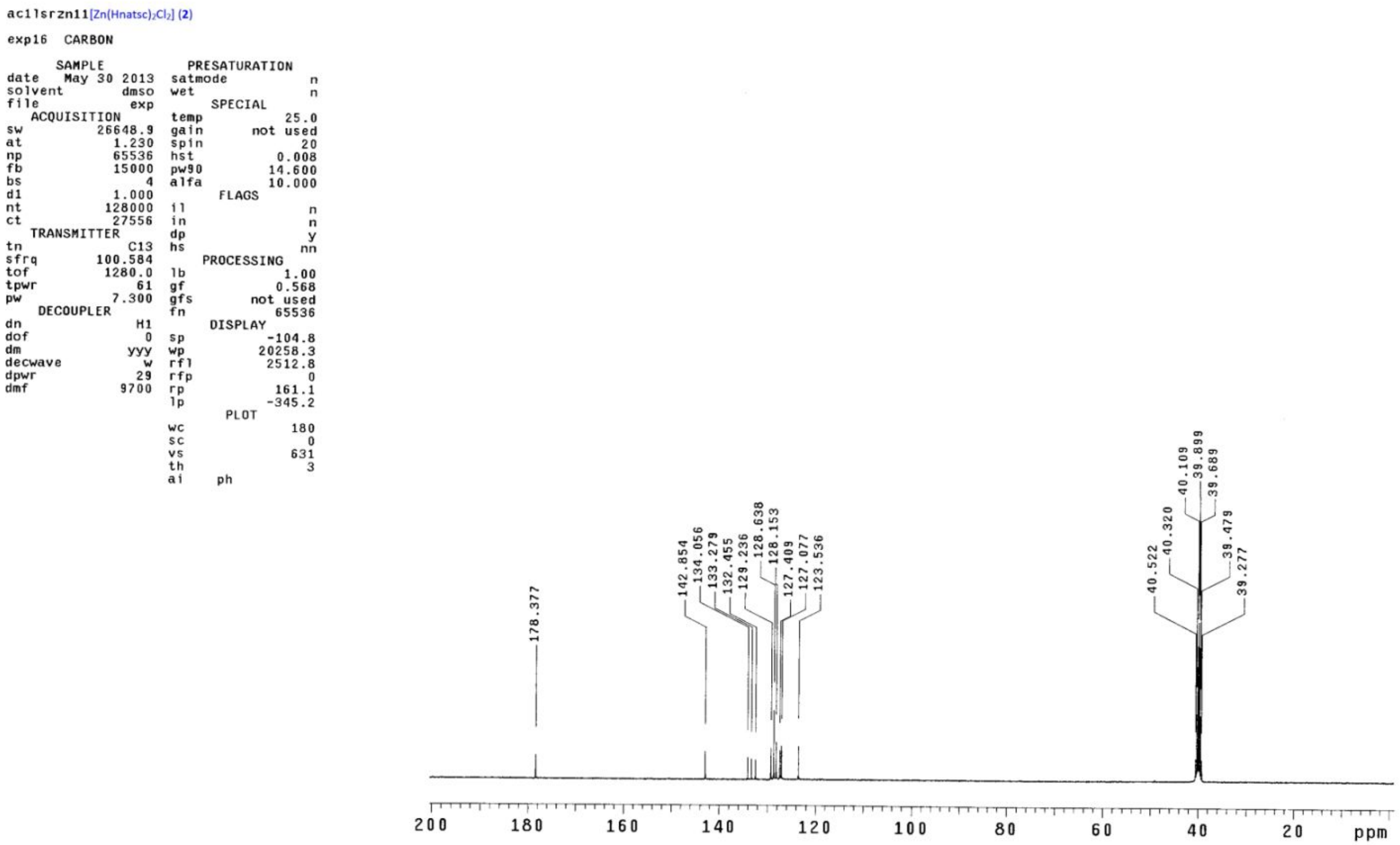

Figure S33. $^{13}C\{^{1}H\}$ NMR spectrum of **2** in DMSO-d^{6} (300 MHz).

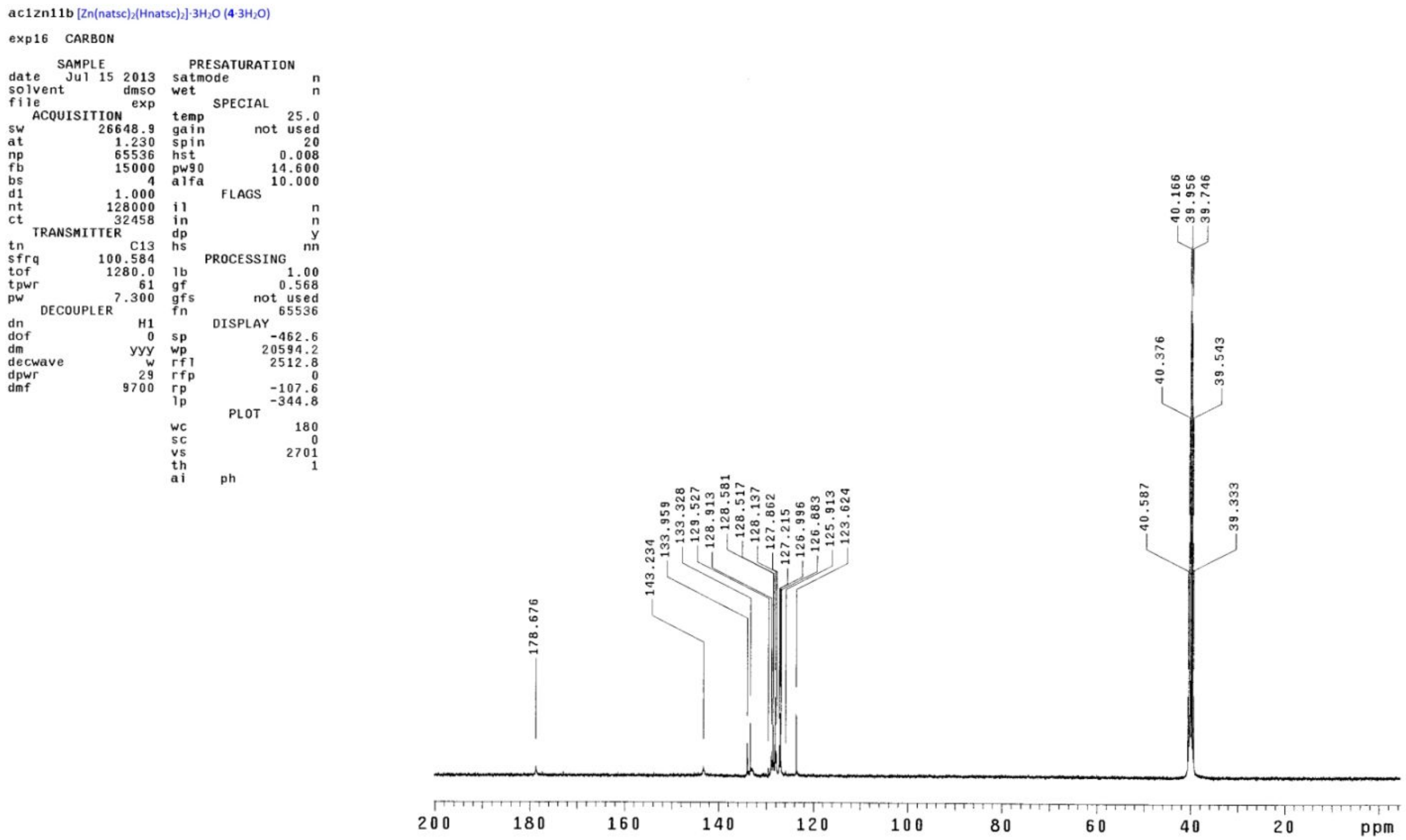

Figure S34. $^{13}C\{^{1}H\}$ NMR spectrum of **4** in DMSO-d^6 (300 MHz).

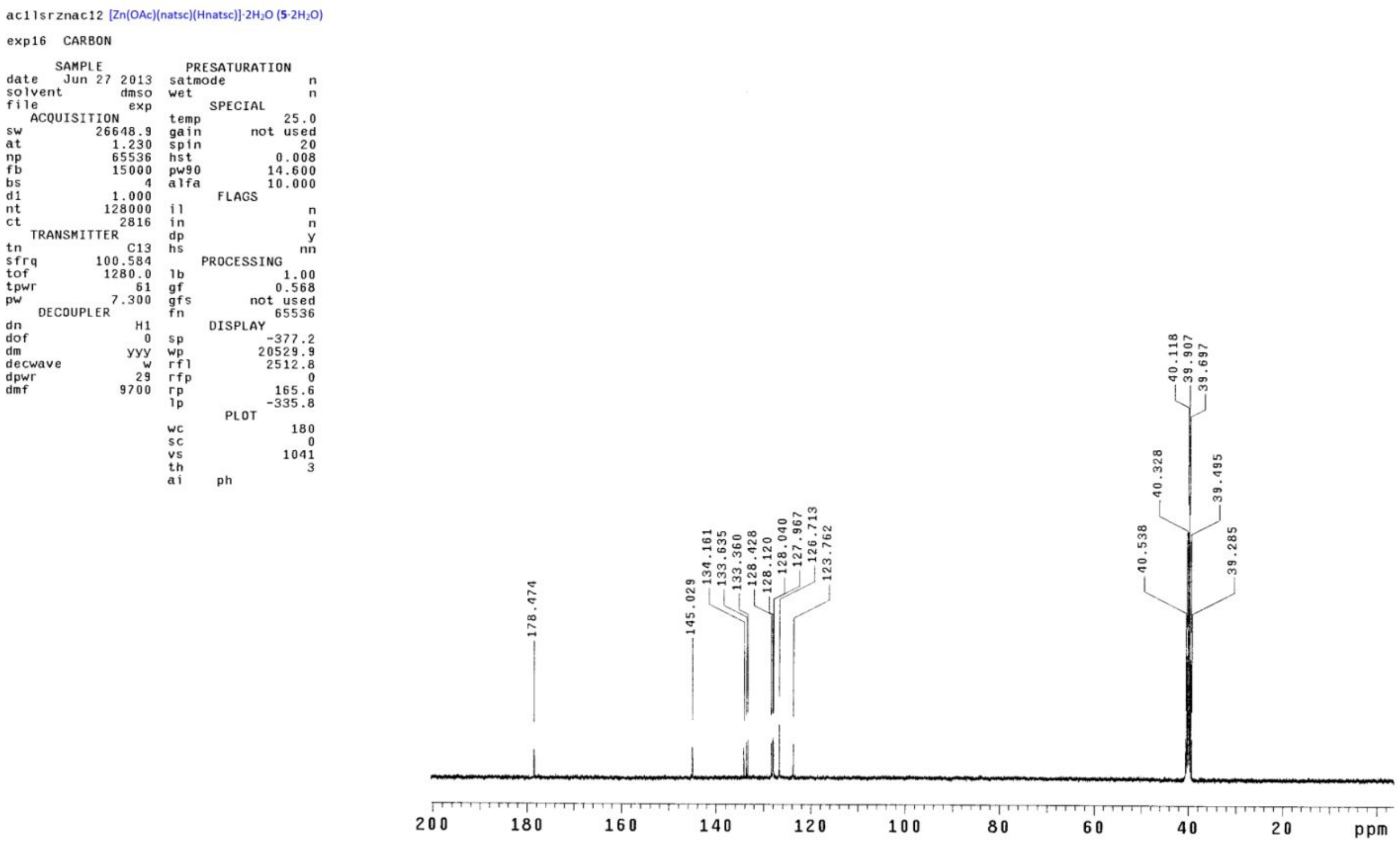

Figure S35. $^{13}C\{^1H\}$ NMR spectrum of **5** in DMSO-d^6 (300 MHz).

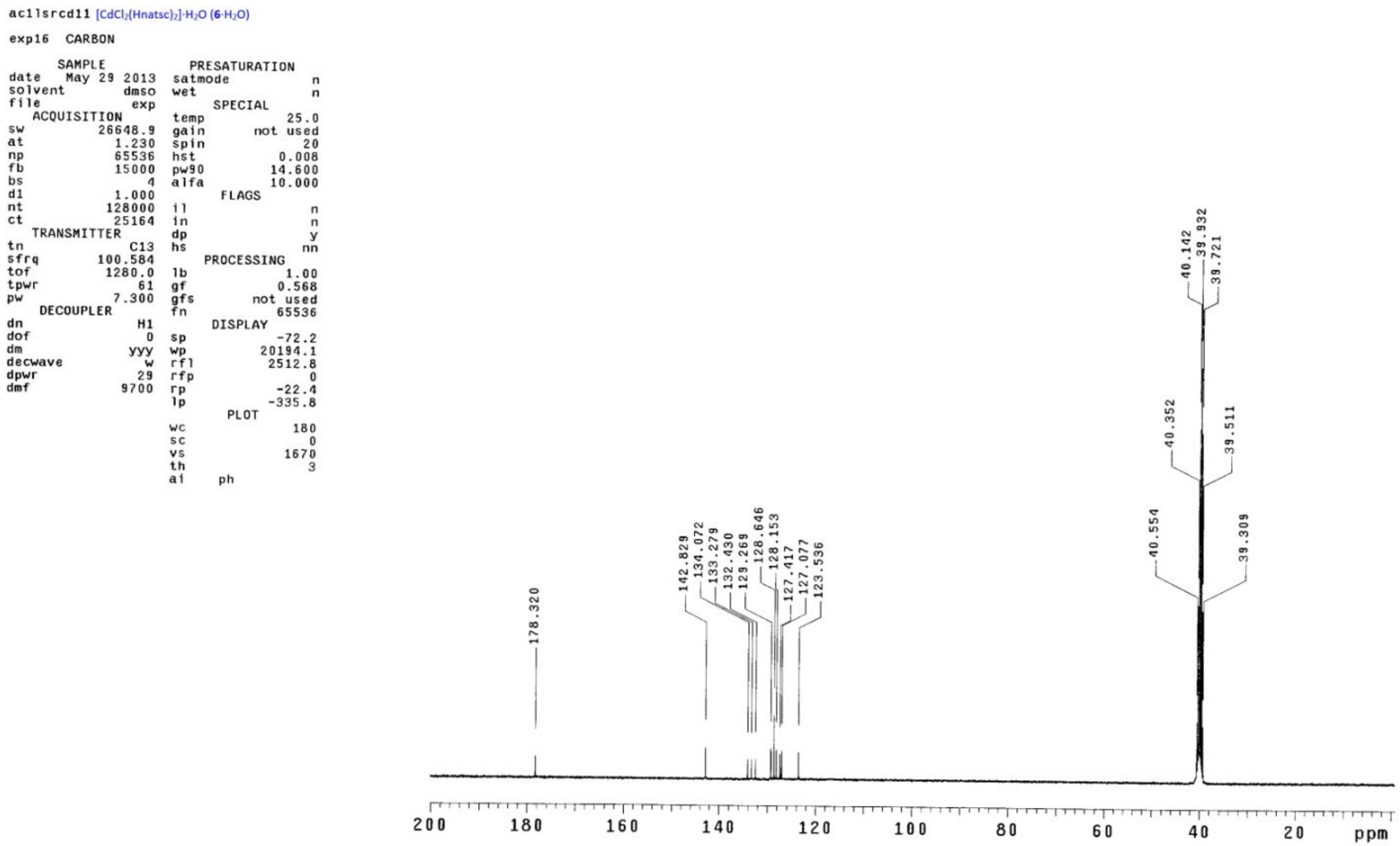

Figure S36. $^{13}C\{^1H\}$ NMR spectrum of **6** in DMSO-d^6 (300 MHz).

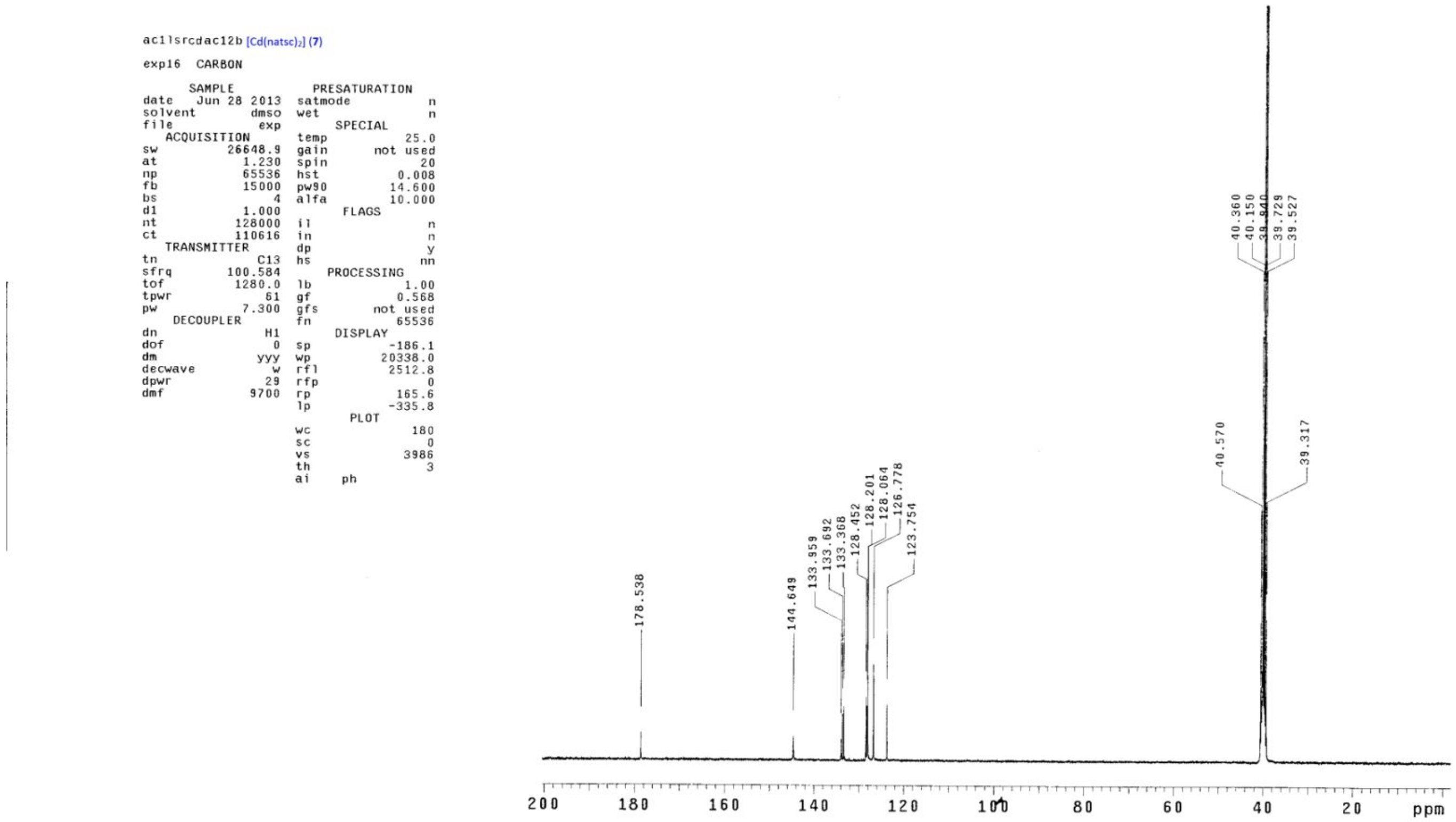

Figure S37. $^{13}C\{^{1}H\}$ NMR spectrum of **7** in DMSO-d^{6} (300 MHz).

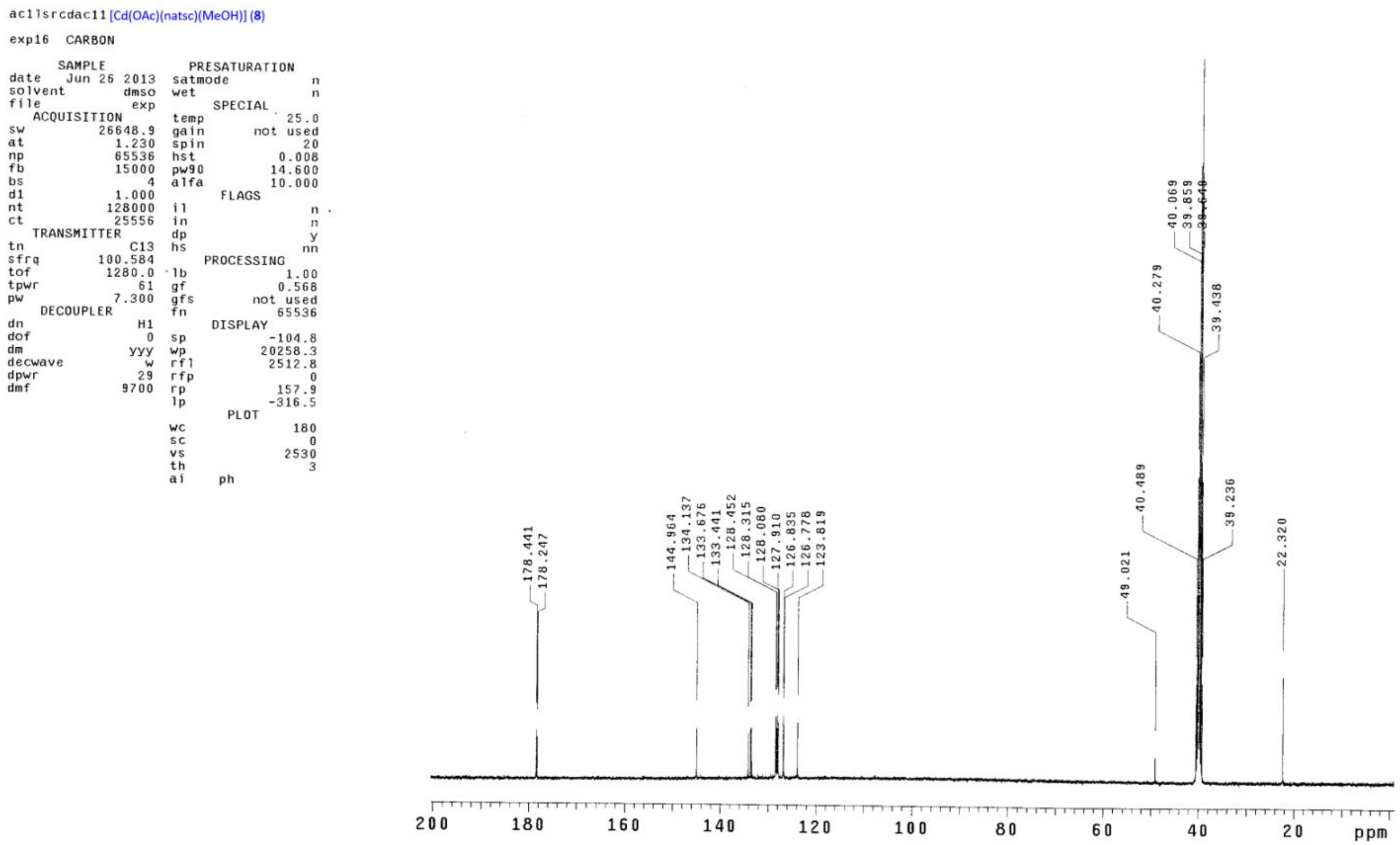

Figure S38. $^{13}C\{^{1}H\}$ NMR spectrum of **8** in DMSO-d[6] (300 MHz).

Crystallography

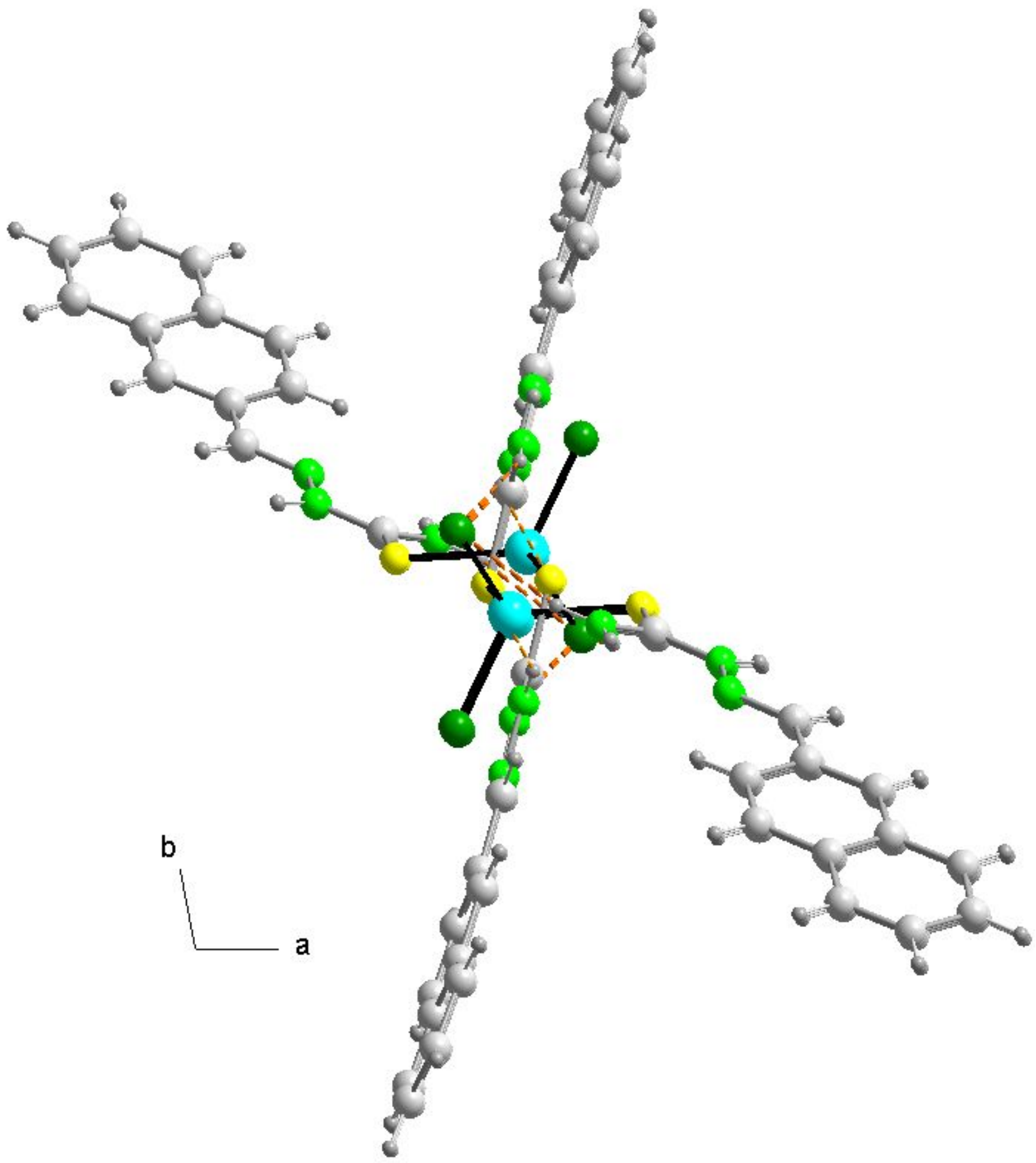

Figure S39. Crystal packing diagram for **2** showing the molecular arrangement along the c-axis, forming a four-spoke helical chain.

Fluorescence Spectra

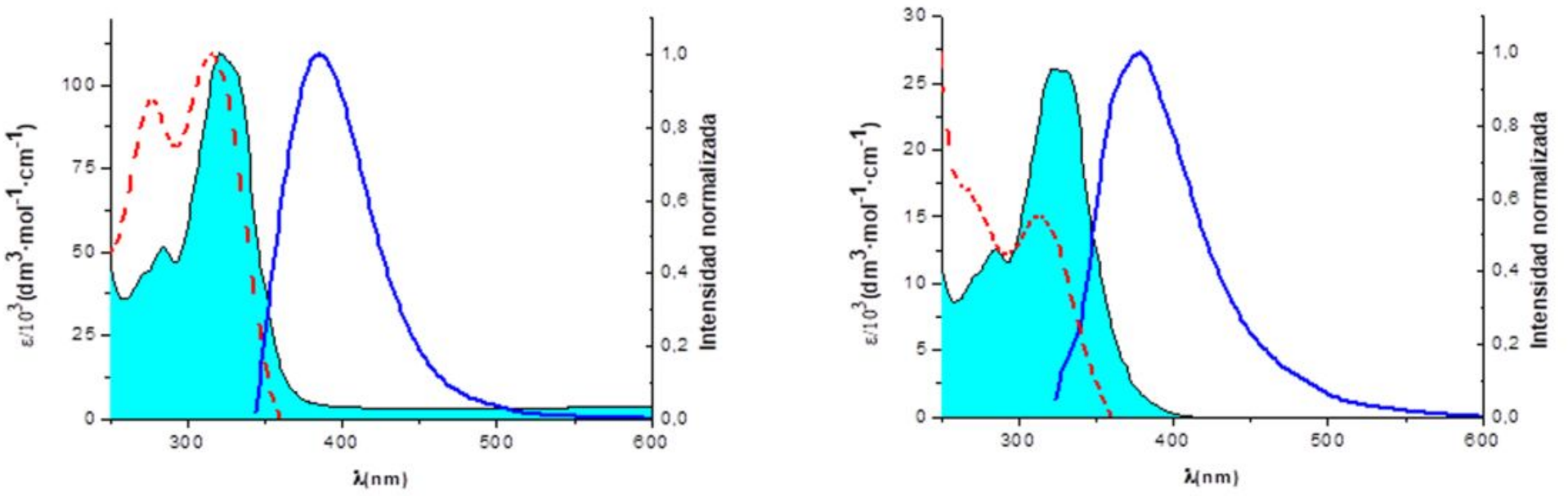

a **b**

Figure S40. Absorption (cyan shaded), excitation (dashed line) and emission (solid line) spectra of a) Zn(OAc)(natsc)(Hnatsc)]·$2H_2O$ (**5**·$2H_2O$) and b) [Cd$(natsc)_2$] (**7**)
